# Genome-wide comparative analyses of GATA transcription factors among 19 *Arabidopsis* ecotype genomes: Intraspecific characteristics of GATA transcription factors

**DOI:** 10.1371/journal.pone.0252181

**Published:** 2021-05-26

**Authors:** Mangi Kim, Hong Xi, Jongsun Park

**Affiliations:** 1 InfoBoss Inc., Gangnam-gu, Seoul, Republic of Korea; 2 InfoBoss Research Center, Gangnam-gu, Seoul, Republic of Korea; Birla Institute of Technology and Science, INDIA

## Abstract

GATA transcription factors (TFs) are widespread eukaryotic regulators whose DNA-binding domain is a class IV zinc finger motif (CX_2_CX_17-20_CX_2_C) followed by a basic region. Due to the low cost of genome sequencing, multiple strains of specific species have been sequenced: e.g., number of plant genomes in the Plant Genome Database (http://www.plantgenome.info/) is 2,174 originated from 713 plant species. Thus, we investigated GATA TFs of 19 *Arabidopsis thaliana* genome-widely to understand intraspecific features of *Arabidopsis* GATA TFs with the pipeline of GATA database (http://gata.genefamily.info/). Numbers of GATA genes and GATA TFs of each *A*. *thaliana* genome range from 29 to 30 and from 39 to 42, respectively. Four cases of different pattern of alternative splicing forms of GATA genes among 19 *A*. *thaliana* genomes are identified. 22 of 2,195 amino acids (1.002%) from the alignment of GATA domain amino acid sequences display variations across 19 ecotype genomes. In addition, maximally four different amino acid sequences per each GATA domain identified in this study indicate that these position-specific amino acid variations may invoke intraspecific functional variations. Among 15 functionally characterized GATA genes, only five GATA genes display variations of amino acids across ecotypes of *A*. *thaliana*, implying variations of their biological roles across natural isolates of *A*. *thaliana*. PCA results from 28 characteristics of GATA genes display the four groups, same to those defined by the number of GATA genes. Topologies of bootstrapped phylogenetic trees of *Arabidopsis* chloroplasts and common GATA genes are mostly incongruent. Moreover, no relationship between geographical distribution and their phylogenetic relationships was found. Our results present that intraspecific variations of GATA TFs in *A*. *thaliana* are conserved and evolutionarily neutral along with 19 ecotypes, which is congruent to the fact that GATA TFs are one of the main regulators for controlling essential mechanisms, such as seed germination and hypocotyl elongation.

## Introduction

Due to the rapid development of sequencing technologies, many sequencing techniques beyond Sanger sequencing, called as next generation sequencing (NGS) technologies, have been established and commercialized [1–3]. Among them, sequencers made by Illumina (HiSeq/NovaSeq) are one of the major sequencing platforms frequently used, producing a huge number of raw reads of which length is 151 bp maximumly with extremely low cost [4,5]. From the first phase of NGS technologies, it promoted whole genome sequencing projects with the aid of a new algorithm of genome assembly, *de bruijn* algorithm [6–11]. As an example, the cucumber genome, the first plant genome assembled from Illumina data, was successfully published in 2009 [12]. After that, many plant genomes have been sequenced with NGS technologies including third generation technology, such as PacBio. It guaranteed much longer contig sequences than those from Illumina data once enough amount of DNA (from 8 to 16 ug) containing long read DNA can be prepared [13].

These new sequencing technologies have resulted in lower sequencing costs, which have changed the trends of whole genome projects: one is increasing number of academically valuable whole genomes [14–17] which provide interesting insights to understand the evolutionary history of plants, beyond economically important species. Another is deciphering many genomes of various strains in one species to identify genetic variations at an intraspecific level [18–25]. The other is genome-wide association studies that investigate genetic variants identified from a large number of individuals’ genomes to find the relationship between genotypes and phenotypes [26–28]. In addition, whole genome sequencing is performed for high-throughput genotyping [29–31].

This trend has uncovered genome-wide sequence variations, including single nucleotide polymorphisms, insertions and deletions, and copy number variations, to find disease-related sequence variations on human for developing individual-specific medicines [32–35], to illuminate evolutionary histories inside species [20], to map biological features to specific variations [24,36,37], or to develop molecular markers to distinguish the origin of species [26,29,38]. Till now more than 10,000 human genomes re-sequenced [39–49] as well as more than 1,700 *A*. *thaliana* [5,18–20,50–53] and 4,000 rice genomes [26,29,54–61] are available. Moreover, the current release of the Plant Genome Database (http://www.plantgenome.info; Park et al., in preparation) [62,63] presents that 103 plant species have more than one whole genome sequences, reflecting that resequencing of additional cultivars or individuals is a recent trend of plant genome projects. However, due to technical reasons, most of the resequenced genomes are usually not provided as assembled sequences as well as do not contain gene models (e.g., *Oryza sativa* [21] and *Populus trichocarpa* [24]), which is a huddle to investigate variations of gene families in detail.

A transcription factor (TF) is a protein that controls the rate of transcriptions by binding to specific DNA sequences including promoter regions of a certain gene. Plant TF plays important roles such as controlling flower developments [64], circadian clock [65], carbon and nitrogen regulatory network [66], and disease resistance [67].

Plant GATA TF family, which is one of the major TF families in plant species [68–72], contains one or sometimes more highly conserved type IV zinc finger motifs (CX_2_X_18,20_CX_2_C) followed by a basic region that can bind to a consensus sequence (WGATAR; W = T or A; R = G or A) [73–75]. Because *Arabidopsis* is a model plant, the biological functions of many GATA TFs have been characterized. For example, AtGATA8 (BME3) is a positive regulator of *Arabidopsis* seed germination [76], AtGATA18 (HAN) is required to position proembryo boundary in the early embryo of *Arabidopsis* [77], and AtGATA25 (ZIM) is involved in hypocotyl and petiole elongation [78].

Even though many genome-wide identifications of GATA TFs in plant species [73,79–87], there is no investigation of intraspecific variations of GATA TFs, which may be fundamental data for understanding subtle differences among natural isolates. Fortunately, the genome project of resequencing *A*. *thaliana* with Illumina technology provided a gene model of 18 *A*. *thaliana* genomes [52]. In addition, reinvestigation of *A*. *thaliana* GATA TFs is also needed because the previous research of genome-wide GATA TF identification was conducted in 2004 [73], when the version gene model of *A*. *thaliana* was older than the current version (TAIR 10.1) [88]. Taken together, we investigated GATA TFs from 19 *A*. *thaliana* genomes including reference genome (*A*. *thaliana* Col0) and analyzed them in the aspects of intraspecific variations of chromosomal distribution, amino acid sequences, and phylogenetic relationships.

Along with 19 *A*. *thaliana* natural isolate genomes, the number of GATA genes and GATA TFs per genome range from 29 to 30 and from 39 and 42, respectively, presenting differences among 19 *A*. *thaliana*. Four genome-wide distribution patterns of GATA TFs were identified. Besides type IV_b_ and IV_c_ defined in previous studies [75,89], an additional type, CX_4_CX_18_CX_2_C (in AtGATA29), named as type IV_4,_ was rescued. Two alternative splicing forms, AtGATA11a and AtGATA15b, were identified only in one *A*. *thaliana* genome, Col0 and Kn0, respectively. In detail, 22 out of 2,195 amino acid positions (1.002%) from 13 out of 41 conserved GATA TFs (31.71%) display amino acid variations across 19 *A*. *thaliana* genomes. 15 out of 30 *A*. *thaliana* GATA genes (50.00%) have been studied about theirs biological functions. Interestingly, GATA genes in subfamily II including seven characterized GATA genes presented the largest amino acid variations implying subtle variations of biological functions across natural isolates of *A*. *thaliana*. Chromosomal distributions of GATA genes on 19 *A*. *thaliana* genomes display biased distribution. PCA results based on 28 characteristics of GATA genes present four groups, same to those defined by the number of GATA genes. Topologies of bootstrapped phylogenetic trees of *Arabidopsis* chloroplast genomes and GATA genes are mostly incongruent and no relationship between geographical distribution and their phylogenetic relationships. Our genome-wide identification of GATA genes in 19 *A*. *thaliana* provides diverse characteristics of intra-species variations of GATA TFs.

## Material and methods

### Collection and preprocess of 19 *Arabidopsis* genome sequences

We utilized nineteen *A*. *thaliana* genomes sequences deposited from the Plant Genome Database (Release 2.6; http://www.plantgenome.info/; Park et al, in preparation) [62,63], which collected genome sequences from several repositories including the NCBI genome database (http://genome.ncbi.nlm.nih.gov/) and standardized based on the GenomeArchive^®^ (http://www.genomearchive.info/; Park et al, in preparation) [90]. We used the gene models of nineteen *Arabidopsis* genomes [52] for systematic studies.

### Identification of GATA TFs from 19 *Arabidopsis* whole genome sequences

Amino acid sequences from nineteen *A*. *thaliana* genomes were subjected to InterProScan [91] to identify GATA TFs. The pipeline for identifying *A*. *thaliana* GATA TFs implemented at the GATA Database (http://gata.genefamily.info/; Park et al., in submission), which is an automated pipeline for identifying GATA TFs with GATA DNA-binding motif InterPro term (IPR000679) and post process to filter out false positive results and for analyzing various analyses including domain sequence analysis, gene family analysis, as well as phylogenetic analysis. GATA Database was constructed and maintained as one of the members of the Gene Family Database (http://www.genefamily.info/; InfoBoss, Inc.; Park et al., in preparation).

### Investigation of exon structure and alternative splicing forms of GATA TFs

Based on the Plant Genome Database (http://www.plantgenome.info/; Park et al., in preparation) [62,63], exon structure and alternative splicing forms of GATA TFs were retrieved. Diagrams of exon structure and alternative splicing forms of GATA TFs were drawn primarily based on the diagram generated by the GATA Database (http://gata.genefamily.info; Park et al., in preparation) with adding additional information manually.

### Assembly of complete chloroplast genomes of *A*. *thaliana* based on public NGS raw reads

Raw sequences downloaded from NCBI SRA (S1 Table) were used for chloroplast *de novo* genome assembly with Velvet v1.2.10 [7] after filtering raw reads using Trimmomatic v0.33 [92]. After obtaining the first draft of the chloro-plast genome sequences, gaps were filled with GapCloser v1.12 [93] and all bases from the assembled sequences wereconfirmed by checking each base in the alignment (view mode in SAMtools 1.9 [94]) against the assembled chloro-plast genome generated with BWA v0.7.17 [95]. All these bio-informatic processes were conducted under the environment of Genome Information System (GeIS; http://geis.infoboss.co.kr/; Park et al., in preparation).

### Construction of phylogenetic tree of GATA TFs

Phylogenetic tree based on amino acid sequences of GATA domains was constructed with neighbor joining (NJ) method (bootstrap repeat is 10,000) by MEGA X [96] based on sequence alignment calculated by ClustalW 2.1 [97] under the environment of the GATA Database (http://gata.genefamily.info/; Park et al., in preparation). For drawing phylogenetic trees based on complete chloroplast genomes, we used MAFFT v7.450 [98] for aligning 19 complete chloroplast genomes including that of *A*. *lyrata* and drew a neighbor-joining phylogenetic tree with 10,000 bootstrap repeats using MEGA X [96], the maximum-likelihood phylogenetic tree with 1,000 bootstrap repeats using IQ-TREE v1.6.2 [99], and Bayesian inference tree (number of generations is 1,100,000) using MrBayes v3.2.7 [100].

## Results

### Identification of GATA TFs from 19 *A*. *thaliana* genomes

We identified 566 GATA genes (773 GATA TFs) from 19 *A*. *thaliana* genomes available in public using the pipeline of GATA database (http://gata.genefamily.info/; Park et al., in preparation; Table 1 and S2 Table). Gene models of 19 *A*. *thaliana* genomes contain alternative splicing forms, so that numbers of GATA TFs are larger than those of GATA genes (Table 1), presenting potential functional differentiation of GATA TFs: e.g. expression levels of alternative forms of one GATA gene (OsGATA23) are different in the same condition [101]. Numbers of GATA genes and GATA TFs of each *A*. *thaliana* genome range from 29 to 30 and 39 to 42, respectively (Table 1). The absence and presence of the AtGATA24 gene in each *A*. *thaliana* genome caused the differences of the number of GATA genes (Table 1). Its function is controlling cryptochrome1-dependent response to excess light [102]. The existence of AtGATA24 homologs in *Arabidopsis lyrata* (EFH59549.1 and EFH67905.1) and *Arabidopsis halleri* (Araha.17146s0001.1 and Araha.2389s0021.1) genomes identified using BLAST search (S1 Fig) indicates that four accessions which do not contain AtGATA24 might miss this gene due to assembly errors.

**Table 1:** Summary of identified GATA TFs from 19 *A*. *thaliana* genomes.

*A*. *thaliana* genome names	# of GATA genes	# of GATA TFs	# of genes	# of proteins
Col0	30	42	27,949	48,147
Edi0	30	41	26,997	38,813
Ct1	30	41	27,006	38,930
Can0	30	41	26,949	38,556
Bur0	30	41	27,014	38,717
Hi0	29	39	27,052	39,015
Kn0	30	42	27,002	38,908
Ler0	29	39	27,014	38,997
Mt0	29	39	27,002	38,685
No0	30	41	27,018	38,635
Oy0	30	41	27,010	38,596
Po0	30	41	27,045	38,776
Rsch4	30	41	27,031	38,557
Sf2	30	41	26,974	38,513
Tsu0	30	41	27,013	38,701
Wil2	30	41	26,978	38,558
Ws0	29	39	27,010	38,395
Wu0	30	41	27,024	38,704
Zu0	30	41	27,044	38,901
**Total**	**566**	**773**	**514,132**	**745,104**

**Fig 1:**
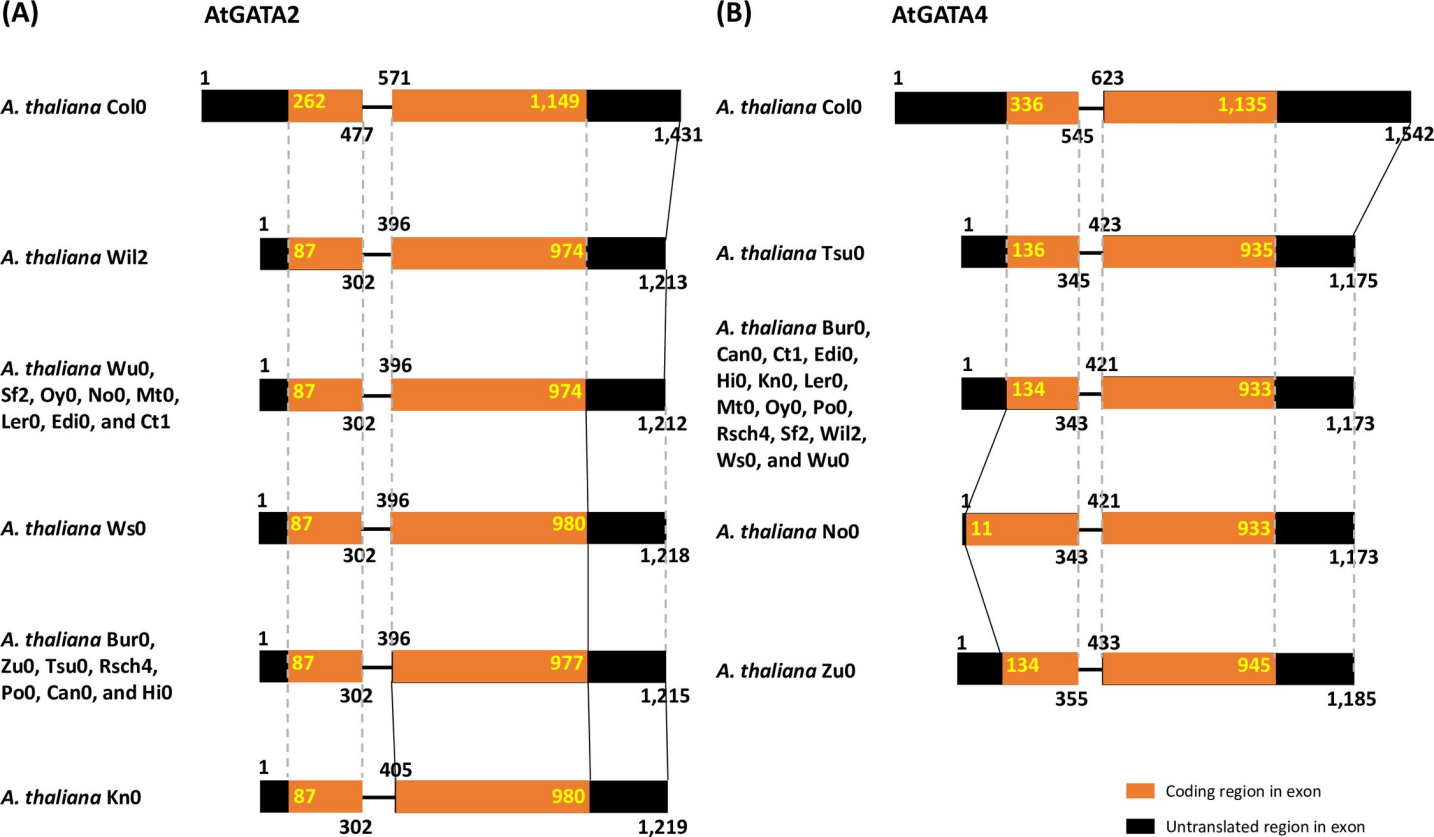
Gene structure of AtGATA2 and AtGATA4 in 19 *A*. *thaliana*. (A) shows gene structure of AtGATA2 genes from 19 *A*. *thaliana* genomes. (B) displays gene structure of AtGATA4 genes from 19 *A*. *thaliana* genomes. Yellow boxes indicate translated regions and black boxes display untranslated regions. Numbers around boxes display relative positions of translated, untranslated, and exons. Names of *A*. *thaliana* genomes are printed in the left part of each gene diagram. Dotted and solid lines indicate the conserved and different structure of GATA genes including exon, intron, and untranslated regions, respectively.

The conserved GATA genes from 19 *A*. *thaliana* genomes, such as AtGATA2 and AtGATA4, presented various exon structures along with *A*. *thaliana* genomes (Fig 1). Lengths of 5’ untranslated regions (UTRs) of AtGATA2 and AtGATA4 gene are different from each other, ranging from 86 bp (18 genomes except Col0) to 261 bp (Col0; Fig 1A) and 10 bp (No0) to 335 bp (Col0; Fig 1B), respectively. In addition, the first and second exons of both GATA genes along with nineteen *A*. *thaliana* genomes show slightly different lengths (Fig 1). Finally, 3’ and 5’ UTRs of both genes also present differences (Fig 1). Interestingly, the Col0 genome displays longer UTRs in comparison to the remaining ecotypes (Fig 1). These variations of exon and intron structure including UTRs were also identified in the other gene families, including polyol transporter [103] and Lipocalin [104] gene families. Even though previous studies display inter-species variations of exon-intron structure in the gene family, they support that these intraspecific variations of the GATA TF family can be considered as fundamental data to understand microevolutionary mechanisms in the gene family, especially for TF families.

### Alternative splicing forms of GATA genes from 19 *A*. *thaliana* genomes

**Fig 2:**
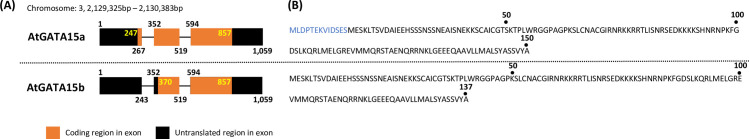
Gene structure and protein sequence of alternative splicing forms of AtGATA15 gene in *A*. *thaliana* Kn0. (A) shows gene structure of two alternative splicing forms of AtGATA15 gene in *A*. *thaliana* Kn0 genome. Black- or orange-colored boxes indicate untranslated and coding regions in exons, respectively. Black lines mean intron regions. Numbers around exon boxes present relative base pair position started from a transcript start position of the AtGATA15 gene. The chromosomal position of the AtGATA15 gene is displayed on the top of the diagram. (B) exhibits protein sequences of alternative splicing forms of the AtGATA15 gene. Black dots with numbers present the position of amino acids. The amino acids marked in blue letters indicate AtGATA15a specific amino acids.

**Fig 3:**
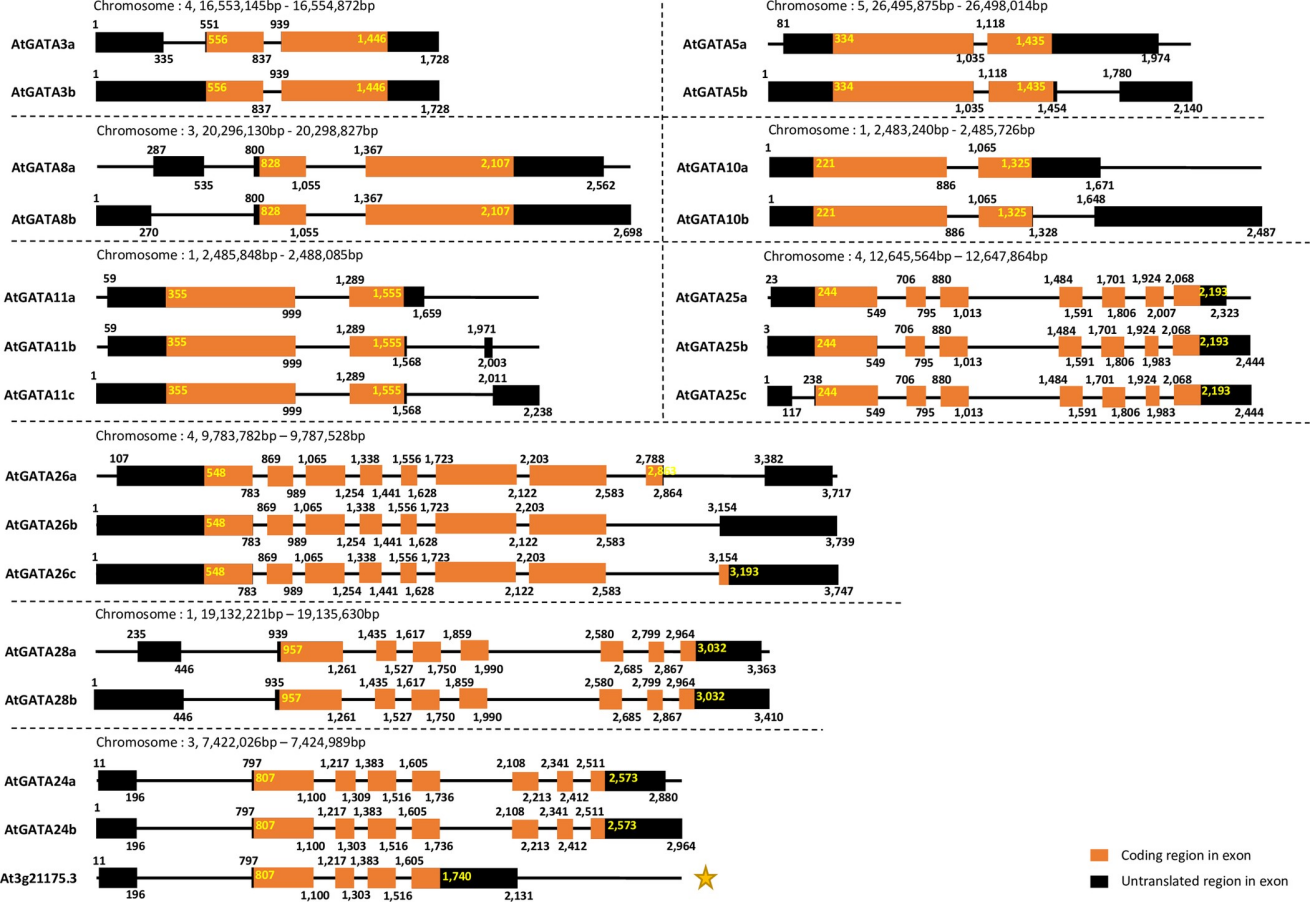
Gene structure of alternative splicing forms of GATA genes in *A*. *thaliana* Col0. It shows alternative splicing forms of GATA genes in *A*. *thaliana* Col0. Black and orange color thick boxes indicate exons and lines means intron. Black- or orange-colored boxes indicate untranslated and coding regions in exons, respectively. Numbers around exon boxes present relative base pair position started from a transcript start position of each gene. Yellow star indicates one of the alternative splicing forms of GATA gene without GATA domain.

The Numbers of GATA genes which have alternative splicing forms range from 8 to 10 per each *A*. *thaliana* genome (see # of GATA genes having alternative splicing forms in Table 2), which account for 29.68% of 566 GATA genes from 19 *A*. *thaliana* genomes (Table 2). The average number of alternative splicing forms of GATA genes for each *A*. *thaliana* genome ranges from 1.34 (*A*. *thaliana* Hi0, Ler0, Mt0, and Ws0) to 1.40 (*A*. *thaliana* Kn0 and Col0; Table 2; Average number of alternative splicing forms of GATA genes). The numbers of total alternative splicing forms of *A*. *thaliana* Kn0 and Col0 GATA genes are the largest among 19 *A*. *thaliana* genomes (Table 2) because AtGATA15 in *A*. *thaliana* Kn0 has two alternative splicing forms and AtGATA11 in *A*. *thaliana* Col0 has three alternative splicing forms; while AtGATA15 of *A*. *thaliana* genomes except *A*. *thaliana* Kn0 has one and AtGATA11 of *A*. *thaliana* genomes except *A*. *thaliana* Col0 has two. Interestingly, translation start positions of two alternative splicing forms of AtGATA15 are different in *A*. *thaliana* Kn0 (Fig 2A), resulting length of amino acids of AtGATA15a is longer than that of AtGATA15b by acquiring MLDPTEKVIDSES (Fig 2B). It is caused by subtle differences in length of the first exon, invoking another start codon in the first exon of AtGATA15a was considered as the start position of this protein. In addition, AtGATA11 of *A*. *thaliana* Col0 presents that the translation start site of three alternative splicing forms are the same to each other; while the transcript start site of AtGATA11c is different from those of AtGATA11a and AtGATA11b (Fig 3). Taken together, the differences identified among 19 ecotyeps, such as number of average alternative splicing forms of each ecotype genome, are caused by the three GATA genes (AtGATA11, AtGATA15, and AtGATA24) implies the importance of GATA TFs in *A*. *thaliana*, such as regulation of seed germination [76].

**Table 2:** Number of GATA genes having alternative splicing forms in 19 *A*. *thaliana* genomes.

*A*. *thaliana* genome	Edi0, Ct1, Can0, Bur0, No0, Oy0, Po0, Rsch4, Sf2, Tsu0, Wil2, Wu0, and Zu0	Hi0, Ler0, Mt0, and Ws0	Kn0	Col0	Total
# of GATA genes (A)	30	29	30	30	566
# of GATA genes having alternative splicing forms (B)	9	8	10	9	168
# of GATA alternative splicing forms from GATA genes containing alternative splicing forms	20	18	22	21	375
Average number of alternative splicing forms of GATA genes	1.37	1.34	1.40	1.40	1.37
**Ratio (B/A)**	**30%**	**27.59%**	**33.33%**	**30%**	**29.68%**

Interestingly, AtGATA11, AtGATA25, and AtGATA26 have three alternative splicing forms, which are the largest number of alternative splicing forms among 19 *A*. *thaliana* genomes (Fig 3). Translated sequences derived from two alternative splicing forms of the AtGATA25 gene (AtGATA25b and AtGATA25c) are 309 aa long, while AtGATA25a is 317 aa (Fig 3). In addition, the numbers of exons of the AtGATA25c are 8 but the rests are 7 (Fig 3). Three alternative splicing forms of AtGATA25 gene present the same start and end positions of ORFs and only the sixth exon from the translation start site shows different lengths: one is 60 bp in length and the other is 84 bp (Fig 3). Three alternative splicing forms of the AtGATA26 gene present different protein lengths, different from those of the AtGATA25 gene; 526 aa (AtGATA26a), 514 aa (AtGATA26c), and 510 aa (AtGATA26b). In addition, AtGATA26a from Hi0 present 515 aa, shorter than those of AtGATA26a from the rest of *A*. *thaliana* genomes. The number of exons of AtGATA26a is 9 and the other two are 8 (Fig 3). Two alternative splicing forms except for AtGATA26a have the same transcription start site, while the transcription end site of the three alternative splicing forms is different from each other (Fig 3). In addition, the eighth exons of the three alternative splicing forms present a different length: that of AtGATA26a is the shortest and that of AtGATA26c is the longest (Fig 3).

The significance of the average number of alternative splicing forms of the GATA gene presents divergence of their biological functions: e.g., OsGATA23 showing different expression levels of different alternative splicing forms [101]. Including this case, we can deduce the several points from the average number of alternative splicing forms of GATA genes: i) differences of start methionine (e.g., AtGATA15) can affect their biological function: mineralocorticoid receptor A and B forms of human which present different transcriptional activities by alternative translation sites [105], ii) exon configuration which shows different exon-intron junctions also affects their functions in the cell: one typical example is OsGATA23 which contains two alternative splicing forms of which numbers of exons and their lengths are different and shows different expression levels for each different alternative splicing form [101]. It indicates that the average number of alternative splicing forms of GATA genes along with subfamilies may reflect subfamily-specific functional diversity.

We also identified that one alternative splicing form (At3g21175.3) of the AtGATA24 gene missed the GATA domain (Fig 3), found in all 15 *A*. *thaliana* genomes except for *A*. *thaliana* Hi0, Ler0, Mt0, and Ws0. Twelve GATA genes from three *Populus* species, *P*. *tremula*, *P*. *tremuloides*, and *P*. *tremula* x *alba* 717-1B4) also miss the GATA domain (Kim et al., in preparation), which is the same phenomenon to that of *A*. *thaliana*. We excluded these GATA TFs without DNA-binding domain for further analyses; however, these GATA TFs without DNA-binding domain can also negatively regulate target transcripts by competing with normal GATA TFs [106] because GATA TFs require additional accessory proteins for regulating target genes. Taken together, an average number of alternative splicing forms along with GATA gene families can be an indicator to show a degree of precise regulation of GATA genes’ functions.

### Identification and characteristics of GATA subfamilies in 19 *A*. *thaliana* genomes

Seven subfamilies of GATA genes were identified based on the most previous studies of the plant GATA gene family [73], among which three (V, VI, and VII) are monocot-specific and the rest four are common. Based on many genome-wide identification studies of GATA genes in plant genomes [73,79–82,84–87], the number of GATA genes in subfamily I has the largest except *Brassica napus* [107] and that of subfamily IV is the smallest in dicot species (Table 3). Interestingly, GATA genes from two more monocot genomes, *Triticum aestivum* [79] and *Phyllostachys edulis* [87] have been identified, presenting that only three or four subfamilies identified from dicots were mentioned (Table 3). Two GATA genes (PeGATA6 and PeGATA11) from *P*. *edulis* and two GATA genes (TaGATA-A2 and TaGATA-A11) from *T*. *aestivum* contain two or three GATA domains [79,87], which should be classified into subfamily VI based on the study of *Oryza sativa* [73], indicating that new criteria for classifying subfamilies of GATA genes should be established again against available hundreds of plant genomes.

**Table 3:** Number of each subfamily of GATA genes in plants analyzed GATA gene family.

Plant genome names	Number of each subfamily of GATA genes							Ref.
	I	II	III	IV	V	VI	VII	
*A*. *thaliana*	14	11*	3	2	0	0	0	[73]
*G*. *max*	30	17	9	8	0	0	0	[80]
*G*. *arboreum*	20	13	8	5	0	0	0	[81]
*G*. *hirsutum*	36	25	16	10	0	0	0	[81]
*G*. *raimondii*	19	14	8	5	0	0	0	[81]
*M*. *domestica*	20	8	4	3	0	0	0	[82]
*R*. *communis*	7	7	4	1	0	0	0	[84]
*S*. *lycopersicum*	14	9	4	3	0	0	0	[85]
*P*. *trichocarpa*	18	10	9	2	0	0	0	[108]
*B*. *napus*	36	43	10	7	0	0	0	[107]
*O*. *pumila*	7	5	5	1	0	0	0	[109]
*V*. *vinifera*	7	6	5	1	0	0	0	[86]
*O*. *sativa*	7	9	5	1	2	3	2	[73]
*T*. *aestivum*	13	6	4	3	0	0	0	[79]
*P*. *edulis*	12	13	6	0	0	0	0	[87]
**Total**	**260**	**196**	**100**	**52**	**2**	**3**	**2**	

*This number is based on our analysis because one GATA gene has been added.

**Piper nigrum, *Zea mays*, *Solanum tuberosum*, and *Capsicum annuum* results were omitted because its paper could not be accessed [83,110–112].

***In the case of two species, different classification, group A, B, C, and/or D, was used so that it is also omitted (group A: 15 GATA genes, group B: 5 GATA genes, group C: 7 GATA genes, and group D: 1 GATA genes in *Brachypodium distachyon* [113] and group A: 17 GATA genes, group B: 5 GATA genes, and group C: 3 GATA genes in *Cicer arietinum* [114]).

There are four types of distribution of GATA TFs along with four subfamilies identified in 19 *A*. *thaliana* genomes (Table 4). The largest one (Type 1), which is from thirteen out of 19 *A*. *thaliana* genomes except for *A*. *thaliana* Col0, Hi0, Ler0, Mt0, Ws0, and Kn0, presents 14 GATA genes (19 GATA TFs) in subfamily I, 11 (11 GATA TFs) in subfamily II, 3 (7 GATA TFs) in subfamily III, and 2 (4 GATA TFs) in subfamily IV (Table 5). The second largest one (Type 2) found in four *A*. *thaliana* genomes, such as Hi0, Ler0, Mt0, and Ws0, shows 2 GATA genes (5 GATA TFs) in subfamily III because of the absence of the AtGATA24 gene. The third type (Type 3) from the *A*. *thaliana* Kn0 genome displays one more GATA TF in subfamily II in comparison to Type 1 because the AtGATA15 gene has one more alternative splicing form than the rest of *A*. *thaliana* genomes. In addition, this additional alternative splicing form is uniquely identified in subfamily II among 19 *A*. *thaliana* genomes. The last form (Type 4) found in *A*. *thaliana* Col0 shows that numbers of GATA TFs except for subfamily I are the same as those of the Type 1; number of GATA TFs in subfamily I of *A*. *thaliana* Col0 is 20 because of AtGATA11a, unique GATA TF among 19 *A*. *thaliana* genomes.

**Table 4:** Number of GATA genes identified from 19 *A*. *thaliana* genomes along with subfamilies.

Type	*A*. *thaliana* genome	# of GATA genes				# of GATA TFs Ratio between GATA genes and TFs			
		I	II	III	IV	I	II	III	IV
Type 1	Edi0, Ct1, Can0, Bur0, No0, Oy0, Po0, Rsch4, Sf2, Tsu0, Wil2, Wu0, and Zu0	14	11	3	2	19	11	7	4
						1.36	1.00	2.33	2.00
Type 2	Hi0, Ler0, Mt0, and Ws0	14	11	2	2	19	11	5	4
						1.36	1.00	2.50	2.00
Type 3	Kn0	14	11	3	2	19	12	7	4
						1.36	1.09	2.33	2.00
Type 4	Col0	14	11	3	2	20	11	7	4
						1.43	1.00	2.33	2.00
**Total**		**266**	**209**	**53**	**38**	**362**	**210**	**125**	**76**

**Table 5:** List of plant GATA TFs including partial type (Type IV_p_).

Plant name	GATA name	Class IV zinc finger motif	Reference
*A*. *thaliana*	AtGATA26a	CX_15_CX_2_C	GATA DB*
*M*. *domestica*	MdGATA27	CX_14_CX_2_C	GATA DB*
*M*. *domestica*	MdGATA35	CX_21_CX_2_C	GATA DB*
*O*. *sativa*	OsGATA24	CX_2_CX_17_	[73]
*G*. *max*	GmGATA28	CX_2_CX_14_	[80]
*G*. *max*	GmGATA48	X_14_CX_2_C	[80]
*P*. *edulis*	PeGATA1	CX_18_CX_2_C	[87]
*P*. *edulis*	PeGATA14	X_17_CX_2_C	[87]
*P*. *edulis*	PeGATA17	CX_2_CX_18_	[87]
*P*. *edulis*	PeGATA18	CX_2_CX_12_	[87]
*P*. *edulis*	PeGATA30	CX_2_CX_18_C	[87]

*GATA transcription factor database, http://gata.genefamily.info/.

Subfamily III shows the highest ratio between GATA TFs and GATA genes, ranging from 2.33 to 2.50 (Table 4); while subfamily II is the lowest (1.00 to 1.09). In subfamily IV, only one of two GATA genes has alternative splicing forms. These results suggest together with the previous studies showing diversified functions of alternative splicing forms of TFs [101,115] that subfamily III may have diverse functions in comparison to the rest of subfamilies. In the case of subfamily II, except *A*. *thaliana* Kn0, there is no alternative splicing form found in *A*. *thaliana* genomes. No alternative splicing form of GATA subfamily II is also found in the recent *Glycine max* genome release of which gene model covers alternative splicing forms. However, four *Populus* genomes (*Populus trichocarpa*, *Populus euphratica*, *Populus tremuloides*, and *Populus tremula* x *alba* 717-1B4) present maximally three alternative splicing forms in subfamily II (Kim et al, in preparation). Taken together, *A*. *thaliana* subfamily II may not be functionally diversified in comparison to *Populus* species [116]. In addition, *O*. *sativa*, a monocot species, also shows that subfamily II contains alternative splicing forms (OsGATA8) [101].

**Fig 4:**
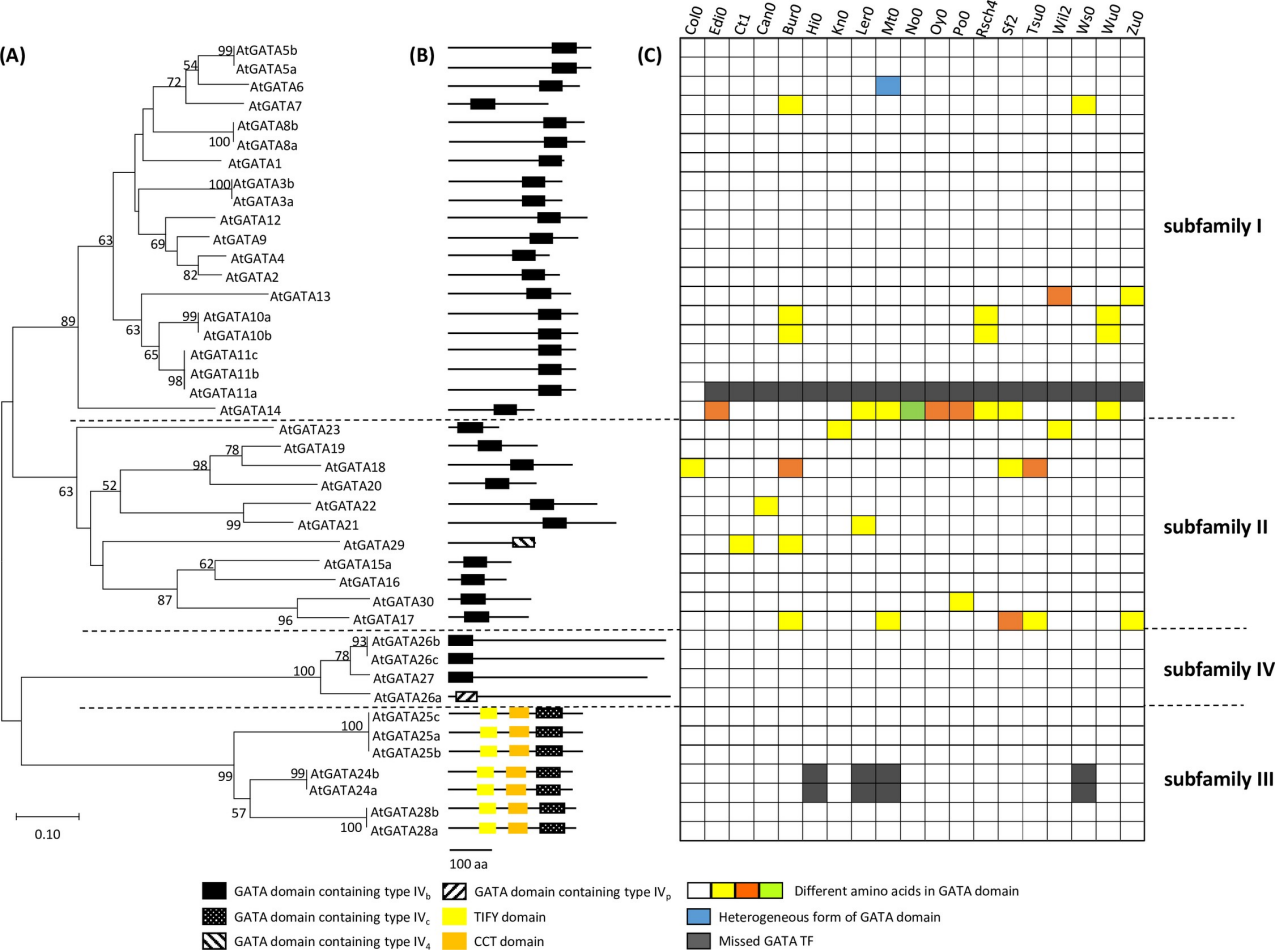
Domain structure in *A*. *thaliana* Col0 and amino acid varaitions of GATA TFs of 19 *A*. *thaliana*. (A) is the phylogenetic analysis of *A*. *thaliana* Col0 GATA domains. This is made of a neighbor-joining tree of GATA domain amino acid sequences from *A*. *thaliana* Col0 GATA TFs. Bootstrap values calculated from 10,000 replicates are shown on the tree except that those values are lower than 50. The scale bar corresponds to 0.10 estimated amino acid substitutions per site. (B) is protein domain organization of the corresponding GATA TFs. Black boxes with four different patterns indicate GATA domains with four different types. Type IV_b_, IV_c_, IV_4_, and IV_p_ mean CX_2_CX_18_CX_2_C, CX_2_CX_20_CX_2_C, CX_4_CX_18_CX_2_C, and partial forms, respectively. Yellow- and orange-colored boxes indicate functional domains of TIFY and CCT, respectively. Subfamily names were displayed at the right side. Definitions of each box were presented in the right-top side. (C) shows GATA domain sequence types along with each GATA TF and *A*. *thaliana* genome. The X-axis of the matrix presents ecotypes of *A*. *thaliana* and Y-axis means each GATA TFs. Four different colors, white, yellow, orange, and green, indicate different amino acids in each *Arabidopsis* GATA TFs and the blue color presents heterogeneous amino acid in a specific position caused by heterogeneous nucleotide. Dark grey color means missed GATA TFs along with 19 ecotypes.

*A*. *thaliana* GATA genes belonging to subfamilies I, II, and IV contain a single GATA domain with CX_2_CX_18_CX_2_C form (Type IV_b_); while GATA genes in subfamily III exhibit a single GATA domain with CX_2_CX_20_CX_2_C form (Type IV_c_; Fig 4) [73,75]. Except two GATA domain types, we identified additional domain types: CX_4_CX_18_CX_2_C type which contains four amino acids in the first cysteine-cysteine is designated as type IV_4_ [75]. Type IV_4_ (CX_4_CX_18_CX_2_C) is considered as an unusual pattern of the GATA domain because of four amino acids in the first two cysteines which have a role in binding zinc molecule. Based on the previous study which tested the ability of DNA binding with zero to five amino acids between two cysteines in C2H2 zinc finger TFs of which three-dimensional structure is almost similar to that of GATA TFs except for two histidines binding to zinc ion and shorter length of the linkers between two cysteines and two histidines [117]. It is similar to the conventional GATA domain as well as is found in many GATA genes: AtGATA29 in *A*. *thaliana*, 28035.m000366 gene in *Ricinus communis* [84], GmGATA50 gene in *G*. *max* [80], and eight GATA genes from *Populus* species (PdGATA20, PeGATA19, PeGATA20, PeGATA23, PpGATA21, PpGATA22, PtaaGATA20, and PtrGATA10; Kim et al., under revision). CX_15_CX_2_C type designed as type IV_p_ is a partial GATA domain identified in AtGATA26a. The partial GATA domain in AtGATA26a was caused by alternative splicing forms so that AtGATA26b and AtGATA26c have intact GATA domain. In addition, AtGATA26a without additional known functional domain was expressed in leaves of cold assimilated *A*. *thaliana* [118]. Moreover, the third GATA domain of the OsGATA24 gene in *O*. *sativa* covers partial GATA domain only with two latter cysteines [73] and MdGATA27 gene (CX_14_CX_2_C) and MdGATA35 gene (CX_21_CX_2_C) in *Malus domestica* [82] present three cysteines, the same form of AtGATA26a (Table 5). *P*. *edulis* genome presents five GATA genes of which domain is partial type (Table 5), which is the largest number among 12 species (Table 5). Taken together, type IV_p_ can be defined as [CX_2-4_C]X_12-21_[CX_2_C], indicating that one of the amino acid patterns inside brackets can be omitted, and it may retain DNA-binding function.

All subfamily III GATA TFs from 19 *A*. *thaliana* genomes contain two additional domains (Fig 4B): one is CCT domain (IPR010402) found in CONSTANS in *A*. *thaliana* [119] which is involved in circadian clock and flowering control, and the other is TIFY domain (IPR010399) which mediates homo- and heteromeric interactions between TIFY proteins and other specific TFs [120,121]. In contrast, some of GATA TFs in subfamily III from other plant species do not contain CCT and/or TIFY domains: 13 GATA TFs from six *Populus* species (Kim et al., in preparation) and 29838.m001723 gene in *R*. *communis* [84]. Some of *Populus* GATA TFs (Kim et al., in preparation) and OsGATA19b in *O*. *sativa* [101] lost CCT and/or TIFY domains by alternative splicing events. There are no GATA TFs without CCT and/or TIFY domains in 19 *A*. *thaliana* genomes, suggesting that two subfamilies from subfamily III, named as subfamilies IIIa and IIIb, can be defined as GATA TFs with or without CCT and/or TIFY domains, respectively.

### Comparison of GATA domain sequences from 19 *A*. *thaliana* genomes

**Fig 5:**
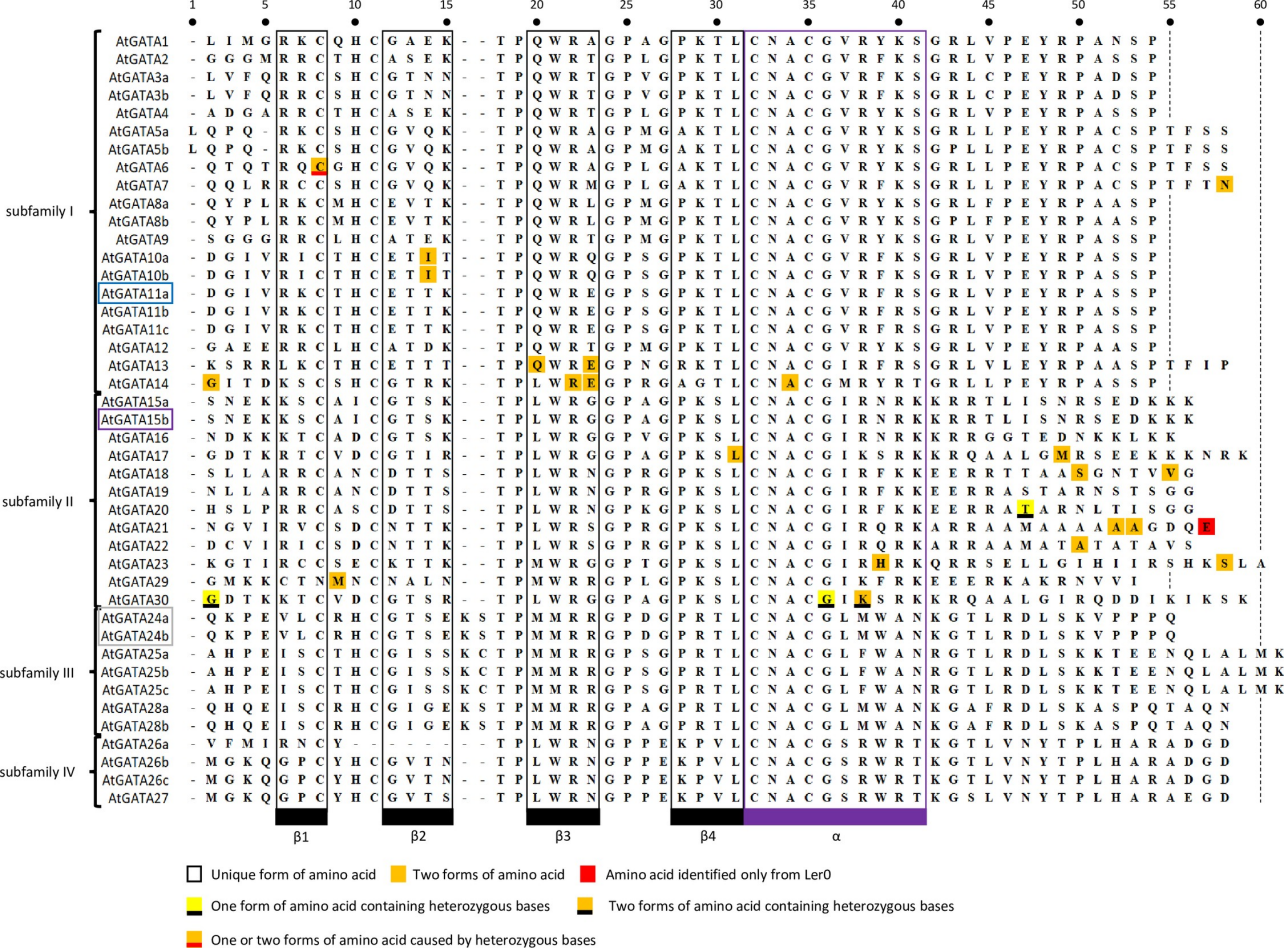
Amino acid patterns of GATA domain from 19 *A*. *thaliana* genomes. It shows amino acid patterns of GATA domains of GATA TFs from 19 *A*. *thaliana* genomes. Purple colored GATA gene name indicates GATA TFs found only in Kn0 genome and grey colored GATA gene names mean that some *A*. *thaliana* genomes do not have GATA gene. Blue colored GATA gene name presents uniquely found in *A*. *thaliana* Col0 genome. Colors on aligned amino acids of the GATA domain indicate the number of amino acids in that position. Black and purple boxes under the alignment indicate the position of beta-sheet and alpha helixes, respectively. Black and purple border boxes indicate an area of the beta sheet and alpha helix areas.

Among distinct 43 *A*. *thaliana* GATA TFs, GATA domain sequences of 30 GATA TFs are identical including two cases, i) AtGATA15b uniquely identified in *A*. *thaliana* Kn0 genome and AtGATA11a only from *A*. *thaliana* Col0 and ii) AtGATA24a and AtGATA24b missed in *A*. *thaliana* Hi0, Ler0, Mt0, and Ws0 genomes (Fig 4C). Thirteen out of 43 distinct GATA TFs (30.23%) have multiple forms of GATA domain sequences. The AtGATA14 gene has four forms among 19 *A*. *thaliana* genomes, which is the largest number among the 13 GATA TFs (Fig 4C). AtGATA13, AtGATA17, and AtGATA18 genes have three forms and the rest nine GATA TFs contain two forms of GATA domains in 19 *A*. *thaliana* genomes (Fig 4C). Among nine GATA TFs with two GATA domain forms, the AtGATA6 gene presents one heterozygous amino acid in *A*. *thaliana* Mt0 genome because one nucleotide inside the AtGATA6 gene is a heterozygous base (K = G or T; Fig 4C and Table 6), causing critical amino acid changes from cysteine (C) to glycine (G) in the first conserved cysteine of GATA domain (Fig 5). It indicates that *A*. *thaliana* Mt0 may have two duplicated AtGATA6 genes with mutation or AtGATA6 on Mt0 genome is heteroallele. In addition, five heteroallele cases identified in AtGATA17, AtGATA20, and AtGATA30 are also identified without changing amino acids (Table 6). Moreover, all 11 GATA TFs in subfamilies III and IV are identical, presenting low diversity among 43 GATA TFs. Different diversity of GATA domain sequences in four subfamilies indicates different evolutionary speed.

**Table 6:** List of variable amino acids found in GATA domains of *A*. *thaliana* GATA TFs.

Gene name	Position (aa)	Amino acid (Codon)	Ecotypes
AtGATA6	8	C or G (KGT)	Mt0
		C	other
AtGATA7	58	T	Ws0, Bur0
		N	other
AtGATA10a	14	T	Bur0, Rsch4, Wu0
AtGATA10b		I	other
AtGATA13	20	L	Zu0
		Q	other
	23	K	Wil2
		E	other
AtGATA14	2	C	Oy0, Edi0, Po0
		G	other
	22	K	No0
		R	other
	23	V	Rsch4, Sf2, No0, Wu0, Ler0, Mt0
		E	other
	34	V	Tsu0
		A	other
AtGATA17	31	F (TTY)	Sf2
		L	other
	49	V	Bur0, Sf2, Tsu0, Zu0, Mt0
		M	other
AtGATA18	50	T	Sf2, Col0
		S	other
	55	I	Bur0, Tsu0
		V	other
AtGATA20	47	T (ACY)	Hi0
		T	other
AtGATA21	52	-	Ler0
		A	other
	53	-	Ler0
		A	other
	57	E	Ler0
		-	other
AtGATA22	50	P	Can0
		A	other
AtGATA23	39	Y	Wil2, Can0
		H	other
	58	G	Wil2, Can0
		S	other
AtGATA29	9	I	Bur0, Ct1
		M	other
AtGATA30	2	G (GGM)	Mt0
		G	other
	36	G (GGM)	Po0
		G	other
	38	N (AAY)	Po0
		K	other

Two out of 19 *A*. *thaliana* genomes, *A*. *thaliana* Rsch4 and Wu0, present identical patterns of GATA domain sequences of 41 GATA TFs, while those of the other *A*. *thaliana* genomes are different from each other (Fig 4C). All 39 GATA TFs in the *A*. *thaliana* Hi0 genome present abundant GATA domain patterns among 19 *A*. *thaliana* genomes; while *A*. *thaliana* Col0, Edi0, Ct1, Can0, Kn0, No0, Oy0, and Ws0 genomes contain one minor domain sequence (Fig 4C). Here, not all GATA TFs of the *A*. *thaliana* Col0 genome are abundant patterns, suggesting that the virtual genome of *A*. *thaliana* which contains all types of *A*. *thaliana* GATA genes should be constructed for understanding intra-species features of GATA genes in *A*. *thaliana*.

In detail, 22 out of 2,195 amino acids (1.002%) originated from GATA domain sequences of 41 GATA TFs except for AtGATA11a and AtGATA15b have variations across the 19 *A*. *thaliana* genomes (Fig 5). Five amino acids of GATA domains originated from heterozygous bases are not changed in contrast to the heterozygous bases found in the AtGATA6 gene: three amino acids in the AtGATA30 gene (*A*. *thaliana* Po0 and Mt0) and one amino acid in AtGATA17 (*A*. *thaliana* Sf2) and AtGATA20 gene (*A*. *thaliana* Hi0). These six amino acids from heterozygous bases suggest additional analyses of at least *A*. *thaliana* Mt0, Po0, Hi0, and Sf2 genomes to probe the reason why they have heterozygous bases in GATA genes.

Amino acid variations of GATA domain sequences within 19 *A*. *thaliana* genomes are not so high; most of the amino acids are conserved (Fig 5). It is reasonable because the GATA domain is critical to recognize specific DNA sequences (WGATAR) [73,74]. The number of heterozygous amino acids among 19 ecotypes identified in alpha helix and four beta sheets (Fig 5) of GATA TF and the number of those amino acids outside alpha helix and beta sheet structure is exactly the same, as 11. Maximally two amino acids are found in a certain position of the GATA domain (Fig 5). One amino acid, glutamine (E), in the end of the GATA domain of the AtGATA21 gene is only found in *A*. *thaliana* Ler0 genome caused by missing two alanines (A) near to the end of the domain (a red color amino acid in Fig 5). However, we confirmed that glutamine after GATA domain were found in other *A*. *thaliana* genomes indicating that the GATA domain of AtGATA21 from the Ler0 genome should not include this glutamine. All GATA genes having alternative splicing forms do not present any amino acid changes in the GATA domain except the AtGATA10 gene. AtGATA10 genes originated from three genomes, *A*. *thaliana* Bur0, Rsch4, and Au0, show threonine (T) instead of isoleucine (I) in the second beta sheet (Fig 5). Except for AtGATA11a and AtGATA15b, subfamily I contains 10 heterozygous amino acids among 19 *A*. *thaliana* genomes, while subfamily II has 11 heterozygous amino acids. It shows that the frequency of heterozygous amino acids in subfamily II (1.86%) is larger than that of subfamily I (1.01%), presenting high diversity of heterozygous amino acids in the GATA domain in subfamily II. There is no heterozygous amino acid in both subfamilies III and IV. These results indicate different evolutionary histories of the GATA domain in each subfamily.

Amino acids in a specific position of the GATA domain were grouped based on properties of amino acids: Inside alpha helix and beta sheets, two out of eleven amino acid changes (18.18%) present the same group of amino acid which may not affect the three-dimensional structure of GATA domain (Fig 5). It is interesting that amino acid changes found in 19 *A*. *thaliana* genomes may affect the three-dimensional structure of the GATA domain. While five out of eleven amino acid changes found outside of alpha helix and beta sheets show the same properties of amino acids, which can be explained that these areas are not important to form the three-dimensional structure of the GATA domain so that amino acid changes can change their properties easily.

In detail, three amino acid changes are in the alpha helix structure, while eight amino acid changes were identified inside four beta sheets (Fig 5 and Table 6). Two out of the three heterogeneous amino acids in alpha helix display lysine (K) or asparagine (N) identified in AtGATA30 and histidine (H) or tyrosine (Y) found in AtGATA23, changing a property of amino acids (Fig 5 and Table 6). Especially for the case of lysine or asparagine, the helical penalty increased from 0.26 kcal/mol to 0.66 kcal/mol [122], potentially disturbing the formation of alpha helix structure. Five out of eight amino acid changes were located in the boundary of beta sheets, which may be tolerable for allowing different properties of amino acids because they are directly linked to linker amino acids of which lengths are relatively short (2 to 4 amino acids). There are three out of eight amino acid changes inside the beta sheet structure of GATA domains: one is arginine (R) at the third amino acid of the third beta sheet at AtGATA14 gene containing amino acid change to lysine (K). Both arginine and lysine have the same characteristics having electrically charged side chains in their residue. The rest two are isoleucine (I) at third amino acids in the second beta sheet of AtGATA10a and AtGATA10b covering threonine (T) change. Threonine has polar uncharged residue, while isoleucine has a hydrophobic side chain. Because the three-dimensional structure of beta sheets faces with another beta sheet, differences of proletaries of threonine and isoleucine may not affect their three-dimensional structure severely. Taken together, amino acid changes in the GATA domain will not affect severely their basic three-dimensional structure, presenting that amino acid changes found in 19 *A*. *thaliana* genomes do not affect the DNA-binding function of GATA TFs, however there is a possibility for these variations to affect DNA binding affinity subtlely, which can affect regulatory gene networks supported by the previous studies [123,124].

### Characterized biological functions of GATA TFs in Col0 and their distribution among 19 *A*. *thaliana* genomes

15 out of 30 *A*. *thaliana* Col0 GATA genes have been studied about their biological functions (Table 7). Five GATA genes belong to subfamily I and seven are from subfamily II and the remaining three GATA genes are in subfamily III. AtGATA1, AtGATA2, AtGATA3, and AtGATA4 (subfamily I) genes may be involved in the regulation of some of the light-responsive genes [125]. AtGATA8 (BME3; subfamily I) gene is a positive regulator of *Arabidopsis* seed germination [76]. AtGATA18 (HAN; subfamily II) gene is required to position the proembryo boundary in the early *Arabidopsis* embryo [77] and AtGATA21 (GNC) and AtGATA22 (GNL/CGA1) genes in subfamily II regulate chloroplast development, growth, and division [126,127]. In addition, AtGATA15, AtGATA16, AtGATA17, and AtGATA30 play roles of cytokinin-regulated development [128]. Interestingly, only these five GATA genes belonging to Subfamily II have amino acid variations across 19 *A*. *thaliana* genomes also supported by one of the results of this study that subfamily II presents the largest number of amino acid variations (Fig 4). It also implies subtle variations of their biological functions, e.g. different DNA binding sequences. AtGATA24 (ZML1) and AtGATA28 (ZML2) genes in subfamily III mediate cryptochrome1-dependent response [102] and AtGATA25 (ZIM; subfamily III) gene is involved in hypocotyl and petiole elongation [78].

**Table 7:** Characterized GATA genes in *A*. *thaliana* Col0.

GATA name	Involved Functions	Sub-family	Reference
AtGATA1 (GATA-1)	Regulation of light-responsive genes	I	[125]
AtGATA2 (GATA-2)			
AtGATA3 (GATA-3)			
AtGATA4 (GATA-4)			
AtGATA8 (BME3)	Regulation of seed germination	I	[76]
AtGATA15 (GATA15)	Cytokinin-regulated development, including greening, hypocotyl elongation, phyllotaxy, floral organ initiation, accessory meristem formation, flowering time, and senescence	II	[128]
AtGATA16 (GATA16)			
AtGATA17 (GATA17)			
AtGATA30 (GATA17L)			
AtGATA18 (HAN)	Regulation of shoot apical meristem and flower development	II	[77,129–131]
	Stable establishment of cotyledon identity during embryogenesis		[131]
	Position the proembryo boundary in the early *Arabidopsis* embryo		[77]
AtGATA21 (GNC)	a nitrate-inducible member important for chlorophyll synthesis and glucose sensitivity	II	[126]
	Modulation of chlorophyll biosynthesis (greening) and glutamate synthase (GLU1/Fd-GOGAT) expression		[132,133]
	Downstream effectors of floral homeotic gene action by controlling two MADS-box TFs		[134]
	Control of convergence of auxin and gibberellin signaling		[135,136]
	Control of greening, cold tolerance, and flowering time		[137]
	Regulation of chloroplast development, growth, and division as well as photosynthetic activities		[127,138]
	Cytokinin-regulated development, including greening, hypocotyl elongation, phyllotaxy, floral organ initiation, accessory meristem formation, flowering time, and senescence		[128]
	PIF- and light-regulated stomata formation in hypocotyls		[139]
AtGATA22 (GNL/CGA1)	Response of blue light and cytokinin	II	[140]
	Modulation of chlorophyll biosynthesis (greening) and glutamate synthase (GLU1/Fd-GOGAT) expression		[132,133]
	Downstream effectors of floral homeotic gene action by controlling two MADS-box TFs		[134]
	Control of convergence of auxin and gibberellin signaling		[135]
	Control of greening, cold tolerance, and flowering time		[137]
	Regulation of chloroplast development, growth, and division as well as photosynthetic activities		[127,138]
	Cytokinin-regulated development, including greening, hypocotyl elongation, phyllotaxy, floral organ initiation, accessory meristem formation, flowering time, and senescence		[128]
	PIF- and light-regulated stomata formation in hypocotyls		[139]
AtGATA24 (ZML1)	Mediation of cryptochrome1-dependent response	III	[102]
AtGATA28 (ZML2)			
AtGATA25	Hypocotyl and petiole elongation	III	[78]

Fourteen out of 15 characterized GATA genes were also found in the other 18 *A*. *thaliana* genomes, indicating that biological functions of GATA genes in *A*. *thalian*a may be conserved and essential to their life cycle. However, one GATA gene, AtGATA24, is missed in the gene model of *A*. *thaliana* Hi0, Ler0, Mt0, and Ws0 genomes. Based on characterized functions of AtGATA24 (ZIM1) and AtGATA28 (ZIM2) genes, two GATA genes may present redundant or co-operational manners, which can explain the missed phenomenon on four *A*. *thaliana* genomes. However, it requires additional experimental researches to probe this hypothesis: e.g., both GATA genes contain CCT domains, related to protein-protein interactions [141], inferring that in the case that AtGATA24 and AtGATA28 genes form hetero-dimers, both genes are essential for elongating petiole and hypocotyl cells. Another possibility to explain this phenomenon is that gene models of four *A*. *thaliana* genomes may missed this gene in some reason; however, it may not be occurred easily because the same gene prediction program to predict genes of the eighteen *A*. *thaliana* genomes was used [52]. In addition, *A*. *lyrata* (EFH59549.1) and *A*. *helleri* (Araha.17146s0001.1), which are neighbor species of *A*. *thaliana*, also have AtGATA24 gene, indicating that functional redundant of AtGATA24 and AtGATA28 genes should be probed in the near future.

### Chromosomal distribution of GATA genes of 19 *A*. *thaliana* genomes

**Fig 6:**
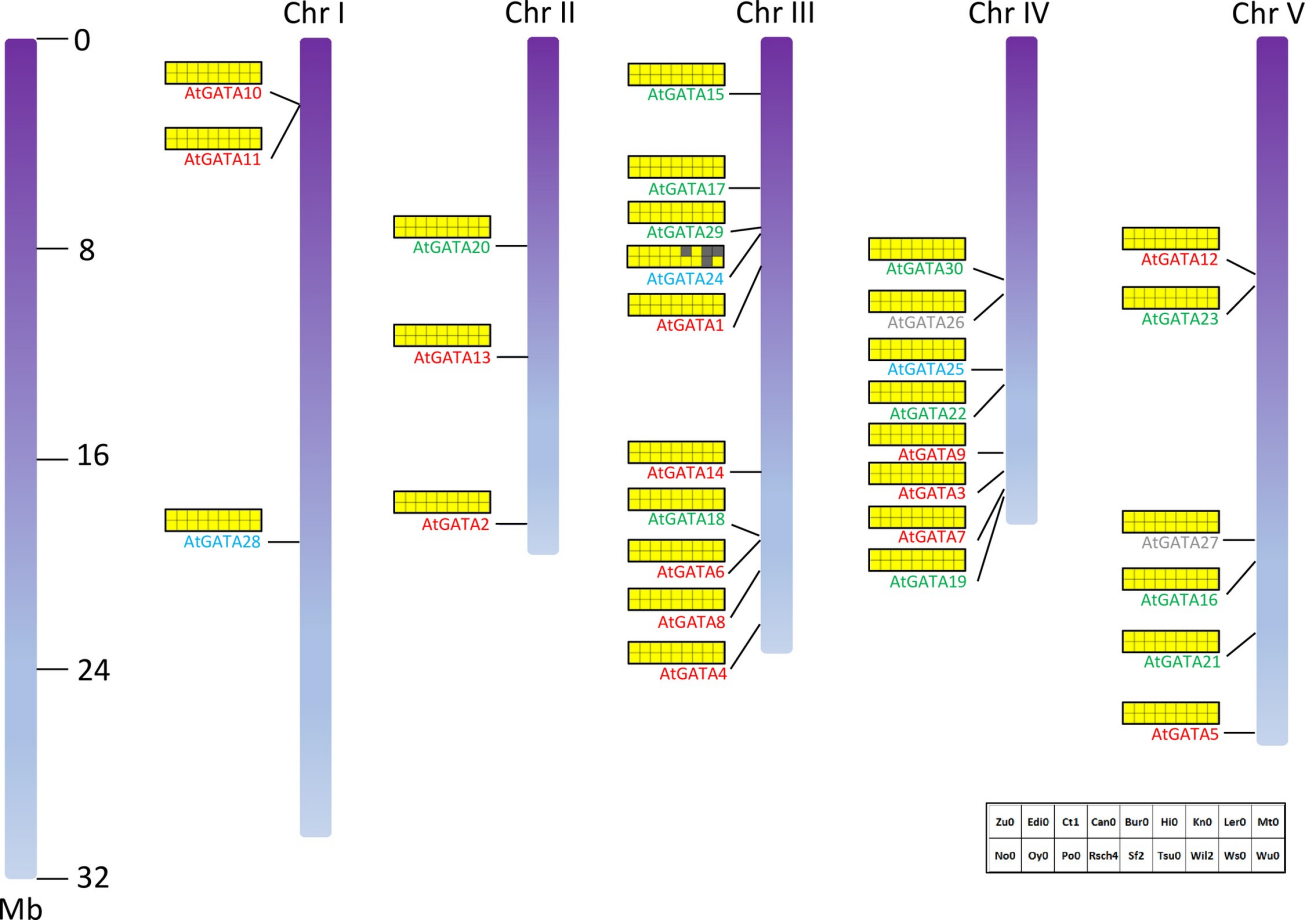
Chromosomal distribution of *A*. *thaliana* GATA genes among 19 genomes. Gradient purple bars indicate the chromosome of *A*. *thaliana* Col0. The left bar indicates the length of the chromosome. Red, green, sky blue, and gray GATA gene names mean subfamilies I, II, III, and IV, respectively. An array of small squares beside chromosomes presents the existence of GATA genes among 18 *A*. *thaliana* genomes: yellow color means existence and white color is non-existence case.

Several characteristics have been confirmed by the chromosome distribution of GATA genes in nineteen *A*. *thaliana* genomes (Fig 6). Chromosomes I and II contain only three GATA genes; while chromosomes III, IV, and V cover 10, 8, and 6 GATA genes, respectively. One exception is the AtGATA24 gene on chromosome III, missed in *A*. *thaliana* Hi0, Ler0, Mt0, and Ws0 genomes. Based on the density of GATA genes on chromosomes, chromosomes III and IV present similar density (chromosome III is 2.35 Mb/gene and chromosome IV is 2.32 Mb/gene); while chromosome I displays 10.14 Mb/gene, the lowest density.

GATA genes in subfamily I are distributed in all five chromosomes and those of subfamily II are in chromosomes II to V. GATA genes belonging to subfamilies III and IV, containing a small number of GATA genes, are distributed in chromosomes I, III, and IV, and IV and V, respectively. Biased distribution of GATA genes along with chromosomes is also found in *G*. *max* [80] and *Solanum lycopersicum* [85].

Four pairs of GATA genes can be grouped because the distance between two GATA genes is less than 170 kb: AtGATA10 and AtGATA11 genes (distance is only 1,638 bp), which can be a candidate for gene duplication, AtGATA6 and AtGATA18 genes (distance is 61 kb), AtGATA7 and AtGATA19 genes (distance is 120 kb), and AtGATA24 and AtGATA29 genes (distance is 167 kb). Interestingly, except AtGATA10 and AtGATA11 genes, members of three pairs are belonging to different subfamilies, reflecting that three pairs of GATA genes are nearly located coincidentally.

### Principle component analysis of *Arabidopsis* GATA genes

**Fig 7:**
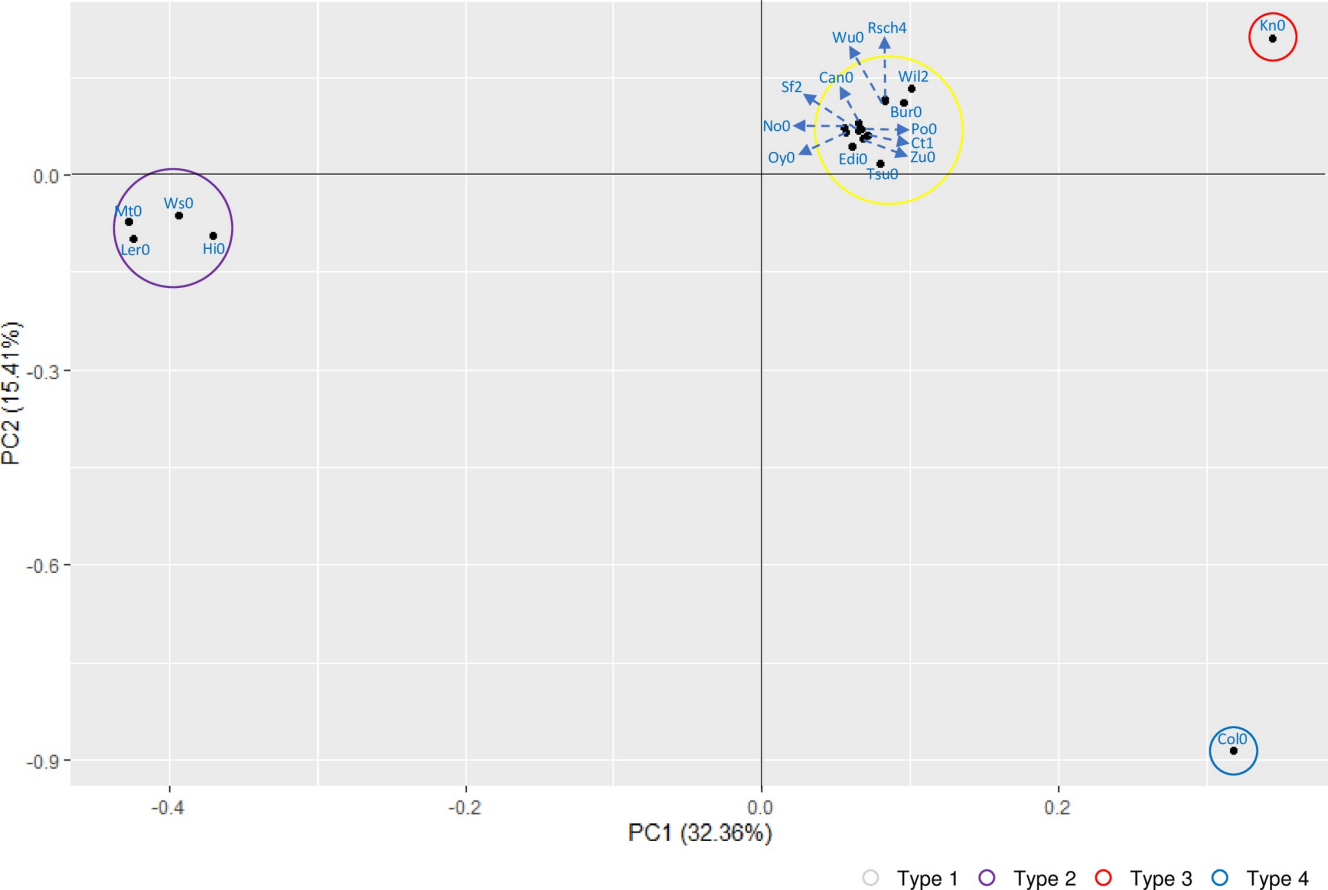
Principal components analysis result of 28 characteristics of GATA genes identified from 19 *Arabidopsis* ecotypes. It shows the two-dimensional model of 19 *Arabidopsis* ecotypes derived from principal components analysis of 28 characteristics of GATA genes identified from 19 *Arabidopsis* ecotypes. Gray, purple, blue, and red circles are corresponding to Type 1, 2, 3, and 4 mentioned in Table 4, respectively. The ecotype name colored blue represents the specific dot.

To understand the relationship of 19 *A*. *thaliana* ecotypes based on the GATA genes identified in this study, we extracted 28 characteristics from properties of the whole genome, number of GATA genes, GATA subfamily, number of alternative splicing forms of GATA genes, and amino acid changes and conducted principal component analysis (PCA) using the R package (see Materials and Methods). The result of PCA displays four distinct groups clearly (Fig 7), which is corresponding to four types defined in Table 4. In detail, Col0 (blue circle in Fig 7) and Kn0 (red circle in Fig 7) are completely separated, caused by one additional GATA TFs, AtGATA11a and AtGATA15b, respectively. It indicates that the power of characteristics related to the number of GATA genes can be dominant to be classified them into four groups (Fig 7). Once additional studies investigating intraspecific variations of GATA genes using plant genomes are available, we can know whether this trend is general across the plant species or not.

### Phylogenetic relationship of *Arabidopsis* GATA genes among 19 ecotypes

Based on nine common *Arabidopsis* GATA genes across 19 ecotypes as well as those of *A*. *lyrata*, we constructed bootstrapped phylogenetic trees of maximum-likelihood (ML), neighbor-joining (NJ), and Bayesian inference (BI) based on the concatenated alignment of the nine common GATA genes (Fig 7B). In addition, we also assembled the complete chloroplast genome of 15 ecotypes excluding Col0, Ler0, and Tsu0 because of available complete chloroplast genomes [142–144] as well as Sf0 due to lack of NGS raw reads in NCBI. In total, eighteen complete *Arabidopsis* chloroplast genomes together with that of *A*. *lyrata* were utilized for constructing the phylogenetic trees (Fig 7A).

**Fig 8:**
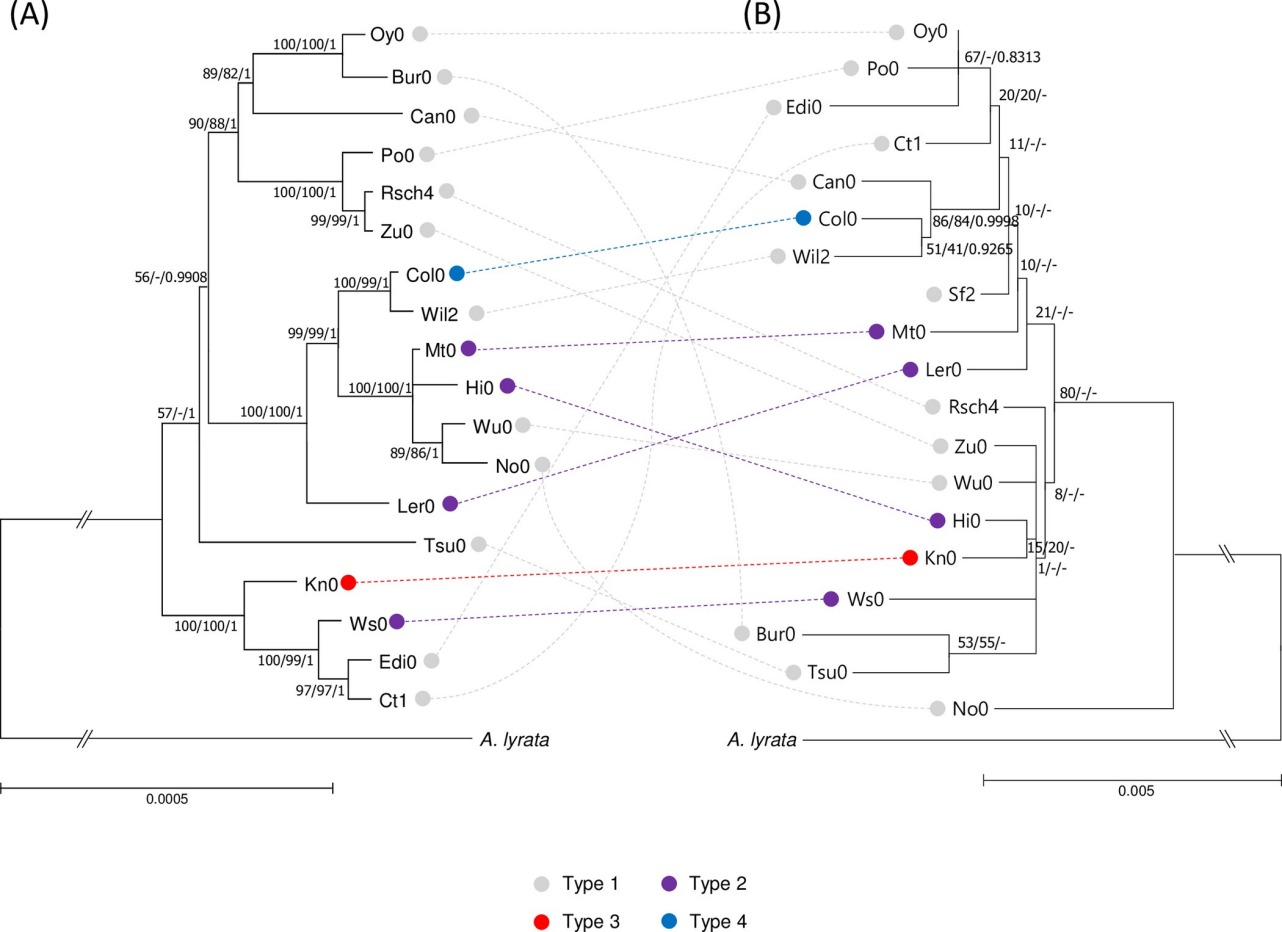
Phylogenetic relationship of GATA genes and chloroplast genomes of *Arabidopsis* ecotypes. (A) is a bootstrapped maximum-likelihood phylogenetic tree of 18 *A*. *thaliana* and *A*. *lyrata* chloroplast genomes. (B) presents a bootstrapped maximum-likelihood phylogenetic tree of concatenated common GATA genes across 19 *A*. *thaliana* ecotypes and *A*. *lyrata*. Numbers on branches in both phylogenetic trees indicate supporting values of maximum-likelihood, neighbor-joining, and Bayesian inference tree, respectively. The scale bars of both trees indicate estimated DNA substitutions per site. Gray, purple, blue, and red circles are corresponding to Types 1, 2, 3, and 4 mentioned in Fig 8 and Table 4, respectively. The dotted straight and curved lines connect the same ecotype in both trees.

Interestingly, both trees show almost completely incongruent except the terminal clade containing Col0 and Wil2, which forms one clade with high supportive values in chloroplast genome tree (Fig 7A) and with high supportive value of BI tree in the GATA gene tree (Fig 7B). Supportive values of the chloroplast tree present a high in most clades (Fig 8A); while those of the GATA gene tree do not, indicating that concatenated common GATA gene sequences are not enough to solve phylogenetic relationships of 19 ecotypes of *A*. *thaliana* (Fig 8B). In addition, the four types which are defined based on the number of GATA genes (Table 4) and are the same as the groups identified in PCA (Fig 7) were mapped on both phylogenetic trees (Fig 8). It displays no clear relationship between these types and clades (Fig 8), indicating that the presents and absences of GATA TFs are not related to evolutionary history.

To find the relationship among the geographical distribution of *Arabidopsis* ecotypes and phylogenetic relationships of *Arabidopsis* chloroplast genomes and their GATA genes, we selected countries which contain more than one ecotype: four ecotypes (Ler0, No0, Po0, and Wu0) derived from Germany, three ecotypes (Rsch4, Wil2, Ws0) from Russia, and two ecotypes (Can0 and Sf2) derived from Sapin (S2 Fig). Ler0, No0, Po0, and Wu0 from Germany are not clustered in the phylogenetic tree of GATA genes (Fig 8B). No0 and Wu0 ecotypes were clustered only in the chloroplast phylogenetic tree (Fig 8A); while all four German ecotypes were not clustered in the GATA gene tree (Fig 8B). Three and two ecotypes from Russia and Spain, respectively, were not clustered in both three (Fig 8). It indicates that there is no clear relationship among the geographical distribution of *Arabidopsis* ecotypes and phylogenetic relationships of *Arabidopsis* chloroplast genomes and their GATA genes.

## Conclusion

Till now, there have been no intra-species genome-wide comparative analyses in the plant GATA gene family. We conducted comparative analyses using 19 *A*. *thaliana* genomes to unravel the characteristics of the GATA gene family: Only subfamily III presents differences number of GATA genes among 19 *A*. *thaliana* genomes; while alternative splicing forms of GATA genes in both subfamilies II and III present differences at the genome level. 13 out of 41 *A*. *thaliana* GATA TFs except two unique GATA TFs, AtGATA11a and AtGATA15b present different amino acids along with other 18 *A*. *thaliana* genomes and, interestingly, half of these variable amino acids are found in structural elements, including alpha helix and beta sheets. AtGATA24 (ZIM1) gene is missed in four *A*. *thaliana* genomes, *A*. *thaliana* Hi0, Ler0, Mt0, and Ws0, requiring additional experiments to show whether that gene is replaceable to AtGATA28 (ZIM2) gene or not. Moreover, the differences of an average number of alternative splicing forms of GATA genes along with subfamilies may represent subfamily-specific functional diversity. PCA result presents the four groups clearly (Fig 7), which is the same as the four types defined based on the number of GATA genes (Table 4). To understand phylogenetic relationships of *Arabidopsis* GATA genes and chloroplast genomes, we constructed bootstrapped phylogenetic trees, showing mostly incongruent. Moreover, there is no clear relationship between geographical distribution and their phylogenetic relationships of chloroplast genomes and GATA genes. Taken together, we successfully identified the genome-wide intraspecific variations of GATA TFs among 19 ecotypes and they are evolutionarily neutral, which can be explained by the fact that GATA TFs have essential regulatory roles for survival, such as seed germination [76] and hypocotyl elongation [128].

To date, more than 1,700 *A*. *thaliana* genomes are available [5,18–20,50–53] and more than 4,000 *O*. *sativa* genomes [26,29,54–61] are available, but their sequences were not processed as independent genome sequence: only raw sequences and/or sequence variations including single nucleotide polymorphisms and insertions and deletions are available. Once these genome sequences can be applied for this genome-wide identification method of GATA TFs, they will provide high-resolution intraspecific variations of the GATA gene family, which will provide insights into the evolution of GATA TFs within species with comparing with various researches especially for investigating intraspecific variations of their organelle genomes of diverse plant species [145–184]. In addition, these intraspecific variations of GATA TFs may provide the molecular mechanisms of intraspecific phenotypic variations in the aspect of the gene regulation network. One genome-wide association study using *B*. *napa* identified deletion region on the genome which contains one TF, orthologs to the HAG1 (At5g61420) controlling aliphatic glucosinolate biosynthesis in *A*. *thaliana* [123]. Another example is chickpea bZIP TF which can control its height based on QTL analysis [124]. It indicates that the existence or absence of TFs among cultivars or individuals of the sample species as well as their intraspecific amino acid variations can explain and predict intraspecific variations of phenotypes. We expect that our approach will contribute to understanding the intraspecific characteristics of the GATA gene family in detail as well as provide additional evidence of their biological roles including variable practical phenotypes inside the species.

## Supporting information

S1 Fig BLAST results of AtGATA24 homologs in *A*. *lyrata* and *A*. *halleri*. (A) displays AtGATA24 homologs of *A*. *lyrata*. (B) shows AtGATA24 homologs of *A*. *halleri*.
(PPTX)

S2 Fig The geographical location of 19 *A*. *thaliana* genomes. The red circle means the geographical location of the species. The red circle containing a yellow star implies a not-precise location due to the lack of GPS coordination in Russia.
(PPTX)

S1 Table List of SRA raw reads of 17 *A*. *thaliana* ecotypes deposited in NCBI, which were used for assembling complete chloroplast genomes. (DOCX)

S2 Table List of identified 773 GATA TFs from 19 *A*. *thaliana* genomes. (DOCX)
