# Supplementary figures and images for: Genome-wide comparative analyses of GATA transcription factors among 19 Arabidopsis ecotype genomes: Intraspecific characteristics of GATA transcription factors
Source: PLoS One. 2021 May 26;16(5):e0252181. doi: 10.1371/journal.pone.0252181 (PMC8153473; doi:10.1371/journal.pone.0252181)

## Slide 1
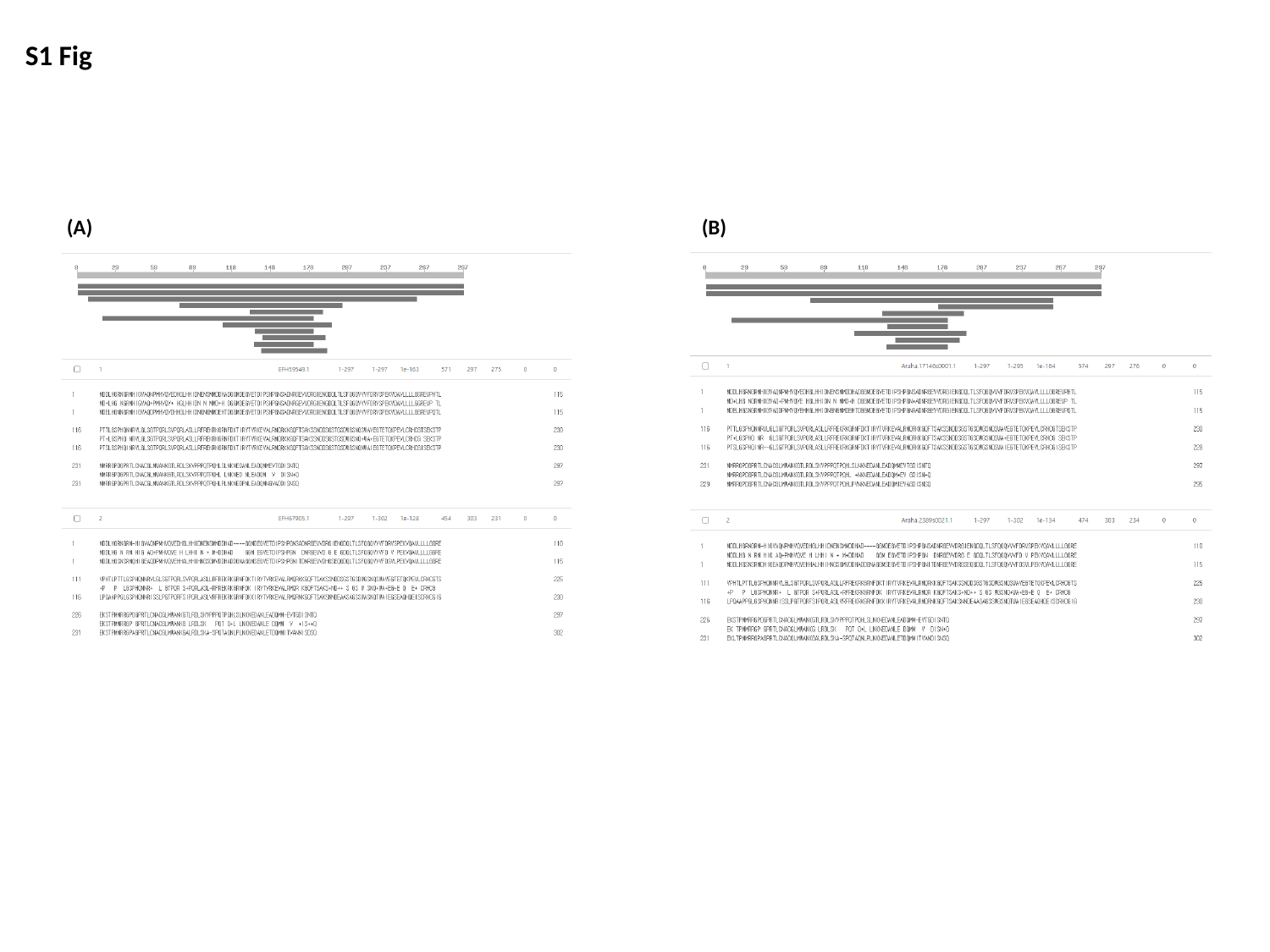

S1 Fig
(A)
(B)

Supplement: S1 Fig — (A) displays AtGATA24 homologs of A. lyrata. (B) shows AtGATA24 homologs of A. halleri. (PPTX) [file pone.0252181.s001.pptx]
