# Supplementary material for: Genome-wide comparative analyses of GATA transcription factors among 19 Arabidopsis ecotype genomes: Intraspecific characteristics of GATA transcription factors
Source: PLoS One. 2021 May 26;16(5):e0252181. doi: 10.1371/journal.pone.0252181 (PMC8153473; doi:10.1371/journal.pone.0252181)
Supplement: S2 Fig — The red circle means the geographical location of the species. The red circle containing a yellow star implies a not-precise location due to the lack of GPS coordination in Russia. (PPTX) [file pone.0252181.s002.pptx]

## Slide 1
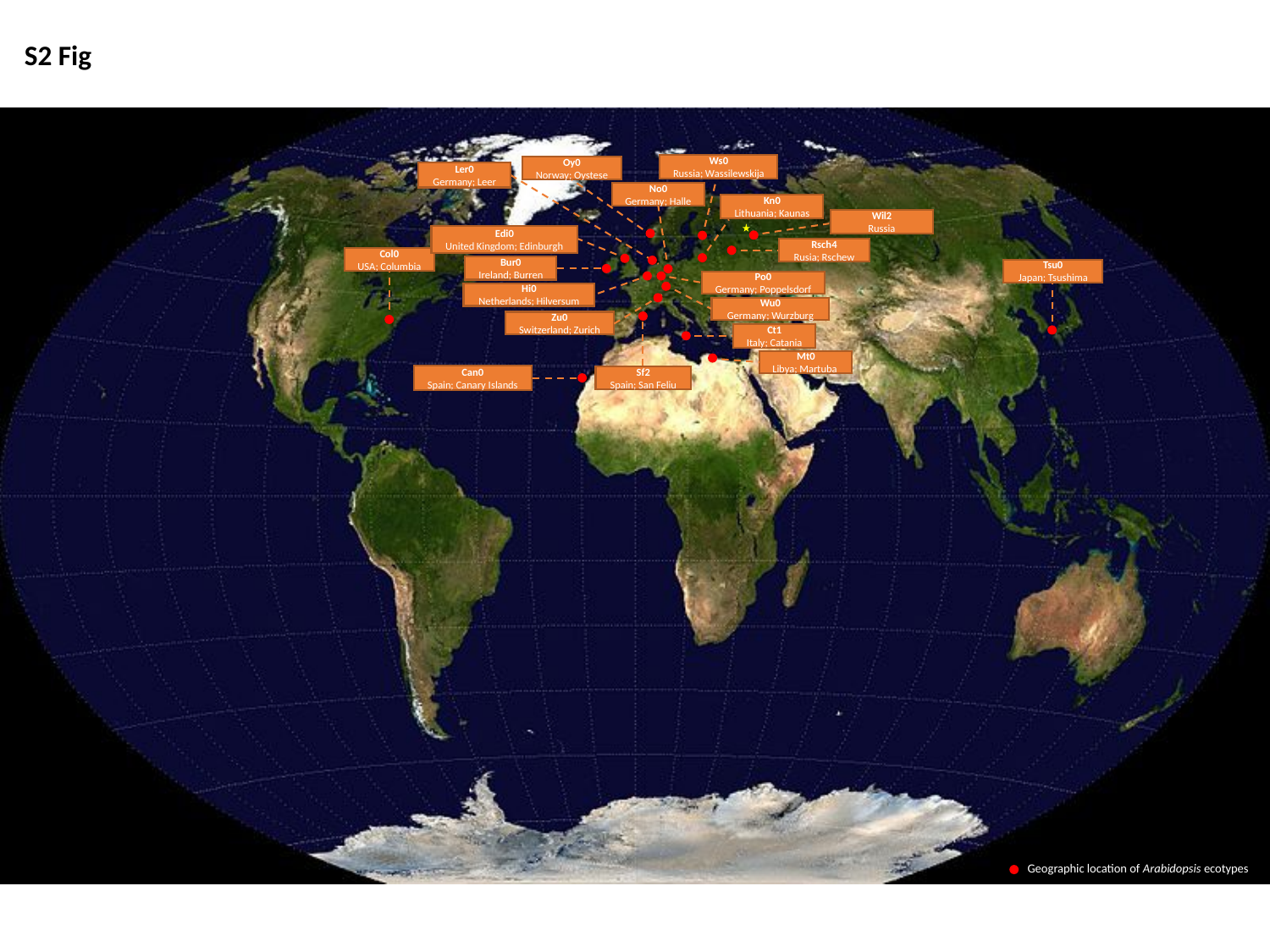

S2 Fig
Ws0
Russia; Wassilewskija
Oy0
Norway; Oystese
Ler0
Germany; Leer
No0
Germany; Halle
Kn0
Lithuania; Kaunas
Wil2
Russia
Edi0
United Kingdom; Edinburgh
Rsch4
Rusia; Rschew
Col0
USA; Columbia
Bur0
Ireland; Burren
Tsu0
Japan; Tsushima
Po0
Germany; Poppelsdorf
Hi0
Netherlands; Hilversum
Wu0
Germany; Wurzburg
Zu0
Switzerland; Zurich
Ct1
Italy; Catania
Mt0
Libya; Martuba
Can0
Spain; Canary Islands
Sf2
Spain; San Feliu
Geographic location of Arabidopsis ecotypes
