# Supplementary material for: Genome-wide comparative analyses of GATA transcription factors among 19 Arabidopsis ecotype genomes: Intraspecific characteristics of GATA transcription factors
Source: PLoS One. 2021 May 26;16(5):e0252181. doi: 10.1371/journal.pone.0252181 (PMC8153473; doi:10.1371/journal.pone.0252181)
Supplement: S1 Table — (DOCX) [file pone.0252181.s003.docx]

**S1 Table. List of NGS raw reads of 17 *A. thaliana* ecotypes deposited in NCBI, which were used for assembling complete chloroplast genomes**

| **Strain name** | **SRA accession** | **# of reads** | **Total size (bp)** |
| --- | --- | --- | --- |
| Edi0 | ERR031536 | 138,034,104 | 4,969,227,744 |
| Ct1 | ERR031534 | 143,912,778 | 5,180,860,008 |
| Can0 | ERR031532 | 129,819,980 | 4,673,519,280 |
| Bur0 | ERR031531 | 58,262,678 | 2,971,396,578 |
| Hi0 | ERR031540 | 112,859,224 | 4,062,932,064 |
| Kn0 | ERR031541 | 134,150,259 | 4,829,409,324 |
| Ler0 | ERR031543 | 142,904,774 | 5,144,571,864 |
| Mt0 | ERR031545 | 114,333,860 | 4,116,018,960 |
| No0 | ERR031547 | 144,501,621 | 5,202,058,356 |
| Oy0 | ERR031549 | 137,255,128 | 4,941,184,608 |
| Po0 | ERR031551 | 100,987,324 | 3,635,543,664 |
| Rsch4 | ERR031553 | 140,080,048 | 5,042,881,728 |
| Tsu0 | ERR031555 | 136,302,286 | 4,906,882,296 |
| Wil2 | ERR031557 | 95,017,296 | 3,420,622,656 |
| Ws0 | ERR031559 | 129,428,674 | 4,659,432,264 |
| Wu0 | ERR031562 | 87,120,680 | 3,136,344,480 |
| Zu0 | ERR031564 | 109,068,452 | 3,926,464,272 |
