# Supplementary material for: Genome-wide comparative analyses of GATA transcription factors among 19 Arabidopsis ecotype genomes: Intraspecific characteristics of GATA transcription factors
Source: PLoS One. 2021 May 26;16(5):e0252181. doi: 10.1371/journal.pone.0252181 (PMC8153473; doi:10.1371/journal.pone.0252181)
Supplement: S2 Table — (DOCX) [file pone.0252181.s004.docx]

**S2 Table. List of identified 773 GATA TFs from 19 *A. thaliana* genomes**

| **No** | **Ecotype** | **Sub-family** | **GATA name** | **Protein name** | **Length (aa)** | **# of exons** | **Chro- mosome** | **Genomic coordination** |
| --- | --- | --- | --- | --- | --- | --- | --- | --- |
| 1 | Col0 | I | AtGATA10b | rna-NM_100674.3 | 308 | 2 | Chr1 | 2483460 - 2484564 |
| 2 | Col0 | I | AtGATA10a | rna-NM_202061.2 | 308 | 2 | Chr1 | 2483460 - 2484564 |
| 3 | Col0 | I | AtGATA11c | rna-NM_100675.4 | 303 | 2 | Chr1 | 2486202 - 2487402 |
| 4 | Col0 | I | AtGATA11b | rna-NM_001084016.2 | 303 | 2 | Chr1 | 2486202 - 2487402 |
| 5 | Col0 | I | AtGATA11a | rna-NM_001331749.1 | 303 | 2 | Chr1 | 2486202 - 2487402 |
| 6 | Col0 | III | AtGATA28b | rna-NM_104038.4 | 302 | 7 | Chr1 | 19133176 - 19135252 |
| 7 | Col0 | III | AtGATA28a | rna-NM_202273.3 | 302 | 7 | Chr1 | 19133176 - 19135252 |
| 8 | Col0 | II | AtGATA20 | rna-NM_127395.3 | 208 | 2 | Chr2 | 7982868 - 7984017 |
| 9 | Col0 | I | AtGATA13 | rna-NM_128393.3 | 291 | 2 | Chr2 | 12103672 - 12106172 |
| 10 | Col0 | I | AtGATA2 | rna-NM_130069.3 | 264 | 2 | Chr2 | 18582958 - 18583845 |
| 11 | Col0 | II | AtGATA15a | rna-NM_111554.5 | 149 | 3 | Chr3 | 2126658 - 2127265 |
| 12 | Col0 | II | AtGATA17 | rna-NM_112563.3 | 190 | 2 | Chr3 | 5763752 - 5764576 |
| 13 | Col0 | II | AtGATA29 | rna-NM_112966.2 | 208 | 3 | Chr3 | 7255016 - 7256140 |
| 14 | Col0 | III | AtGATA24b | rna-NM_180287.5 | 295 | 7 | Chr3 | 7422832 - 7424598 |
| 15 | Col0 | III | AtGATA24a | rna-NM_113012.4 | 297 | 7 | Chr3 | 7422832 - 7424598 |
| 16 | Col0 | I | AtGATA1 | rna-NM_113310.4 | 274 | 2 | Chr3 | 8686060 - 8687462 |
| 17 | Col0 | I | AtGATA14 | rna-NM_114386.2 | 204 | 2 | Chr3 | 16537538 - 16538232 |
| 18 | Col0 | II | AtGATA18 | rna-NM_114947.3 | 295 | 2 | Chr3 | 18911112 - 18912369 |
| 19 | Col0 | I | AtGATA6 | rna-NM_114968.4 | 312 | 2 | Chr3 | 18973639 - 18974668 |
| 20 | Col0 | I | AtGATA8b | rna-NM_180373.3 | 322 | 2 | Chr3 | 20296957 - 20298236 |
| 21 | Col0 | I | AtGATA8a | rna-NM_115338.1 | 322 | 2 | Chr3 | 20296957 - 20298236 |
| 22 | Col0 | I | AtGATA4 | rna-NM_115917.5 | 240 | 2 | Chr3 | 22373348 - 22374147 |
| 23 | Col0 | II | AtGATA30 | rna-NM_148341.5 | 197 | 2 | Chr4 | 9131872 - 9132893 |
| 24 | Col0 | IV | AtGATA26c | rna-NM_001203825.2 | 514 | 8 | Chr4 | 9784329 - 9786974 |
| 25 | Col0 | IV | AtGATA26b | rna-NM_117864.4 | 510 | 8 | Chr4 | 9784329 - 9786974 |
| 26 | Col0 | IV | AtGATA26a | rna-NM_001203826.1 | 526 | 8 | Chr4 | 9784329 - 9786644 |
| 27 | Col0 | III | AtGATA25c | rna-NM_179104.3 | 309 | 7 | Chr4 | 12645785 - 12647734 |
| 28 | Col0 | III | AtGATA25b | rna-NM_118580.4 | 309 | 7 | Chr4 | 12645785 - 12647734 |
| 29 | Col0 | III | AtGATA25a | rna-NM_001203892.1 | 317 | 7 | Chr4 | 12645785 - 12647734 |
| 30 | Col0 | II | AtGATA22 | rna-NM_118748.3 | 352 | 3 | Chr4 | 13253210 - 13254659 |
| 31 | Col0 | I | AtGATA9 | rna-NM_119442.3 | 308 | 2 | Chr4 | 15875598 - 15876615 |
| 32 | Col0 | I | AtGATA3b | rna-NM_001036712.2 | 269 | 2 | Chr4 | 16553700 - 16554610 |
| 33 | Col0 | I | AtGATA3a | rna-NM_119634.4 | 269 | 2 | Chr4 | 16553700 - 16554610 |
| 34 | Col0 | I | AtGATA7 | rna-NM_119792.4 | 238 | 2 | Chr4 | 17147376 - 17148211 |
| 35 | Col0 | II | AtGATA19 | rna-NM_119825.3 | 211 | 2 | Chr4 | 17268906 - 17269662 |
| 36 | Col0 | I | AtGATA12 | rna-NM_122484.3 | 331 | 2 | Chr5 | 9004398 - 9005502 |
| 37 | Col0 | II | AtGATA23 | rna-NM_122575.3 | 120 | 2 | Chr5 | 9479592 - 9480061 |
| 38 | Col0 | IV | AtGATA27 | rna-NM_124085.5 | 470 | 7 | Chr5 | 19145108 - 19147303 |
| 39 | Col0 | II | AtGATA16 | rna-NM_124307.3 | 139 | 3 | Chr5 | 19984849 - 19985475 |
| 40 | Col0 | II | AtGATA21 | rna-NM_125069.3 | 398 | 3 | Chr5 | 22989630 - 22991351 |
| 41 | Col0 | I | AtGATA5b | rna-NM_126030.6 | 339 | 2 | Chr5 | 26496208 - 26497309 |
| 42 | Col0 | I | AtGATA5a | rna-NM_203273.2 | 339 | 2 | Chr5 | 26496208 - 26497309 |
| 43 | Edi0 | I | AtGATA10b | ATEd1G08000.1 | 308 | 2 | Chr1 | 2478170 - 2479274 |
| 44 | Edi0 | I | AtGATA10a | ATEd1G08000.2 | 308 | 2 | Chr1 | 2478170 - 2479274 |
| 45 | Edi0 | I | AtGATA11c | ATEd1G08010.1 | 303 | 2 | Chr1 | 2480922 - 2482121 |
| 46 | Edi0 | I | AtGATA11b | ATEd1G08010.2 | 303 | 2 | Chr1 | 2480922 - 2482121 |
| 47 | Edi0 | III | AtGATA28b | ATEd1G51600.1 | 302 | 7 | Chr1 | 18824537 - 18826613 |
| 48 | Edi0 | III | AtGATA28a | ATEd1G51600.2 | 302 | 7 | Chr1 | 18824537 - 18826613 |
| 49 | Edi0 | II | AtGATA20 | ATEd2G18380.1 | 208 | 2 | Chr2 | 7766854 - 7768003 |
| 50 | Edi0 | I | AtGATA13 | ATEd2G28340.1 | 291 | 2 | Chr2 | 11850270 - 11852770 |
| 51 | Edi0 | I | AtGATA2 | ATEd2G45050.1 | 264 | 2 | Chr2 | 18304881 - 18305768 |
| 52 | Edi0 | II | AtGATA15a | ATEd3G06740.1 | 150 | 3 | Chr3 | 2132498 - 2133108 |
| 53 | Edi0 | II | AtGATA17 | ATEd3G16870.1 | 190 | 2 | Chr3 | 5749970 - 5750794 |
| 54 | Edi0 | II | AtGATA29 | ATEd3G20750.1 | 208 | 3 | Chr3 | 7237541 - 7238665 |
| 55 | Edi0 | III | AtGATA24a | ATEd3G21175.1 | 297 | 7 | Chr3 | 7405316 - 7407082 |
| 56 | Edi0 | III | AtGATA24b | ATEd3G21175.2 | 295 | 7 | Chr3 | 7405316 - 7407082 |
| 57 | Edi0 | I | AtGATA1 | ATEd3G24050.1 | 274 | 2 | Chr3 | 8647320 - 8648722 |
| 58 | Edi0 | I | AtGATA14 | ATEd3G45170.1 | 187 | 2 | Chr3 | 16279364 - 16280007 |
| 59 | Edi0 | II | AtGATA18 | ATEd3G50870.1 | 295 | 2 | Chr3 | 18620356 - 18621613 |
| 60 | Edi0 | I | AtGATA6 | ATEd3G51080.1 | 312 | 2 | Chr3 | 18683121 - 18684150 |
| 61 | Edi0 | I | AtGATA8a | ATEd3G54810.1 | 322 | 2 | Chr3 | 20002878 - 20004157 |
| 62 | Edi0 | I | AtGATA8b | ATEd3G54810.2 | 322 | 2 | Chr3 | 20002878 - 20004157 |
| 63 | Edi0 | I | AtGATA4 | ATEd3G60530.1 | 240 | 2 | Chr3 | 22068588 - 22069387 |
| 64 | Edi0 | II | AtGATA30 | ATEd4G16141.1 | 197 | 2 | Chr4 | 8932176 - 8933197 |
| 65 | Edi0 | IV | AtGATA26b | ATEd4G17570.1 | 510 | 8 | Chr4 | 9572164 - 9574809 |
| 66 | Edi0 | IV | AtGATA26a | ATEd4G17570.2 | 526 | 8 | Chr4 | 9572164 - 9574479 |
| 67 | Edi0 | IV | AtGATA26c | ATEd4G17570.3 | 514 | 8 | Chr4 | 9572164 - 9574809 |
| 68 | Edi0 | III | AtGATA25b | ATEd4G24470.1 | 309 | 7 | Chr4 | 12424560 - 12426509 |
| 69 | Edi0 | III | AtGATA25c | ATEd4G24470.2 | 309 | 7 | Chr4 | 12424560 - 12426509 |
| 70 | Edi0 | III | AtGATA25a | ATEd4G24470.3 | 317 | 7 | Chr4 | 12424560 - 12426509 |
| 71 | Edi0 | II | AtGATA22 | ATEd4G26150.1 | 351 | 3 | Chr4 | 13024634 - 13026081 |
| 72 | Edi0 | I | AtGATA9 | ATEd4G32890.1 | 308 | 2 | Chr4 | 15615784 - 15616801 |
| 73 | Edi0 | I | AtGATA3a | ATEd4G34680.1 | 269 | 2 | Chr4 | 16292060 - 16292970 |
| 74 | Edi0 | I | AtGATA3b | ATEd4G34680.2 | 269 | 2 | Chr4 | 16292060 - 16292970 |
| 75 | Edi0 | I | AtGATA7 | ATEd4G36240.1 | 238 | 2 | Chr4 | 16881707 - 16882539 |
| 76 | Edi0 | II | AtGATA19 | ATEd4G36620.1 | 211 | 2 | Chr4 | 17003241 - 17003997 |
| 77 | Edi0 | I | AtGATA12 | ATEd5G25830.1 | 332 | 2 | Chr5 | 8889727 - 8890834 |
| 78 | Edi0 | II | AtGATA23 | ATEd5G26930.1 | 120 | 2 | Chr5 | 9350369 - 9350838 |
| 79 | Edi0 | IV | AtGATA27 | ATEd5G47140.1 | 470 | 7 | Chr5 | 18738619 - 18740820 |
| 80 | Edi0 | II | AtGATA16 | ATEd5G49300.1 | 139 | 3 | Chr5 | 19556871 - 19557497 |
| 81 | Edi0 | II | AtGATA21 | ATEd5G56860.1 | 399 | 3 | Chr5 | 22525558 - 22527282 |
| 82 | Edi0 | I | AtGATA5b | ATEd5G66320.1 | 339 | 2 | Chr5 | 26015840 - 26016941 |
| 83 | Edi0 | I | AtGATA5a | ATEd5G66320.2 | 339 | 2 | Chr5 | 26015840 - 26016941 |
| 84 | Ct1 | I | AtGATA10b | ATCt1G08000.1 | 308 | 2 | Chr1 | 2474220 - 2475324 |
| 85 | Ct1 | I | AtGATA10a | ATCt1G08000.2 | 308 | 2 | Chr1 | 2474220 - 2475324 |
| 86 | Ct1 | I | AtGATA11c | ATCt1G08010.1 | 303 | 2 | Chr1 | 2476972 - 2478171 |
| 87 | Ct1 | I | AtGATA11b | ATCt1G08010.2 | 303 | 2 | Chr1 | 2476972 - 2478171 |
| 88 | Ct1 | III | AtGATA28b | ATCt1G51600.1 | 302 | 7 | Chr1 | 18866560 - 18868636 |
| 89 | Ct1 | III | AtGATA28a | ATCt1G51600.2 | 302 | 7 | Chr1 | 18866560 - 18868636 |
| 90 | Ct1 | II | AtGATA20 | ATCt2G18380.1 | 208 | 2 | Chr2 | 7791456 - 7792605 |
| 91 | Ct1 | I | AtGATA13 | ATCt2G28340.1 | 291 | 2 | Chr2 | 11865188 - 11867688 |
| 92 | Ct1 | I | AtGATA2 | ATCt2G45050.1 | 264 | 2 | Chr2 | 18302294 - 18303190 |
| 93 | Ct1 | II | AtGATA15a | ATCt3G06740.1 | 150 | 3 | Chr3 | 2124577 - 2125187 |
| 94 | Ct1 | II | AtGATA17 | ATCt3G16870.1 | 190 | 2 | Chr3 | 5751519 - 5752343 |
| 95 | Ct1 | II | AtGATA29 | ATCt3G20750.1 | 208 | 3 | Chr3 | 7234963 - 7236087 |
| 96 | Ct1 | III | AtGATA24a | ATCt3G21175.1 | 297 | 7 | Chr3 | 7400839 - 7402605 |
| 97 | Ct1 | III | AtGATA24b | ATCt3G21175.2 | 295 | 7 | Chr3 | 7400839 - 7402605 |
| 98 | Ct1 | I | AtGATA1 | ATCt3G24050.1 | 274 | 2 | Chr3 | 8645606 - 8647008 |
| 99 | Ct1 | I | AtGATA14 | ATCt3G45170.1 | 192 | 2 | Chr3 | 16151174 - 16151832 |
| 100 | Ct1 | II | AtGATA18 | ATCt3G50870.1 | 295 | 2 | Chr3 | 18469352 - 18470611 |
| 101 | Ct1 | I | AtGATA6 | ATCt3G51080.1 | 312 | 2 | Chr3 | 18531728 - 18532757 |
| 102 | Ct1 | I | AtGATA8a | ATCt3G54810.1 | 322 | 2 | Chr3 | 19851311 - 19852583 |
| 103 | Ct1 | I | AtGATA8b | ATCt3G54810.2 | 322 | 2 | Chr3 | 19851311 - 19852583 |
| 104 | Ct1 | I | AtGATA4 | ATCt3G60530.1 | 240 | 2 | Chr3 | 21910751 - 21911550 |
| 105 | Ct1 | II | AtGATA30 | ATCt4G16141.1 | 197 | 2 | Chr4 | 8870710 - 8871731 |
| 106 | Ct1 | IV | AtGATA26b | ATCt4G17570.1 | 510 | 8 | Chr4 | 9508658 - 9511303 |
| 107 | Ct1 | IV | AtGATA26a | ATCt4G17570.2 | 526 | 8 | Chr4 | 9508658 - 9510973 |
| 108 | Ct1 | IV | AtGATA26c | ATCt4G17570.3 | 514 | 8 | Chr4 | 9508658 - 9511303 |
| 109 | Ct1 | III | AtGATA25b | ATCt4G24470.1 | 309 | 7 | Chr4 | 12325231 - 12327180 |
| 110 | Ct1 | III | AtGATA25c | ATCt4G24470.2 | 309 | 7 | Chr4 | 12325231 - 12327180 |
| 111 | Ct1 | III | AtGATA25a | ATCt4G24470.3 | 317 | 7 | Chr4 | 12325231 - 12327180 |
| 112 | Ct1 | II | AtGATA22 | ATCt4G26150.1 | 352 | 3 | Chr4 | 12929694 - 12931143 |
| 113 | Ct1 | I | AtGATA9 | ATCt4G32890.1 | 308 | 2 | Chr4 | 15547702 - 15548719 |
| 114 | Ct1 | I | AtGATA3a | ATCt4G34680.1 | 269 | 2 | Chr4 | 16225629 - 16226539 |
| 115 | Ct1 | I | AtGATA3b | ATCt4G34680.2 | 269 | 2 | Chr4 | 16225629 - 16226539 |
| 116 | Ct1 | I | AtGATA7 | ATCt4G36240.1 | 238 | 2 | Chr4 | 16818028 - 16818850 |
| 117 | Ct1 | II | AtGATA19 | ATCt4G36620.1 | 211 | 2 | Chr4 | 16939965 - 16940721 |
| 118 | Ct1 | I | AtGATA12 | ATCt5G25830.1 | 331 | 2 | Chr5 | 8884506 - 8885610 |
| 119 | Ct1 | II | AtGATA23 | ATCt5G26930.1 | 120 | 2 | Chr5 | 9351819 - 9352288 |
| 120 | Ct1 | IV | AtGATA27 | ATCt5G47140.1 | 470 | 7 | Chr5 | 18694286 - 18696487 |
| 121 | Ct1 | II | AtGATA16 | ATCt5G49300.1 | 139 | 3 | Chr5 | 19511745 - 19512372 |
| 122 | Ct1 | II | AtGATA21 | ATCt5G56860.1 | 398 | 3 | Chr5 | 22490843 - 22492564 |
| 123 | Ct1 | I | AtGATA5b | ATCt5G66320.1 | 339 | 2 | Chr5 | 25976678 - 25977779 |
| 124 | Ct1 | I | AtGATA5a | ATCt5G66320.2 | 339 | 2 | Chr5 | 25976678 - 25977779 |
| 125 | Can0 | I | AtGATA10b | ATCn1G08000.1 | 308 | 2 | Chr1 | 2475729 - 2476833 |
| 126 | Can0 | I | AtGATA10a | ATCn1G08000.2 | 308 | 2 | Chr1 | 2475729 - 2476833 |
| 127 | Can0 | I | AtGATA11c | ATCn1G08010.1 | 303 | 2 | Chr1 | 2478471 - 2479671 |
| 128 | Can0 | I | AtGATA11b | ATCn1G08010.2 | 303 | 2 | Chr1 | 2478471 - 2479671 |
| 129 | Can0 | III | AtGATA28b | ATCn1G51600.1 | 302 | 7 | Chr1 | 18788706 - 18790782 |
| 130 | Can0 | III | AtGATA28a | ATCn1G51600.2 | 302 | 7 | Chr1 | 18788706 - 18790782 |
| 131 | Can0 | II | AtGATA20 | ATCn2G18380.1 | 208 | 2 | Chr2 | 7736882 - 7738031 |
| 132 | Can0 | I | AtGATA13 | ATCn2G28340.1 | 291 | 2 | Chr2 | 11828455 - 11831035 |
| 133 | Can0 | I | AtGATA2 | ATCn2G45050.1 | 265 | 2 | Chr2 | 18269012 - 18269902 |
| 134 | Can0 | II | AtGATA15a | ATCn3G06740.1 | 150 | 3 | Chr3 | 2127413 - 2128023 |
| 135 | Can0 | II | AtGATA17 | ATCn3G16870.1 | 190 | 2 | Chr3 | 5752092 - 5752916 |
| 136 | Can0 | II | AtGATA29 | ATCn3G20750.1 | 208 | 3 | Chr3 | 7240514 - 7241638 |
| 137 | Can0 | III | AtGATA24a | ATCn3G21175.1 | 297 | 7 | Chr3 | 7403450 - 7405216 |
| 138 | Can0 | III | AtGATA24b | ATCn3G21175.2 | 295 | 7 | Chr3 | 7403450 - 7405216 |
| 139 | Can0 | I | AtGATA1 | ATCn3G24050.1 | 274 | 2 | Chr3 | 8634044 - 8635447 |
| 140 | Can0 | I | AtGATA14 | ATCn3G45170.1 | 192 | 2 | Chr3 | 16172480 - 16173138 |
| 141 | Can0 | II | AtGATA18 | ATCn3G50870.1 | 295 | 2 | Chr3 | 18508062 - 18509318 |
| 142 | Can0 | I | AtGATA6 | ATCn3G51080.1 | 312 | 2 | Chr3 | 18570737 - 18571766 |
| 143 | Can0 | I | AtGATA8a | ATCn3G54810.1 | 322 | 2 | Chr3 | 19886335 - 19887607 |
| 144 | Can0 | I | AtGATA8b | ATCn3G54810.2 | 322 | 2 | Chr3 | 19886335 - 19887607 |
| 145 | Can0 | I | AtGATA4 | ATCn3G60530.1 | 240 | 2 | Chr3 | 21949444 - 21950243 |
| 146 | Can0 | II | AtGATA30 | ATCn4G16141.1 | 197 | 2 | Chr4 | 8736071 - 8737091 |
| 147 | Can0 | IV | AtGATA26b | ATCn4G17570.1 | 510 | 8 | Chr4 | 9368465 - 9371108 |
| 148 | Can0 | IV | AtGATA26a | ATCn4G17570.2 | 526 | 8 | Chr4 | 9368465 - 9370778 |
| 149 | Can0 | IV | AtGATA26c | ATCn4G17570.3 | 514 | 8 | Chr4 | 9368465 - 9371108 |
| 150 | Can0 | III | AtGATA25b | ATCn4G24470.1 | 309 | 7 | Chr4 | 12189872 - 12191821 |
| 151 | Can0 | III | AtGATA25c | ATCn4G24470.2 | 309 | 7 | Chr4 | 12189872 - 12191821 |
| 152 | Can0 | III | AtGATA25a | ATCn4G24470.3 | 317 | 7 | Chr4 | 12189872 - 12191821 |
| 153 | Can0 | II | AtGATA22 | ATCn4G26150.1 | 351 | 3 | Chr4 | 12789876 - 12791322 |
| 154 | Can0 | I | AtGATA9 | ATCn4G32890.1 | 306 | 2 | Chr4 | 15400805 - 15401816 |
| 155 | Can0 | I | AtGATA3a | ATCn4G34680.1 | 269 | 2 | Chr4 | 16078497 - 16079407 |
| 156 | Can0 | I | AtGATA3b | ATCn4G34680.2 | 269 | 2 | Chr4 | 16078497 - 16079407 |
| 157 | Can0 | I | AtGATA7 | ATCn4G36240.1 | 238 | 2 | Chr4 | 16669463 - 16670298 |
| 158 | Can0 | II | AtGATA19 | ATCn4G36620.1 | 211 | 2 | Chr4 | 16792047 - 16792803 |
| 159 | Can0 | I | AtGATA12 | ATCn5G25830.1 | 332 | 2 | Chr5 | 8862088 - 8863195 |
| 160 | Can0 | II | AtGATA23 | ATCn5G26930.1 | 120 | 2 | Chr5 | 9304223 - 9304692 |
| 161 | Can0 | IV | AtGATA27 | ATCn5G47140.1 | 472 | 7 | Chr5 | 18657891 - 18660098 |
| 162 | Can0 | II | AtGATA16 | ATCn5G49300.1 | 139 | 3 | Chr5 | 19456989 - 19457615 |
| 163 | Can0 | II | AtGATA21 | ATCn5G56860.1 | 398 | 3 | Chr5 | 22438779 - 22440500 |
| 164 | Can0 | I | AtGATA5b | ATCn5G66320.1 | 339 | 2 | Chr5 | 25936638 - 25937739 |
| 165 | Can0 | I | AtGATA5a | ATCn5G66320.2 | 339 | 2 | Chr5 | 25936638 - 25937739 |
| 166 | Bur0 | I | AtGATA10b | ATBr1G08000.1 | 308 | 2 | Chr1 | 2469259 - 2470364 |
| 167 | Bur0 | I | AtGATA10a | ATBr1G08000.2 | 308 | 2 | Chr1 | 2469259 - 2470364 |
| 168 | Bur0 | I | AtGATA11c | ATBr1G08010.1 | 303 | 2 | Chr1 | 2472008 - 2473207 |
| 169 | Bur0 | I | AtGATA11b | ATBr1G08010.2 | 303 | 2 | Chr1 | 2472008 - 2473207 |
| 170 | Bur0 | III | AtGATA28b | ATBr1G51600.1 | 302 | 7 | Chr1 | 18776407 - 18778483 |
| 171 | Bur0 | III | AtGATA28a | ATBr1G51600.2 | 302 | 7 | Chr1 | 18776407 - 18778483 |
| 172 | Bur0 | II | AtGATA20 | ATBr2G18380.1 | 208 | 2 | Chr2 | 7739045 - 7740194 |
| 173 | Bur0 | I | AtGATA13 | ATBr2G28340.1 | 291 | 2 | Chr2 | 11806600 - 11809183 |
| 174 | Bur0 | I | AtGATA2 | ATBr2G45050.1 | 265 | 2 | Chr2 | 18229141 - 18230031 |
| 175 | Bur0 | II | AtGATA15a | ATBr3G06740.1 | 150 | 3 | Chr3 | 2124019 - 2124629 |
| 176 | Bur0 | II | AtGATA17 | ATBr3G16870.1 | 189 | 2 | Chr3 | 5738932 - 5739753 |
| 177 | Bur0 | II | AtGATA29 | ATBr3G20750.1 | 208 | 3 | Chr3 | 7224772 - 7225896 |
| 178 | Bur0 | III | AtGATA24a | ATBr3G21175.1 | 297 | 7 | Chr3 | 7385253 - 7387012 |
| 179 | Bur0 | III | AtGATA24b | ATBr3G21175.2 | 295 | 7 | Chr3 | 7385253 - 7387012 |
| 180 | Bur0 | I | AtGATA1 | ATBr3G24050.1 | 274 | 2 | Chr3 | 8626768 - 8628171 |
| 181 | Bur0 | I | AtGATA14 | ATBr3G45170.1 | 192 | 2 | Chr3 | 16246679 - 16247337 |
| 182 | Bur0 | II | AtGATA18 | ATBr3G50870.1 | 295 | 2 | Chr3 | 18582289 - 18583548 |
| 183 | Bur0 | I | AtGATA6 | ATBr3G51080.1 | 312 | 2 | Chr3 | 18645024 - 18646053 |
| 184 | Bur0 | I | AtGATA8a | ATBr3G54810.1 | 322 | 2 | Chr3 | 19958777 - 19960057 |
| 185 | Bur0 | I | AtGATA8b | ATBr3G54810.2 | 322 | 2 | Chr3 | 19958777 - 19960057 |
| 186 | Bur0 | I | AtGATA4 | ATBr3G60530.1 | 240 | 2 | Chr3 | 22021532 - 22022331 |
| 187 | Bur0 | II | AtGATA30 | ATBr4G16141.1 | 197 | 2 | Chr4 | 8854523 - 8855544 |
| 188 | Bur0 | IV | AtGATA26b | ATBr4G17570.1 | 510 | 8 | Chr4 | 9502420 - 9505063 |
| 189 | Bur0 | IV | AtGATA26a | ATBr4G17570.2 | 526 | 8 | Chr4 | 9502420 - 9504730 |
| 190 | Bur0 | IV | AtGATA26c | ATBr4G17570.3 | 514 | 8 | Chr4 | 9502420 - 9505063 |
| 191 | Bur0 | III | AtGATA25b | ATBr4G24470.1 | 309 | 7 | Chr4 | 12341311 - 12343260 |
| 192 | Bur0 | III | AtGATA25c | ATBr4G24470.2 | 309 | 7 | Chr4 | 12341311 - 12343260 |
| 193 | Bur0 | III | AtGATA25a | ATBr4G24470.3 | 317 | 7 | Chr4 | 12341311 - 12343260 |
| 194 | Bur0 | II | AtGATA22 | ATBr4G26150.1 | 351 | 3 | Chr4 | 12947553 - 12948999 |
| 195 | Bur0 | I | AtGATA9 | ATBr4G32890.1 | 308 | 2 | Chr4 | 15558890 - 15559907 |
| 196 | Bur0 | I | AtGATA3a | ATBr4G34680.1 | 269 | 2 | Chr4 | 16237463 - 16238373 |
| 197 | Bur0 | I | AtGATA3b | ATBr4G34680.2 | 269 | 2 | Chr4 | 16237463 - 16238373 |
| 198 | Bur0 | I | AtGATA7 | ATBr4G36240.1 | 238 | 2 | Chr4 | 16828200 - 16829022 |
| 199 | Bur0 | II | AtGATA19 | ATBr4G36620.1 | 211 | 2 | Chr4 | 16949525 - 16950281 |
| 200 | Bur0 | I | AtGATA12 | ATBr5G25830.1 | 331 | 2 | Chr5 | 8932440 - 8933544 |
| 201 | Bur0 | II | AtGATA23 | ATBr5G26930.1 | 120 | 2 | Chr5 | 9408401 - 9408870 |
| 202 | Bur0 | IV | AtGATA27 | ATBr5G47140.1 | 470 | 7 | Chr5 | 18846714 - 18848915 |
| 203 | Bur0 | II | AtGATA16 | ATBr5G49300.1 | 139 | 3 | Chr5 | 19675984 - 19676610 |
| 204 | Bur0 | II | AtGATA21 | ATBr5G56860.1 | 398 | 3 | Chr5 | 22652817 - 22654538 |
| 205 | Bur0 | I | AtGATA5b | ATBr5G66320.1 | 339 | 2 | Chr5 | 26147355 - 26148456 |
| 206 | Bur0 | I | AtGATA5a | ATBr5G66320.2 | 339 | 2 | Chr5 | 26147355 - 26148456 |
| 207 | Hi0 | I | AtGATA10b | ATHi1G08000.1 | 308 | 2 | Chr1 | 2477701 - 2478805 |
| 208 | Hi0 | I | AtGATA10a | ATHi1G08000.2 | 308 | 2 | Chr1 | 2477701 - 2478805 |
| 209 | Hi0 | I | AtGATA11c | ATHi1G08010.1 | 303 | 2 | Chr1 | 2480453 - 2481652 |
| 210 | Hi0 | I | AtGATA11b | ATHi1G08010.2 | 303 | 2 | Chr1 | 2480453 - 2481652 |
| 211 | Hi0 | III | AtGATA28b | ATHi1G51600.1 | 302 | 7 | Chr1 | 18882314 - 18884390 |
| 212 | Hi0 | III | AtGATA28a | ATHi1G51600.2 | 302 | 7 | Chr1 | 18882314 - 18884390 |
| 213 | Hi0 | II | AtGATA20 | ATHi2G18380.1 | 208 | 2 | Chr2 | 7893852 - 7895001 |
| 214 | Hi0 | I | AtGATA13 | ATHi2G28340.1 | 291 | 2 | Chr2 | 11993771 - 11996351 |
| 215 | Hi0 | I | AtGATA2 | ATHi2G45050.1 | 265 | 2 | Chr2 | 18458959 - 18459849 |
| 216 | Hi0 | II | AtGATA15a | ATHi3G06740.1 | 150 | 3 | Chr3 | 2126939 - 2127549 |
| 217 | Hi0 | II | AtGATA17 | ATHi3G16870.1 | 190 | 2 | Chr3 | 5746314 - 5747138 |
| 218 | Hi0 | II | AtGATA29 | ATHi3G20750.1 | 208 | 3 | Chr3 | 7232346 - 7233470 |
| 219 | Hi0 | I | AtGATA1 | ATHi3G24050.1 | 274 | 2 | Chr3 | 8658752 - 8660155 |
| 220 | Hi0 | I | AtGATA14 | ATHi3G45170.1 | 192 | 2 | Chr3 | 16352173 - 16352831 |
| 221 | Hi0 | II | AtGATA18 | ATHi3G50870.1 | 296 | 2 | Chr3 | 18672335 - 18673595 |
| 222 | Hi0 | I | AtGATA6 | ATHi3G51080.1 | 312 | 2 | Chr3 | 18734570 - 18735600 |
| 223 | Hi0 | I | AtGATA8a | ATHi3G54810.1 | 322 | 2 | Chr3 | 20053353 - 20054632 |
| 224 | Hi0 | I | AtGATA8b | ATHi3G54810.2 | 322 | 2 | Chr3 | 20053353 - 20054632 |
| 225 | Hi0 | I | AtGATA4 | ATHi3G60530.1 | 240 | 2 | Chr3 | 22104784 - 22105583 |
| 226 | Hi0 | II | AtGATA30 | ATHi4G16141.1 | 197 | 2 | Chr4 | 8944364 - 8945409 |
| 227 | Hi0 | IV | AtGATA26b | ATHi4G17570.1 | 510 | 8 | Chr4 | 9589905 - 9592550 |
| 228 | Hi0 | IV | AtGATA26a | ATHi4G17570.2 | 515 | 8 | Chr4 | 9589905 - 9592187 |
| 229 | Hi0 | IV | AtGATA26c | ATHi4G17570.3 | 514 | 8 | Chr4 | 9589905 - 9592550 |
| 230 | Hi0 | III | AtGATA25b | ATHi4G24470.1 | 309 | 7 | Chr4 | 12426434 - 12428383 |
| 231 | Hi0 | III | AtGATA25c | ATHi4G24470.2 | 309 | 7 | Chr4 | 12426434 - 12428383 |
| 232 | Hi0 | III | AtGATA25a | ATHi4G24470.3 | 317 | 7 | Chr4 | 12426434 - 12428383 |
| 233 | Hi0 | II | AtGATA22 | ATHi4G26150.1 | 351 | 3 | Chr4 | 13032103 - 13033549 |
| 234 | Hi0 | I | AtGATA9 | ATHi4G32890.1 | 308 | 2 | Chr4 | 15645824 - 15646841 |
| 235 | Hi0 | I | AtGATA3a | ATHi4G34680.1 | 269 | 2 | Chr4 | 16322363 - 16323273 |
| 236 | Hi0 | I | AtGATA3b | ATHi4G34680.2 | 269 | 2 | Chr4 | 16322363 - 16323273 |
| 237 | Hi0 | I | AtGATA7 | ATHi4G36240.1 | 238 | 2 | Chr4 | 16912729 - 16913564 |
| 238 | Hi0 | II | AtGATA19 | ATHi4G36620.1 | 211 | 2 | Chr4 | 17034784 - 17035540 |
| 239 | Hi0 | I | AtGATA12 | ATHi5G25830.1 | 331 | 2 | Chr5 | 8929493 - 8930597 |
| 240 | Hi0 | II | AtGATA23 | ATHi5G26930.1 | 120 | 2 | Chr5 | 9400703 - 9401172 |
| 241 | Hi0 | IV | AtGATA27 | ATHi5G47140.1 | 470 | 7 | Chr5 | 18913591 - 18915792 |
| 242 | Hi0 | II | AtGATA16 | ATHi5G49300.1 | 139 | 3 | Chr5 | 19734809 - 19735435 |
| 243 | Hi0 | II | AtGATA21 | ATHi5G56860.1 | 398 | 3 | Chr5 | 22713821 - 22715542 |
| 244 | Hi0 | I | AtGATA5b | ATHi5G66320.1 | 339 | 2 | Chr5 | 26206486 - 26207587 |
| 245 | Hi0 | I | AtGATA5a | ATHi5G66320.2 | 339 | 2 | Chr5 | 26206486 - 26207587 |
| 246 | Kn0 | I | AtGATA10b | ATKn1G08000.1 | 308 | 2 | Chr1 | 2476800 - 2477904 |
| 247 | Kn0 | I | AtGATA10a | ATKn1G08000.2 | 308 | 2 | Chr1 | 2476800 - 2477904 |
| 248 | Kn0 | I | AtGATA11c | ATKn1G08010.1 | 303 | 2 | Chr1 | 2479542 - 2480742 |
| 249 | Kn0 | I | AtGATA11b | ATKn1G08010.2 | 303 | 2 | Chr1 | 2479542 - 2480742 |
| 250 | Kn0 | III | AtGATA28b | ATKn1G51600.1 | 302 | 7 | Chr1 | 18806111 - 18808187 |
| 251 | Kn0 | III | AtGATA28a | ATKn1G51600.2 | 302 | 7 | Chr1 | 18806111 - 18808187 |
| 252 | Kn0 | II | AtGATA20 | ATKn2G18380.1 | 208 | 2 | Chr2 | 7778853 - 7780002 |
| 253 | Kn0 | I | AtGATA13 | ATKn2G28340.1 | 291 | 2 | Chr2 | 11861402 - 11863983 |
| 254 | Kn0 | I | AtGATA2 | ATKn2G45050.1 | 263 | 2 | Chr2 | 18296721 - 18297614 |
| 255 | Kn0 | II | AtGATA15a | ATKn3G06740.1 | 150 | 3 | Chr3 | 2129571 - 2130181 |
| 256 | Kn0 | II | AtGATA15b | ATKn3G06740.2 | 137 | 2 | Chr3 | 2129694 - 2130181 |
| 257 | Kn0 | II | AtGATA17 | ATKn3G16870.1 | 190 | 2 | Chr3 | 5729972 - 5730796 |
| 258 | Kn0 | II | AtGATA29 | ATKn3G20750.1 | 208 | 3 | Chr3 | 7212485 - 7213609 |
| 259 | Kn0 | III | AtGATA24a | ATKn3G21175.1 | 297 | 7 | Chr3 | 7379171 - 7380937 |
| 260 | Kn0 | III | AtGATA24b | ATKn3G21175.2 | 295 | 7 | Chr3 | 7379171 - 7380937 |
| 261 | Kn0 | I | AtGATA1 | ATKn3G24050.1 | 274 | 2 | Chr3 | 8618926 - 8620329 |
| 262 | Kn0 | I | AtGATA14 | ATKn3G45170.1 | 192 | 2 | Chr3 | 16221745 - 16222403 |
| 263 | Kn0 | II | AtGATA18 | ATKn3G50870.1 | 295 | 2 | Chr3 | 18571050 - 18572307 |
| 264 | Kn0 | I | AtGATA6 | ATKn3G51080.1 | 312 | 2 | Chr3 | 18633339 - 18634369 |
| 265 | Kn0 | I | AtGATA8a | ATKn3G54810.1 | 322 | 2 | Chr3 | 19948553 - 19949831 |
| 266 | Kn0 | I | AtGATA8b | ATKn3G54810.2 | 322 | 2 | Chr3 | 19948553 - 19949831 |
| 267 | Kn0 | I | AtGATA4 | ATKn3G60530.1 | 240 | 2 | Chr3 | 21997113 - 21997912 |
| 268 | Kn0 | II | AtGATA30 | ATKn4G16141.1 | 197 | 2 | Chr4 | 8866658 - 8867703 |
| 269 | Kn0 | IV | AtGATA26b | ATKn4G17570.1 | 510 | 8 | Chr4 | 9499951 - 9502599 |
| 270 | Kn0 | IV | AtGATA26a | ATKn4G17570.2 | 526 | 8 | Chr4 | 9499951 - 9502266 |
| 271 | Kn0 | IV | AtGATA26c | ATKn4G17570.3 | 514 | 8 | Chr4 | 9499951 - 9502599 |
| 272 | Kn0 | III | AtGATA25b | ATKn4G24470.1 | 309 | 7 | Chr4 | 12332167 - 12334116 |
| 273 | Kn0 | III | AtGATA25c | ATKn4G24470.2 | 309 | 7 | Chr4 | 12332167 - 12334116 |
| 274 | Kn0 | III | AtGATA25a | ATKn4G24470.3 | 317 | 7 | Chr4 | 12332167 - 12334116 |
| 275 | Kn0 | II | AtGATA22 | ATKn4G26150.1 | 351 | 3 | Chr4 | 12938638 - 12940084 |
| 276 | Kn0 | I | AtGATA9 | ATKn4G32890.1 | 308 | 2 | Chr4 | 15554128 - 15555145 |
| 277 | Kn0 | I | AtGATA3a | ATKn4G34680.1 | 269 | 2 | Chr4 | 16230375 - 16231285 |
| 278 | Kn0 | I | AtGATA3b | ATKn4G34680.2 | 269 | 2 | Chr4 | 16230375 - 16231285 |
| 279 | Kn0 | I | AtGATA7 | ATKn4G36240.1 | 238 | 2 | Chr4 | 16823821 - 16824656 |
| 280 | Kn0 | II | AtGATA19 | ATKn4G36620.1 | 211 | 2 | Chr4 | 16945519 - 16946275 |
| 281 | Kn0 | I | AtGATA12 | ATKn5G25830.1 | 331 | 2 | Chr5 | 8921630 - 8922734 |
| 282 | Kn0 | II | AtGATA23 | ATKn5G26930.1 | 120 | 2 | Chr5 | 9383216 - 9383686 |
| 283 | Kn0 | IV | AtGATA27 | ATKn5G47140.1 | 470 | 7 | Chr5 | 18807397 - 18809598 |
| 284 | Kn0 | II | AtGATA16 | ATKn5G49300.1 | 139 | 3 | Chr5 | 19631938 - 19632564 |
| 285 | Kn0 | II | AtGATA21 | ATKn5G56860.1 | 398 | 3 | Chr5 | 22598882 - 22600603 |
| 286 | Kn0 | I | AtGATA5b | ATKn5G66320.1 | 339 | 2 | Chr5 | 26090566 - 26091667 |
| 287 | Kn0 | I | AtGATA5a | ATKn5G66320.2 | 339 | 2 | Chr5 | 26090566 - 26091667 |
| 288 | Ler0 | I | AtGATA10b | ATLr1G08000.1 | 308 | 2 | Chr1 | 2479324 - 2480428 |
| 289 | Ler0 | I | AtGATA10a | ATLr1G08000.2 | 308 | 2 | Chr1 | 2479324 - 2480428 |
| 290 | Ler0 | I | AtGATA11c | ATLr1G08010.1 | 303 | 2 | Chr1 | 2482067 - 2483266 |
| 291 | Ler0 | I | AtGATA11b | ATLr1G08010.2 | 303 | 2 | Chr1 | 2482067 - 2483266 |
| 292 | Ler0 | III | AtGATA28b | ATLr1G51600.1 | 302 | 7 | Chr1 | 18839233 - 18841309 |
| 293 | Ler0 | III | AtGATA28a | ATLr1G51600.2 | 302 | 7 | Chr1 | 18839233 - 18841309 |
| 294 | Ler0 | II | AtGATA20 | ATLr2G18380.1 | 208 | 2 | Chr2 | 7729800 - 7730949 |
| 295 | Ler0 | I | AtGATA13 | ATLr2G28340.1 | 291 | 2 | Chr2 | 11798645 - 11801145 |
| 296 | Ler0 | I | AtGATA2 | ATLr2G45050.1 | 264 | 2 | Chr2 | 18235414 - 18236301 |
| 297 | Ler0 | II | AtGATA15a | ATLr3G06740.1 | 150 | 3 | Chr3 | 2123278 - 2123888 |
| 298 | Ler0 | II | AtGATA17 | ATLr3G16870.1 | 190 | 2 | Chr3 | 5741722 - 5742546 |
| 299 | Ler0 | II | AtGATA29 | ATLr3G20750.1 | 208 | 3 | Chr3 | 7227406 - 7228530 |
| 300 | Ler0 | I | AtGATA1 | ATLr3G24050.1 | 274 | 2 | Chr3 | 8639866 - 8641269 |
| 301 | Ler0 | I | AtGATA14 | ATLr3G45170.1 | 192 | 2 | Chr3 | 16208244 - 16208902 |
| 302 | Ler0 | II | AtGATA18 | ATLr3G50870.1 | 295 | 2 | Chr3 | 18552108 - 18553365 |
| 303 | Ler0 | I | AtGATA6 | ATLr3G51080.1 | 312 | 2 | Chr3 | 18614965 - 18615994 |
| 304 | Ler0 | I | AtGATA8a | ATLr3G54810.1 | 322 | 2 | Chr3 | 19934778 - 19936057 |
| 305 | Ler0 | I | AtGATA8b | ATLr3G54810.2 | 322 | 2 | Chr3 | 19934778 - 19936057 |
| 306 | Ler0 | I | AtGATA4 | ATLr3G60530.1 | 240 | 2 | Chr3 | 22003068 - 22003867 |
| 307 | Ler0 | II | AtGATA30 | ATLr4G16141.1 | 197 | 2 | Chr4 | 8822333 - 8823354 |
| 308 | Ler0 | IV | AtGATA26b | ATLr4G17570.1 | 510 | 8 | Chr4 | 9466815 - 9469463 |
| 309 | Ler0 | IV | AtGATA26a | ATLr4G17570.2 | 526 | 8 | Chr4 | 9466815 - 9469130 |
| 310 | Ler0 | IV | AtGATA26c | ATLr4G17570.3 | 514 | 8 | Chr4 | 9466815 - 9469463 |
| 311 | Ler0 | III | AtGATA25b | ATLr4G24470.1 | 309 | 7 | Chr4 | 12297815 - 12299764 |
| 312 | Ler0 | III | AtGATA25c | ATLr4G24470.2 | 309 | 7 | Chr4 | 12297815 - 12299764 |
| 313 | Ler0 | III | AtGATA25a | ATLr4G24470.3 | 317 | 7 | Chr4 | 12297815 - 12299764 |
| 314 | Ler0 | II | AtGATA22 | ATLr4G26150.1 | 351 | 3 | Chr4 | 12903986 - 12905432 |
| 315 | Ler0 | I | AtGATA9 | ATLr4G32890.1 | 308 | 2 | Chr4 | 15524215 - 15525232 |
| 316 | Ler0 | I | AtGATA3a | ATLr4G34680.1 | 269 | 2 | Chr4 | 16203127 - 16204037 |
| 317 | Ler0 | I | AtGATA3b | ATLr4G34680.2 | 269 | 2 | Chr4 | 16203127 - 16204037 |
| 318 | Ler0 | I | AtGATA7 | ATLr4G36240.1 | 238 | 2 | Chr4 | 16795292 - 16796114 |
| 319 | Ler0 | II | AtGATA19 | ATLr4G36620.1 | 211 | 2 | Chr4 | 16917009 - 16917765 |
| 320 | Ler0 | I | AtGATA12 | ATLr5G25830.1 | 332 | 2 | Chr5 | 8896793 - 8897900 |
| 321 | Ler0 | II | AtGATA23 | ATLr5G26930.1 | 103 | 2 | Chr5 | 9356793 - 9357211 |
| 322 | Ler0 | IV | AtGATA27 | ATLr5G47140.1 | 470 | 7 | Chr5 | 18755629 - 18757830 |
| 323 | Ler0 | II | AtGATA16 | ATLr5G49300.1 | 139 | 3 | Chr5 | 19589127 - 19589753 |
| 324 | Ler0 | II | AtGATA21 | ATLr5G56860.1 | 396 | 3 | Chr5 | 22565067 - 22566782 |
| 325 | Ler0 | I | AtGATA5b | ATLr5G66320.1 | 339 | 2 | Chr5 | 26059695 - 26060796 |
| 326 | Ler0 | I | AtGATA5a | ATLr5G66320.2 | 339 | 2 | Chr5 | 26059695 - 26060796 |
| 327 | Mt0 | I | AtGATA10b | ATMt1G08000.1 | 308 | 2 | Chr1 | 2470582 - 2471686 |
| 328 | Mt0 | I | AtGATA10a | ATMt1G08000.2 | 308 | 2 | Chr1 | 2470582 - 2471686 |
| 329 | Mt0 | I | AtGATA11c | ATMt1G08010.1 | 303 | 2 | Chr1 | 2473334 - 2474533 |
| 330 | Mt0 | I | AtGATA11b | ATMt1G08010.2 | 303 | 2 | Chr1 | 2473334 - 2474533 |
| 331 | Mt0 | III | AtGATA28b | ATMt1G51600.1 | 302 | 7 | Chr1 | 18740595 - 18742663 |
| 332 | Mt0 | III | AtGATA28a | ATMt1G51600.2 | 302 | 7 | Chr1 | 18740595 - 18742663 |
| 333 | Mt0 | II | AtGATA20 | ATMt2G18380.1 | 208 | 2 | Chr2 | 7678965 - 7680113 |
| 334 | Mt0 | I | AtGATA13 | ATMt2G28340.1 | 291 | 2 | Chr2 | 11735135 - 11737634 |
| 335 | Mt0 | I | AtGATA2 | ATMt2G45050.1 | 264 | 2 | Chr2 | 18190630 - 18191517 |
| 336 | Mt0 | II | AtGATA15a | ATMt3G06740.1 | 150 | 3 | Chr3 | 2124189 - 2124799 |
| 337 | Mt0 | II | AtGATA17 | ATMt3G16870.1 | 190 | 2 | Chr3 | 5747282 - 5748106 |
| 338 | Mt0 | II | AtGATA29 | ATMt3G20750.1 | 208 | 3 | Chr3 | 7236309 - 7237433 |
| 339 | Mt0 | I | AtGATA1 | ATMt3G24050.1 | 274 | 2 | Chr3 | 8643034 - 8644435 |
| 340 | Mt0 | I | AtGATA14 | ATMt3G45170.1 | 192 | 2 | Chr3 | 16239528 - 16240186 |
| 341 | Mt0 | II | AtGATA18 | ATMt3G50870.1 | 292 | 2 | Chr3 | 18564149 - 18565397 |
| 342 | Mt0 | I | AtGATA6 | ATMt3G51080.1 | 312 | 2 | Chr3 | 18626893 - 18627922 |
| 343 | Mt0 | I | AtGATA8a | ATMt3G54810.1 | 322 | 2 | Chr3 | 19945516 - 19946788 |
| 344 | Mt0 | I | AtGATA8b | ATMt3G54810.2 | 322 | 2 | Chr3 | 19945516 - 19946788 |
| 345 | Mt0 | I | AtGATA4 | ATMt3G60530.1 | 240 | 2 | Chr3 | 22008705 - 22009504 |
| 346 | Mt0 | II | AtGATA30 | ATMt4G16141.1 | 197 | 2 | Chr4 | 8878397 - 8879418 |
| 347 | Mt0 | IV | AtGATA26b | ATMt4G17570.1 | 510 | 8 | Chr4 | 9498499 - 9501144 |
| 348 | Mt0 | IV | AtGATA26a | ATMt4G17570.2 | 526 | 8 | Chr4 | 9498499 - 9500814 |
| 349 | Mt0 | IV | AtGATA26c | ATMt4G17570.3 | 514 | 8 | Chr4 | 9498499 - 9501144 |
| 350 | Mt0 | III | AtGATA25b | ATMt4G24470.1 | 309 | 7 | Chr4 | 12244899 - 12246848 |
| 351 | Mt0 | III | AtGATA25c | ATMt4G24470.2 | 309 | 7 | Chr4 | 12244899 - 12246848 |
| 352 | Mt0 | III | AtGATA25a | ATMt4G24470.3 | 317 | 7 | Chr4 | 12244899 - 12246848 |
| 353 | Mt0 | II | AtGATA22 | ATMt4G26150.1 | 351 | 3 | Chr4 | 12851148 - 12852594 |
| 354 | Mt0 | I | AtGATA9 | ATMt4G32890.1 | 308 | 2 | Chr4 | 15454920 - 15455940 |
| 355 | Mt0 | I | AtGATA3a | ATMt4G34680.1 | 269 | 2 | Chr4 | 16132156 - 16133066 |
| 356 | Mt0 | I | AtGATA3b | ATMt4G34680.2 | 269 | 2 | Chr4 | 16132156 - 16133066 |
| 357 | Mt0 | I | AtGATA7 | ATMt4G36240.1 | 238 | 2 | Chr4 | 16724766 - 16725601 |
| 358 | Mt0 | II | AtGATA19 | ATMt4G36620.1 | 211 | 2 | Chr4 | 16846539 - 16847295 |
| 359 | Mt0 | I | AtGATA12 | ATMt5G25830.1 | 331 | 2 | Chr5 | 8896979 - 8898083 |
| 360 | Mt0 | II | AtGATA23 | ATMt5G26930.1 | 120 | 2 | Chr5 | 9357739 - 9358208 |
| 361 | Mt0 | IV | AtGATA27 | ATMt5G47140.1 | 470 | 7 | Chr5 | 18810782 - 18812983 |
| 362 | Mt0 | II | AtGATA16 | ATMt5G49300.1 | 139 | 3 | Chr5 | 19638643 - 19639269 |
| 363 | Mt0 | II | AtGATA21 | ATMt5G56860.1 | 398 | 3 | Chr5 | 22614257 - 22615978 |
| 364 | Mt0 | I | AtGATA5b | ATMt5G66320.1 | 339 | 2 | Chr5 | 26107717 - 26108818 |
| 365 | Mt0 | I | AtGATA5a | ATMt5G66320.2 | 339 | 2 | Chr5 | 26107717 - 26108818 |
| 366 | No0 | I | AtGATA10b | ATNo1G08000.1 | 308 | 2 | Chr1 | 2481548 - 2482652 |
| 367 | No0 | I | AtGATA10a | ATNo1G08000.2 | 308 | 2 | Chr1 | 2481548 - 2482652 |
| 368 | No0 | I | AtGATA11c | ATNo1G08010.1 | 303 | 2 | Chr1 | 2484300 - 2485499 |
| 369 | No0 | I | AtGATA11b | ATNo1G08010.2 | 303 | 2 | Chr1 | 2484300 - 2485499 |
| 370 | No0 | III | AtGATA28b | ATNo1G51600.1 | 302 | 7 | Chr1 | 18829536 - 18831612 |
| 371 | No0 | III | AtGATA28a | ATNo1G51600.2 | 302 | 7 | Chr1 | 18829536 - 18831612 |
| 372 | No0 | II | AtGATA20 | ATNo2G18380.1 | 208 | 2 | Chr2 | 7739834 - 7740983 |
| 373 | No0 | I | AtGATA13 | ATNo2G28340.1 | 291 | 2 | Chr2 | 11815149 - 11817728 |
| 374 | No0 | I | AtGATA2 | ATNo2G45050.1 | 264 | 2 | Chr2 | 18245066 - 18245953 |
| 375 | No0 | II | AtGATA15a | ATNo3G06740.1 | 150 | 3 | Chr3 | 2126117 - 2126727 |
| 376 | No0 | II | AtGATA17 | ATNo3G16870.1 | 190 | 2 | Chr3 | 5749348 - 5750172 |
| 377 | No0 | II | AtGATA29 | ATNo3G20750.1 | 208 | 3 | Chr3 | 7234018 - 7235142 |
| 378 | No0 | III | AtGATA24a | ATNo3G21175.1 | 297 | 7 | Chr3 | 7395967 - 7397734 |
| 379 | No0 | III | AtGATA24b | ATNo3G21175.2 | 295 | 7 | Chr3 | 7395967 - 7397734 |
| 380 | No0 | I | AtGATA1 | ATNo3G24050.1 | 274 | 2 | Chr3 | 8648470 - 8649873 |
| 381 | No0 | I | AtGATA14 | ATNo3G45170.1 | 192 | 2 | Chr3 | 16223288 - 16223946 |
| 382 | No0 | II | AtGATA18 | ATNo3G50870.1 | 295 | 2 | Chr3 | 18549933 - 18551190 |
| 383 | No0 | I | AtGATA6 | ATNo3G51080.1 | 312 | 2 | Chr3 | 18612716 - 18613745 |
| 384 | No0 | I | AtGATA8a | ATNo3G54810.1 | 322 | 2 | Chr3 | 19930847 - 19932119 |
| 385 | No0 | I | AtGATA8b | ATNo3G54810.2 | 322 | 2 | Chr3 | 19930847 - 19932119 |
| 386 | No0 | I | AtGATA4 | ATNo3G60530.1 | 281 | 2 | Chr3 | 21992141 - 21993063 |
| 387 | No0 | II | AtGATA30 | ATNo4G16141.1 | 197 | 2 | Chr4 | 8874934 - 8875979 |
| 388 | No0 | IV | AtGATA26b | ATNo4G17570.1 | 510 | 8 | Chr4 | 9516603 - 9519248 |
| 389 | No0 | IV | AtGATA26a | ATNo4G17570.2 | 526 | 8 | Chr4 | 9516603 - 9518918 |
| 390 | No0 | IV | AtGATA26c | ATNo4G17570.3 | 514 | 8 | Chr4 | 9516603 - 9519248 |
| 391 | No0 | III | AtGATA25b | ATNo4G24470.1 | 309 | 7 | Chr4 | 12344373 - 12346322 |
| 392 | No0 | III | AtGATA25c | ATNo4G24470.2 | 309 | 7 | Chr4 | 12344373 - 12346322 |
| 393 | No0 | III | AtGATA25a | ATNo4G24470.3 | 317 | 7 | Chr4 | 12344373 - 12346322 |
| 394 | No0 | II | AtGATA22 | ATNo4G26150.1 | 348 | 3 | Chr4 | 12949062 - 12950502 |
| 395 | No0 | I | AtGATA9 | ATNo4G32890.1 | 308 | 2 | Chr4 | 15561652 - 15562669 |
| 396 | No0 | I | AtGATA3a | ATNo4G34680.1 | 269 | 2 | Chr4 | 16239714 - 16240624 |
| 397 | No0 | I | AtGATA3b | ATNo4G34680.2 | 269 | 2 | Chr4 | 16239714 - 16240624 |
| 398 | No0 | I | AtGATA7 | ATNo4G36240.1 | 238 | 2 | Chr4 | 16831081 - 16831916 |
| 399 | No0 | II | AtGATA19 | ATNo4G36620.1 | 211 | 2 | Chr4 | 16953140 - 16953896 |
| 400 | No0 | I | AtGATA12 | ATNo5G25830.1 | 331 | 2 | Chr5 | 8891198 - 8892302 |
| 401 | No0 | II | AtGATA23 | ATNo5G26930.1 | 120 | 2 | Chr5 | 9363208 - 9363677 |
| 402 | No0 | IV | AtGATA27 | ATNo5G47140.1 | 470 | 7 | Chr5 | 18737449 - 18739650 |
| 403 | No0 | II | AtGATA16 | ATNo5G49300.1 | 139 | 3 | Chr5 | 19556918 - 19557544 |
| 404 | No0 | II | AtGATA21 | ATNo5G56860.1 | 398 | 3 | Chr5 | 22519841 - 22521562 |
| 405 | No0 | I | AtGATA5b | ATNo5G66320.1 | 339 | 2 | Chr5 | 26014719 - 26015820 |
| 406 | No0 | I | AtGATA5a | ATNo5G66320.2 | 339 | 2 | Chr5 | 26014719 - 26015820 |
| 407 | Oy0 | I | AtGATA10b | ATOy1G08000.1 | 308 | 2 | Chr1 | 2474080 - 2475184 |
| 408 | Oy0 | I | AtGATA10a | ATOy1G08000.2 | 308 | 2 | Chr1 | 2474080 - 2475184 |
| 409 | Oy0 | I | AtGATA11c | ATOy1G08010.1 | 303 | 2 | Chr1 | 2476822 - 2478021 |
| 410 | Oy0 | I | AtGATA11b | ATOy1G08010.2 | 303 | 2 | Chr1 | 2476822 - 2478021 |
| 411 | Oy0 | III | AtGATA28b | ATOy1G51600.1 | 302 | 7 | Chr1 | 18647201 - 18649277 |
| 412 | Oy0 | III | AtGATA28a | ATOy1G51600.2 | 302 | 7 | Chr1 | 18647201 - 18649277 |
| 413 | Oy0 | II | AtGATA20 | ATOy2G18380.1 | 208 | 2 | Chr2 | 7705812 - 7706961 |
| 414 | Oy0 | I | AtGATA13 | ATOy2G28340.1 | 291 | 2 | Chr2 | 11794186 - 11796767 |
| 415 | Oy0 | I | AtGATA2 | ATOy2G45050.1 | 264 | 2 | Chr2 | 18241180 - 18242067 |
| 416 | Oy0 | II | AtGATA15a | ATOy3G06740.1 | 150 | 3 | Chr3 | 2125961 - 2126571 |
| 417 | Oy0 | II | AtGATA17 | ATOy3G16870.1 | 190 | 2 | Chr3 | 5736387 - 5737211 |
| 418 | Oy0 | II | AtGATA29 | ATOy3G20750.1 | 208 | 3 | Chr3 | 7223184 - 7224308 |
| 419 | Oy0 | III | AtGATA24a | ATOy3G21175.1 | 297 | 7 | Chr3 | 7390445 - 7392211 |
| 420 | Oy0 | III | AtGATA24b | ATOy3G21175.2 | 295 | 7 | Chr3 | 7390445 - 7392211 |
| 421 | Oy0 | I | AtGATA1 | ATOy3G24050.1 | 274 | 2 | Chr3 | 8647656 - 8649058 |
| 422 | Oy0 | I | AtGATA14 | ATOy3G45170.1 | 192 | 2 | Chr3 | 16188674 - 16189332 |
| 423 | Oy0 | II | AtGATA18 | ATOy3G50870.1 | 295 | 2 | Chr3 | 18532055 - 18533312 |
| 424 | Oy0 | I | AtGATA6 | ATOy3G51080.1 | 312 | 2 | Chr3 | 18594285 - 18595314 |
| 425 | Oy0 | I | AtGATA8a | ATOy3G54810.1 | 322 | 2 | Chr3 | 19908944 - 19910218 |
| 426 | Oy0 | I | AtGATA8b | ATOy3G54810.2 | 322 | 2 | Chr3 | 19908944 - 19910218 |
| 427 | Oy0 | I | AtGATA4 | ATOy3G60530.1 | 240 | 2 | Chr3 | 21973776 - 21974575 |
| 428 | Oy0 | II | AtGATA30 | ATOy4G16141.1 | 197 | 2 | Chr4 | 8886043 - 8887064 |
| 429 | Oy0 | IV | AtGATA26b | ATOy4G17570.1 | 510 | 8 | Chr4 | 9527420 - 9530064 |
| 430 | Oy0 | IV | AtGATA26a | ATOy4G17570.2 | 526 | 8 | Chr4 | 9527420 - 9529735 |
| 431 | Oy0 | IV | AtGATA26c | ATOy4G17570.3 | 514 | 8 | Chr4 | 9527420 - 9530064 |
| 432 | Oy0 | III | AtGATA25b | ATOy4G24470.1 | 309 | 7 | Chr4 | 12352037 - 12353977 |
| 433 | Oy0 | III | AtGATA25c | ATOy4G24470.2 | 309 | 7 | Chr4 | 12352037 - 12353977 |
| 434 | Oy0 | III | AtGATA25a | ATOy4G24470.3 | 317 | 7 | Chr4 | 12352037 - 12353977 |
| 435 | Oy0 | II | AtGATA22 | ATOy4G26150.1 | 352 | 3 | Chr4 | 12958336 - 12959785 |
| 436 | Oy0 | I | AtGATA9 | ATOy4G32890.1 | 308 | 2 | Chr4 | 15577790 - 15578807 |
| 437 | Oy0 | I | AtGATA3a | ATOy4G34680.1 | 269 | 2 | Chr4 | 16255912 - 16256822 |
| 438 | Oy0 | I | AtGATA3b | ATOy4G34680.2 | 269 | 2 | Chr4 | 16255912 - 16256822 |
| 439 | Oy0 | I | AtGATA7 | ATOy4G36240.1 | 238 | 2 | Chr4 | 16848426 - 16849261 |
| 440 | Oy0 | II | AtGATA19 | ATOy4G36620.1 | 211 | 2 | Chr4 | 16969614 - 16970370 |
| 441 | Oy0 | I | AtGATA12 | ATOy5G25830.1 | 332 | 2 | Chr5 | 8917461 - 8918568 |
| 442 | Oy0 | II | AtGATA23 | ATOy5G26930.1 | 120 | 2 | Chr5 | 9378635 - 9379104 |
| 443 | Oy0 | IV | AtGATA27 | ATOy5G47140.1 | 470 | 7 | Chr5 | 18819050 - 18821251 |
| 444 | Oy0 | II | AtGATA16 | ATOy5G49300.1 | 139 | 3 | Chr5 | 19644634 - 19645260 |
| 445 | Oy0 | II | AtGATA21 | ATOy5G56860.1 | 398 | 3 | Chr5 | 22628584 - 22630305 |
| 446 | Oy0 | I | AtGATA5b | ATOy5G66320.1 | 339 | 2 | Chr5 | 26121061 - 26122162 |
| 447 | Oy0 | I | AtGATA5a | ATOy5G66320.2 | 339 | 2 | Chr5 | 26121061 - 26122162 |
| 448 | Po0 | I | AtGATA10b | ATPo1G08000.1 | 308 | 2 | Chr1 | 2478555 - 2479659 |
| 449 | Po0 | I | AtGATA10a | ATPo1G08000.2 | 308 | 2 | Chr1 | 2478555 - 2479659 |
| 450 | Po0 | I | AtGATA11c | ATPo1G08010.1 | 303 | 2 | Chr1 | 2481297 - 2482496 |
| 451 | Po0 | I | AtGATA11b | ATPo1G08010.2 | 303 | 2 | Chr1 | 2481297 - 2482496 |
| 452 | Po0 | III | AtGATA28b | ATPo1G51600.1 | 302 | 7 | Chr1 | 18879434 - 18881510 |
| 453 | Po0 | III | AtGATA28a | ATPo1G51600.2 | 302 | 7 | Chr1 | 18879434 - 18881510 |
| 454 | Po0 | II | AtGATA20 | ATPo2G18380.1 | 208 | 2 | Chr2 | 7884886 - 7886035 |
| 455 | Po0 | I | AtGATA13 | ATPo2G28340.1 | 291 | 2 | Chr2 | 11976839 - 11979339 |
| 456 | Po0 | I | AtGATA2 | ATPo2G45050.1 | 265 | 2 | Chr2 | 18408213 - 18409103 |
| 457 | Po0 | II | AtGATA15a | ATPo3G06740.1 | 150 | 3 | Chr3 | 2128065 - 2128675 |
| 458 | Po0 | II | AtGATA17 | ATPo3G16870.1 | 190 | 2 | Chr3 | 5762313 - 5763137 |
| 459 | Po0 | II | AtGATA29 | ATPo3G20750.1 | 208 | 3 | Chr3 | 7253133 - 7254257 |
| 460 | Po0 | III | AtGATA24a | ATPo3G21175.1 | 297 | 7 | Chr3 | 7420965 - 7422731 |
| 461 | Po0 | III | AtGATA24b | ATPo3G21175.2 | 295 | 7 | Chr3 | 7420965 - 7422731 |
| 462 | Po0 | I | AtGATA1 | ATPo3G24050.1 | 274 | 2 | Chr3 | 8678991 - 8680393 |
| 463 | Po0 | I | AtGATA14 | ATPo3G45170.1 | 192 | 2 | Chr3 | 16315914 - 16316572 |
| 464 | Po0 | II | AtGATA18 | ATPo3G50870.1 | 295 | 2 | Chr3 | 18637206 - 18638463 |
| 465 | Po0 | I | AtGATA6 | ATPo3G51080.1 | 312 | 2 | Chr3 | 18699750 - 18700779 |
| 466 | Po0 | I | AtGATA8a | ATPo3G54810.1 | 322 | 2 | Chr3 | 20019651 - 20020925 |
| 467 | Po0 | I | AtGATA8b | ATPo3G54810.2 | 322 | 2 | Chr3 | 20019651 - 20020925 |
| 468 | Po0 | I | AtGATA4 | ATPo3G60530.1 | 240 | 2 | Chr3 | 22084485 - 22085284 |
| 469 | Po0 | II | AtGATA30 | ATPo4G16141.1 | 197 | 2 | Chr4 | 9003962 - 9004983 |
| 470 | Po0 | IV | AtGATA26b | ATPo4G17570.1 | 510 | 8 | Chr4 | 9643679 - 9646323 |
| 471 | Po0 | IV | AtGATA26a | ATPo4G17570.2 | 526 | 8 | Chr4 | 9643679 - 9645994 |
| 472 | Po0 | IV | AtGATA26c | ATPo4G17570.3 | 514 | 8 | Chr4 | 9643679 - 9646323 |
| 473 | Po0 | III | AtGATA25b | ATPo4G24470.1 | 309 | 7 | Chr4 | 12491706 - 12493646 |
| 474 | Po0 | III | AtGATA25c | ATPo4G24470.2 | 309 | 7 | Chr4 | 12491706 - 12493646 |
| 475 | Po0 | III | AtGATA25a | ATPo4G24470.3 | 317 | 7 | Chr4 | 12491706 - 12493646 |
| 476 | Po0 | II | AtGATA22 | ATPo4G26150.1 | 351 | 3 | Chr4 | 13098471 - 13099917 |
| 477 | Po0 | I | AtGATA9 | ATPo4G32890.1 | 308 | 2 | Chr4 | 15716691 - 15717708 |
| 478 | Po0 | I | AtGATA3a | ATPo4G34680.1 | 269 | 2 | Chr4 | 16394779 - 16395689 |
| 479 | Po0 | I | AtGATA3b | ATPo4G34680.2 | 269 | 2 | Chr4 | 16394779 - 16395689 |
| 480 | Po0 | I | AtGATA7 | ATPo4G36240.1 | 238 | 2 | Chr4 | 16986886 - 16987721 |
| 481 | Po0 | II | AtGATA19 | ATPo4G36620.1 | 211 | 2 | Chr4 | 17108141 - 17108897 |
| 482 | Po0 | I | AtGATA12 | ATPo5G25830.1 | 331 | 2 | Chr5 | 8916781 - 8917885 |
| 483 | Po0 | II | AtGATA23 | ATPo5G26930.1 | 120 | 2 | Chr5 | 9377958 - 9378427 |
| 484 | Po0 | IV | AtGATA27 | ATPo5G47140.1 | 470 | 7 | Chr5 | 18935257 - 18937458 |
| 485 | Po0 | II | AtGATA16 | ATPo5G49300.1 | 139 | 3 | Chr5 | 19766382 - 19767008 |
| 486 | Po0 | II | AtGATA21 | ATPo5G56860.1 | 399 | 3 | Chr5 | 22756781 - 22758505 |
| 487 | Po0 | I | AtGATA5b | ATPo5G66320.1 | 339 | 2 | Chr5 | 26251105 - 26252206 |
| 488 | Po0 | I | AtGATA5a | ATPo5G66320.2 | 339 | 2 | Chr5 | 26251105 - 26252206 |
| 489 | Rsch4 | I | AtGATA10b | ATRc1G08000.1 | 308 | 2 | Chr1 | 2481318 - 2482423 |
| 490 | Rsch4 | I | AtGATA10a | ATRc1G08000.2 | 308 | 2 | Chr1 | 2481318 - 2482423 |
| 491 | Rsch4 | I | AtGATA11c | ATRc1G08010.1 | 303 | 2 | Chr1 | 2484067 - 2485268 |
| 492 | Rsch4 | I | AtGATA11b | ATRc1G08010.2 | 303 | 2 | Chr1 | 2484067 - 2485268 |
| 493 | Rsch4 | III | AtGATA28b | ATRc1G51600.1 | 302 | 7 | Chr1 | 18800707 - 18802783 |
| 494 | Rsch4 | III | AtGATA28a | ATRc1G51600.2 | 302 | 7 | Chr1 | 18800707 - 18802783 |
| 495 | Rsch4 | II | AtGATA20 | ATRc2G18380.1 | 208 | 2 | Chr2 | 7737015 - 7738165 |
| 496 | Rsch4 | I | AtGATA13 | ATRc2G28340.1 | 291 | 2 | Chr2 | 11815097 - 11817599 |
| 497 | Rsch4 | I | AtGATA2 | ATRc2G45050.1 | 265 | 2 | Chr2 | 18249736 - 18250626 |
| 498 | Rsch4 | II | AtGATA15a | ATRc3G06740.1 | 150 | 3 | Chr3 | 2129197 - 2129807 |
| 499 | Rsch4 | II | AtGATA17 | ATRc3G16870.1 | 190 | 2 | Chr3 | 5756093 - 5756917 |
| 500 | Rsch4 | II | AtGATA29 | ATRc3G20750.1 | 208 | 3 | Chr3 | 7245401 - 7246525 |
| 501 | Rsch4 | III | AtGATA24a | ATRc3G21175.1 | 297 | 7 | Chr3 | 7407894 - 7409653 |
| 502 | Rsch4 | III | AtGATA24b | ATRc3G21175.2 | 295 | 7 | Chr3 | 7407894 - 7409653 |
| 503 | Rsch4 | I | AtGATA1 | ATRc3G24050.1 | 274 | 2 | Chr3 | 8645965 - 8647367 |
| 504 | Rsch4 | I | AtGATA14 | ATRc3G45170.1 | 192 | 2 | Chr3 | 16313507 - 16314165 |
| 505 | Rsch4 | II | AtGATA18 | ATRc3G50870.1 | 295 | 2 | Chr3 | 18641898 - 18643157 |
| 506 | Rsch4 | I | AtGATA6 | ATRc3G51080.1 | 312 | 2 | Chr3 | 18704779 - 18705808 |
| 507 | Rsch4 | I | AtGATA8a | ATRc3G54810.1 | 322 | 2 | Chr3 | 20023498 - 20024777 |
| 508 | Rsch4 | I | AtGATA8b | ATRc3G54810.2 | 322 | 2 | Chr3 | 20023498 - 20024777 |
| 509 | Rsch4 | I | AtGATA4 | ATRc3G60530.1 | 240 | 2 | Chr3 | 22081957 - 22082756 |
| 510 | Rsch4 | II | AtGATA30 | ATRc4G16141.1 | 197 | 2 | Chr4 | 8802980 - 8804025 |
| 511 | Rsch4 | IV | AtGATA26b | ATRc4G17570.1 | 510 | 8 | Chr4 | 9431305 - 9433949 |
| 512 | Rsch4 | IV | AtGATA26a | ATRc4G17570.2 | 526 | 8 | Chr4 | 9431305 - 9433620 |
| 513 | Rsch4 | IV | AtGATA26c | ATRc4G17570.3 | 514 | 8 | Chr4 | 9431305 - 9433949 |
| 514 | Rsch4 | III | AtGATA25b | ATRc4G24470.1 | 309 | 7 | Chr4 | 12282312 - 12284261 |
| 515 | Rsch4 | III | AtGATA25c | ATRc4G24470.2 | 309 | 7 | Chr4 | 12282312 - 12284261 |
| 516 | Rsch4 | III | AtGATA25a | ATRc4G24470.3 | 317 | 7 | Chr4 | 12282312 - 12284261 |
| 517 | Rsch4 | II | AtGATA22 | ATRc4G26150.1 | 352 | 3 | Chr4 | 12889732 - 12891181 |
| 518 | Rsch4 | I | AtGATA9 | ATRc4G32890.1 | 308 | 2 | Chr4 | 15509845 - 15510862 |
| 519 | Rsch4 | I | AtGATA3a | ATRc4G34680.1 | 269 | 2 | Chr4 | 16187754 - 16188664 |
| 520 | Rsch4 | I | AtGATA3b | ATRc4G34680.2 | 269 | 2 | Chr4 | 16187754 - 16188664 |
| 521 | Rsch4 | I | AtGATA7 | ATRc4G36240.1 | 238 | 2 | Chr4 | 16781500 - 16782335 |
| 522 | Rsch4 | II | AtGATA19 | ATRc4G36620.1 | 211 | 2 | Chr4 | 16903243 - 16903999 |
| 523 | Rsch4 | I | AtGATA12 | ATRc5G25830.1 | 331 | 2 | Chr5 | 8924180 - 8925284 |
| 524 | Rsch4 | II | AtGATA23 | ATRc5G26930.1 | 120 | 2 | Chr5 | 9378332 - 9378801 |
| 525 | Rsch4 | IV | AtGATA27 | ATRc5G47140.1 | 470 | 7 | Chr5 | 18796110 - 18798311 |
| 526 | Rsch4 | II | AtGATA16 | ATRc5G49300.1 | 134 | 3 | Chr5 | 19614072 - 19614683 |
| 527 | Rsch4 | II | AtGATA21 | ATRc5G56860.1 | 398 | 3 | Chr5 | 22611022 - 22612743 |
| 528 | Rsch4 | I | AtGATA5b | ATRc5G66320.1 | 339 | 2 | Chr5 | 26105418 - 26106519 |
| 529 | Rsch4 | I | AtGATA5a | ATRc5G66320.2 | 339 | 2 | Chr5 | 26105418 - 26106519 |
| 530 | Sf2 | I | AtGATA10b | ATSf1G08000.1 | 308 | 2 | Chr1 | 2478386 - 2479490 |
| 531 | Sf2 | I | AtGATA10a | ATSf1G08000.2 | 308 | 2 | Chr1 | 2478386 - 2479490 |
| 532 | Sf2 | I | AtGATA11c | ATSf1G08010.1 | 303 | 2 | Chr1 | 2481138 - 2482337 |
| 533 | Sf2 | I | AtGATA11b | ATSf1G08010.2 | 303 | 2 | Chr1 | 2481138 - 2482337 |
| 534 | Sf2 | III | AtGATA28b | ATSf1G51600.1 | 302 | 7 | Chr1 | 18809637 - 18811713 |
| 535 | Sf2 | III | AtGATA28a | ATSf1G51600.2 | 302 | 7 | Chr1 | 18809637 - 18811713 |
| 536 | Sf2 | II | AtGATA20 | ATSf2G18380.1 | 208 | 2 | Chr2 | 7728902 - 7730051 |
| 537 | Sf2 | I | AtGATA13 | ATSf2G28340.1 | 291 | 2 | Chr2 | 11818533 - 11821033 |
| 538 | Sf2 | I | AtGATA2 | ATSf2G45050.1 | 264 | 2 | Chr2 | 18275874 - 18276761 |
| 539 | Sf2 | II | AtGATA15a | ATSf3G06740.1 | 150 | 3 | Chr3 | 2120994 - 2121604 |
| 540 | Sf2 | II | AtGATA17 | ATSf3G16870.1 | 189 | 2 | Chr3 | 5740204 - 5741025 |
| 541 | Sf2 | II | AtGATA29 | ATSf3G20750.1 | 208 | 3 | Chr3 | 7218589 - 7219713 |
| 542 | Sf2 | III | AtGATA24a | ATSf3G21175.1 | 297 | 7 | Chr3 | 7385968 - 7387734 |
| 543 | Sf2 | III | AtGATA24b | ATSf3G21175.2 | 295 | 7 | Chr3 | 7385968 - 7387734 |
| 544 | Sf2 | I | AtGATA1 | ATSf3G24050.1 | 274 | 2 | Chr3 | 8642502 - 8643905 |
| 545 | Sf2 | I | AtGATA14 | ATSf3G45170.1 | 192 | 2 | Chr3 | 16215838 - 16216496 |
| 546 | Sf2 | II | AtGATA18 | ATSf3G50870.1 | 295 | 2 | Chr3 | 18552490 - 18553747 |
| 547 | Sf2 | I | AtGATA6 | ATSf3G51080.1 | 312 | 2 | Chr3 | 18615108 - 18616137 |
| 548 | Sf2 | I | AtGATA8a | ATSf3G54810.1 | 322 | 2 | Chr3 | 19932719 - 19933991 |
| 549 | Sf2 | I | AtGATA8b | ATSf3G54810.2 | 322 | 2 | Chr3 | 19932719 - 19933991 |
| 550 | Sf2 | I | AtGATA4 | ATSf3G60530.1 | 240 | 2 | Chr3 | 21989046 - 21989845 |
| 551 | Sf2 | II | AtGATA30 | ATSf4G16141.1 | 197 | 2 | Chr4 | 8873202 - 8874223 |
| 552 | Sf2 | IV | AtGATA26b | ATSf4G17570.1 | 510 | 8 | Chr4 | 9516721 - 9519365 |
| 553 | Sf2 | IV | AtGATA26a | ATSf4G17570.2 | 526 | 8 | Chr4 | 9516721 - 9519036 |
| 554 | Sf2 | IV | AtGATA26c | ATSf4G17570.3 | 514 | 8 | Chr4 | 9516721 - 9519365 |
| 555 | Sf2 | III | AtGATA25b | ATSf4G24470.1 | 309 | 7 | Chr4 | 12364735 - 12366684 |
| 556 | Sf2 | III | AtGATA25c | ATSf4G24470.2 | 309 | 7 | Chr4 | 12364735 - 12366684 |
| 557 | Sf2 | III | AtGATA25a | ATSf4G24470.3 | 317 | 7 | Chr4 | 12364735 - 12366684 |
| 558 | Sf2 | II | AtGATA22 | ATSf4G26150.1 | 352 | 3 | Chr4 | 12967200 - 12968649 |
| 559 | Sf2 | I | AtGATA9 | ATSf4G32890.1 | 308 | 2 | Chr4 | 15574006 - 15575023 |
| 560 | Sf2 | I | AtGATA3a | ATSf4G34680.1 | 269 | 2 | Chr4 | 16250671 - 16251581 |
| 561 | Sf2 | I | AtGATA3b | ATSf4G34680.2 | 269 | 2 | Chr4 | 16250671 - 16251581 |
| 562 | Sf2 | I | AtGATA7 | ATSf4G36240.1 | 238 | 2 | Chr4 | 16843704 - 16844539 |
| 563 | Sf2 | II | AtGATA19 | ATSf4G36620.1 | 211 | 2 | Chr4 | 16965723 - 16966479 |
| 564 | Sf2 | I | AtGATA12 | ATSf5G25830.1 | 331 | 2 | Chr5 | 8900982 - 8902086 |
| 565 | Sf2 | II | AtGATA23 | ATSf5G26930.1 | 120 | 2 | Chr5 | 9357819 - 9358288 |
| 566 | Sf2 | IV | AtGATA27 | ATSf5G47140.1 | 470 | 7 | Chr5 | 18776419 - 18778620 |
| 567 | Sf2 | II | AtGATA16 | ATSf5G49300.1 | 139 | 3 | Chr5 | 19609501 - 19610127 |
| 568 | Sf2 | II | AtGATA21 | ATSf5G56860.1 | 398 | 3 | Chr5 | 22583325 - 22585046 |
| 569 | Sf2 | I | AtGATA5b | ATSf5G66320.1 | 339 | 2 | Chr5 | 26076424 - 26077525 |
| 570 | Sf2 | I | AtGATA5a | ATSf5G66320.2 | 339 | 2 | Chr5 | 26076424 - 26077525 |
| 571 | Tsu0 | I | AtGATA10b | ATTu1G08000.1 | 308 | 2 | Chr1 | 2474414 - 2475518 |
| 572 | Tsu0 | I | AtGATA10a | ATTu1G08000.2 | 308 | 2 | Chr1 | 2474414 - 2475518 |
| 573 | Tsu0 | I | AtGATA11c | ATTu1G08010.1 | 303 | 2 | Chr1 | 2477156 - 2478356 |
| 574 | Tsu0 | I | AtGATA11b | ATTu1G08010.2 | 303 | 2 | Chr1 | 2477156 - 2478356 |
| 575 | Tsu0 | III | AtGATA28b | ATTu1G51600.1 | 302 | 7 | Chr1 | 18753865 - 18755941 |
| 576 | Tsu0 | III | AtGATA28a | ATTu1G51600.2 | 302 | 7 | Chr1 | 18753865 - 18755941 |
| 577 | Tsu0 | II | AtGATA20 | ATTu2G18380.1 | 208 | 2 | Chr2 | 7704799 - 7705947 |
| 578 | Tsu0 | I | AtGATA13 | ATTu2G28340.1 | 291 | 2 | Chr2 | 11782434 - 11784933 |
| 579 | Tsu0 | I | AtGATA2 | ATTu2G45050.1 | 265 | 2 | Chr2 | 18224140 - 18225030 |
| 580 | Tsu0 | II | AtGATA15a | ATTu3G06740.1 | 150 | 3 | Chr3 | 2127933 - 2128543 |
| 581 | Tsu0 | II | AtGATA17 | ATTu3G16870.1 | 189 | 2 | Chr3 | 5739781 - 5740602 |
| 582 | Tsu0 | II | AtGATA29 | ATTu3G20750.1 | 208 | 3 | Chr3 | 7226614 - 7227738 |
| 583 | Tsu0 | III | AtGATA24a | ATTu3G21175.1 | 297 | 7 | Chr3 | 7393276 - 7395042 |
| 584 | Tsu0 | III | AtGATA24b | ATTu3G21175.2 | 295 | 7 | Chr3 | 7393276 - 7395042 |
| 585 | Tsu0 | I | AtGATA1 | ATTu3G24050.1 | 274 | 2 | Chr3 | 8628904 - 8630307 |
| 586 | Tsu0 | I | AtGATA14 | ATTu3G45170.1 | 192 | 2 | Chr3 | 16124051 - 16124712 |
| 587 | Tsu0 | II | AtGATA18 | ATTu3G50870.1 | 295 | 2 | Chr3 | 18461043 - 18462302 |
| 588 | Tsu0 | I | AtGATA6 | ATTu3G51080.1 | 312 | 2 | Chr3 | 18523708 - 18524736 |
| 589 | Tsu0 | I | AtGATA8a | ATTu3G54810.1 | 322 | 2 | Chr3 | 19842854 - 19844133 |
| 590 | Tsu0 | I | AtGATA8b | ATTu3G54810.2 | 322 | 2 | Chr3 | 19842854 - 19844133 |
| 591 | Tsu0 | I | AtGATA4 | ATTu3G60530.1 | 240 | 2 | Chr3 | 21905089 - 21905888 |
| 592 | Tsu0 | II | AtGATA30 | ATTu4G16141.1 | 197 | 2 | Chr4 | 8879769 - 8880814 |
| 593 | Tsu0 | IV | AtGATA26b | ATTu4G17570.1 | 510 | 8 | Chr4 | 9515621 - 9518264 |
| 594 | Tsu0 | IV | AtGATA26a | ATTu4G17570.2 | 526 | 8 | Chr4 | 9515621 - 9517931 |
| 595 | Tsu0 | IV | AtGATA26c | ATTu4G17570.3 | 514 | 8 | Chr4 | 9515621 - 9518264 |
| 596 | Tsu0 | III | AtGATA25b | ATTu4G24470.1 | 309 | 7 | Chr4 | 12362608 - 12364558 |
| 597 | Tsu0 | III | AtGATA25c | ATTu4G24470.2 | 309 | 7 | Chr4 | 12362608 - 12364558 |
| 598 | Tsu0 | III | AtGATA25a | ATTu4G24470.3 | 317 | 7 | Chr4 | 12362608 - 12364558 |
| 599 | Tsu0 | II | AtGATA22 | ATTu4G26150.1 | 351 | 3 | Chr4 | 12968735 - 12970181 |
| 600 | Tsu0 | I | AtGATA9 | ATTu4G32890.1 | 308 | 2 | Chr4 | 15570088 - 15571105 |
| 601 | Tsu0 | I | AtGATA3a | ATTu4G34680.1 | 269 | 2 | Chr4 | 16248048 - 16248958 |
| 602 | Tsu0 | I | AtGATA3b | ATTu4G34680.2 | 269 | 2 | Chr4 | 16248048 - 16248958 |
| 603 | Tsu0 | I | AtGATA7 | ATTu4G36240.1 | 238 | 2 | Chr4 | 16837774 - 16838609 |
| 604 | Tsu0 | II | AtGATA19 | ATTu4G36620.1 | 209 | 2 | Chr4 | 16959359 - 16960109 |
| 605 | Tsu0 | I | AtGATA12 | ATTu5G25830.1 | 331 | 2 | Chr5 | 8895799 - 8896903 |
| 606 | Tsu0 | II | AtGATA23 | ATTu5G26930.1 | 120 | 2 | Chr5 | 9360140 - 9360609 |
| 607 | Tsu0 | IV | AtGATA27 | ATTu5G47140.1 | 470 | 7 | Chr5 | 18835841 - 18838042 |
| 608 | Tsu0 | II | AtGATA16 | ATTu5G49300.1 | 139 | 3 | Chr5 | 19654457 - 19655083 |
| 609 | Tsu0 | II | AtGATA21 | ATTu5G56860.1 | 398 | 3 | Chr5 | 22627399 - 22629120 |
| 610 | Tsu0 | I | AtGATA5b | ATTu5G66320.1 | 339 | 2 | Chr5 | 26122190 - 26123291 |
| 611 | Tsu0 | I | AtGATA5a | ATTu5G66320.2 | 339 | 2 | Chr5 | 26122190 - 26123291 |
| 612 | Wil2 | I | AtGATA10b | ATWl1G08000.1 | 308 | 2 | Chr1 | 2477717 - 2478821 |
| 613 | Wil2 | I | AtGATA10a | ATWl1G08000.2 | 308 | 2 | Chr1 | 2477717 - 2478821 |
| 614 | Wil2 | I | AtGATA11c | ATWl1G08010.1 | 303 | 2 | Chr1 | 2480469 - 2481669 |
| 615 | Wil2 | I | AtGATA11b | ATWl1G08010.2 | 303 | 2 | Chr1 | 2480469 - 2481669 |
| 616 | Wil2 | III | AtGATA28b | ATWl1G51600.1 | 302 | 7 | Chr1 | 18754906 - 18756982 |
| 617 | Wil2 | III | AtGATA28a | ATWl1G51600.2 | 302 | 7 | Chr1 | 18754906 - 18756982 |
| 618 | Wil2 | II | AtGATA20 | ATWl2G18380.1 | 208 | 2 | Chr2 | 7708819 - 7709968 |
| 619 | Wil2 | I | AtGATA13 | ATWl2G28340.1 | 291 | 2 | Chr2 | 11792967 - 11795467 |
| 620 | Wil2 | I | AtGATA2 | ATWl2G45050.1 | 264 | 2 | Chr2 | 18220189 - 18221076 |
| 621 | Wil2 | II | AtGATA15a | ATWl3G06740.1 | 150 | 3 | Chr3 | 2121168 - 2121778 |
| 622 | Wil2 | II | AtGATA17 | ATWl3G16870.1 | 190 | 2 | Chr3 | 5731048 - 5731872 |
| 623 | Wil2 | II | AtGATA29 | ATWl3G20750.1 | 208 | 3 | Chr3 | 7216157 - 7217281 |
| 624 | Wil2 | III | AtGATA24a | ATWl3G21175.1 | 297 | 7 | Chr3 | 7383796 - 7385562 |
| 625 | Wil2 | III | AtGATA24b | ATWl3G21175.2 | 295 | 7 | Chr3 | 7383796 - 7385562 |
| 626 | Wil2 | I | AtGATA1 | ATWl3G24050.1 | 274 | 2 | Chr3 | 8632635 - 8634038 |
| 627 | Wil2 | I | AtGATA14 | ATWl3G45170.1 | 192 | 2 | Chr3 | 16171267 - 16171925 |
| 628 | Wil2 | II | AtGATA18 | ATWl3G50870.1 | 295 | 2 | Chr3 | 18498380 - 18499637 |
| 629 | Wil2 | I | AtGATA6 | ATWl3G51080.1 | 312 | 2 | Chr3 | 18561013 - 18562043 |
| 630 | Wil2 | I | AtGATA8a | ATWl3G54810.1 | 322 | 2 | Chr3 | 19874543 - 19875821 |
| 631 | Wil2 | I | AtGATA8b | ATWl3G54810.2 | 322 | 2 | Chr3 | 19874543 - 19875821 |
| 632 | Wil2 | I | AtGATA4 | ATWl3G60530.1 | 240 | 2 | Chr3 | 21930568 - 21931367 |
| 633 | Wil2 | II | AtGATA30 | ATWl4G16141.1 | 197 | 2 | Chr4 | 8789319 - 8790340 |
| 634 | Wil2 | IV | AtGATA26b | ATWl4G17570.1 | 510 | 8 | Chr4 | 9424789 - 9427436 |
| 635 | Wil2 | IV | AtGATA26a | ATWl4G17570.2 | 526 | 8 | Chr4 | 9424789 - 9427103 |
| 636 | Wil2 | IV | AtGATA26c | ATWl4G17570.3 | 514 | 8 | Chr4 | 9424789 - 9427436 |
| 637 | Wil2 | III | AtGATA25b | ATWl4G24470.1 | 309 | 7 | Chr4 | 12238600 - 12240549 |
| 638 | Wil2 | III | AtGATA25c | ATWl4G24470.2 | 309 | 7 | Chr4 | 12238600 - 12240549 |
| 639 | Wil2 | III | AtGATA25a | ATWl4G24470.3 | 317 | 7 | Chr4 | 12238600 - 12240549 |
| 640 | Wil2 | II | AtGATA22 | ATWl4G26150.1 | 351 | 3 | Chr4 | 12843956 - 12845403 |
| 641 | Wil2 | I | AtGATA9 | ATWl4G32890.1 | 308 | 2 | Chr4 | 15455850 - 15456867 |
| 642 | Wil2 | I | AtGATA3a | ATWl4G34680.1 | 269 | 2 | Chr4 | 16134253 - 16135163 |
| 643 | Wil2 | I | AtGATA3b | ATWl4G34680.2 | 269 | 2 | Chr4 | 16134253 - 16135163 |
| 644 | Wil2 | I | AtGATA7 | ATWl4G36240.1 | 238 | 2 | Chr4 | 16725162 - 16725997 |
| 645 | Wil2 | II | AtGATA19 | ATWl4G36620.1 | 211 | 2 | Chr4 | 16846625 - 16847381 |
| 646 | Wil2 | I | AtGATA12 | ATWl5G25830.1 | 331 | 2 | Chr5 | 8920656 - 8921760 |
| 647 | Wil2 | II | AtGATA23 | ATWl5G26930.1 | 120 | 2 | Chr5 | 9374449 - 9374919 |
| 648 | Wil2 | IV | AtGATA27 | ATWl5G47140.1 | 470 | 7 | Chr5 | 18840202 - 18842403 |
| 649 | Wil2 | II | AtGATA16 | ATWl5G49300.1 | 139 | 3 | Chr5 | 19651818 - 19652444 |
| 650 | Wil2 | II | AtGATA21 | ATWl5G56860.1 | 398 | 3 | Chr5 | 22625595 - 22627316 |
| 651 | Wil2 | I | AtGATA5b | ATWl5G66320.1 | 339 | 2 | Chr5 | 26116116 - 26117217 |
| 652 | Wil2 | I | AtGATA5a | ATWl5G66320.2 | 339 | 2 | Chr5 | 26116116 - 26117217 |
| 653 | Ws0 | I | AtGATA10b | ATWs1G08000.1 | 308 | 2 | Chr1 | 2476430 - 2477534 |
| 654 | Ws0 | I | AtGATA10a | ATWs1G08000.2 | 308 | 2 | Chr1 | 2476430 - 2477534 |
| 655 | Ws0 | I | AtGATA11c | ATWs1G08010.1 | 303 | 2 | Chr1 | 2479182 - 2480382 |
| 656 | Ws0 | I | AtGATA11b | ATWs1G08010.2 | 303 | 2 | Chr1 | 2479182 - 2480382 |
| 657 | Ws0 | III | AtGATA28b | ATWs1G51600.1 | 302 | 7 | Chr1 | 18830421 - 18832497 |
| 658 | Ws0 | III | AtGATA28a | ATWs1G51600.2 | 302 | 7 | Chr1 | 18830421 - 18832497 |
| 659 | Ws0 | II | AtGATA20 | ATWs2G18380.1 | 208 | 2 | Chr2 | 7699422 - 7700571 |
| 660 | Ws0 | I | AtGATA13 | ATWs2G28340.1 | 257 | 2 | Chr2 | 11771075 - 11773557 |
| 661 | Ws0 | I | AtGATA2 | ATWs2G45050.1 | 266 | 2 | Chr2 | 18201837 - 18202730 |
| 662 | Ws0 | II | AtGATA15a | ATWs3G06740.1 | 150 | 3 | Chr3 | 2125890 - 2126500 |
| 663 | Ws0 | II | AtGATA17 | ATWs3G16870.1 | 190 | 2 | Chr3 | 5746265 - 5747089 |
| 664 | Ws0 | II | AtGATA29 | ATWs3G20750.1 | 208 | 3 | Chr3 | 7230413 - 7231537 |
| 665 | Ws0 | I | AtGATA1 | ATWs3G24050.1 | 274 | 2 | Chr3 | 8638712 - 8640115 |
| 666 | Ws0 | I | AtGATA14 | ATWs3G45170.1 | 192 | 2 | Chr3 | 16280693 - 16281351 |
| 667 | Ws0 | II | AtGATA18 | ATWs3G50870.1 | 295 | 2 | Chr3 | 18630529 - 18631786 |
| 668 | Ws0 | I | AtGATA6 | ATWs3G51080.1 | 312 | 2 | Chr3 | 18693183 - 18694213 |
| 669 | Ws0 | I | AtGATA8a | ATWs3G54810.1 | 322 | 2 | Chr3 | 20014015 - 20015294 |
| 670 | Ws0 | I | AtGATA8b | ATWs3G54810.2 | 322 | 2 | Chr3 | 20014015 - 20015294 |
| 671 | Ws0 | I | AtGATA4 | ATWs3G60530.1 | 240 | 2 | Chr3 | 22064495 - 22065294 |
| 672 | Ws0 | II | AtGATA30 | ATWs4G16141.1 | 197 | 2 | Chr4 | 8895456 - 8896501 |
| 673 | Ws0 | IV | AtGATA26b | ATWs4G17570.1 | 510 | 8 | Chr4 | 9526481 - 9529129 |
| 674 | Ws0 | IV | AtGATA26a | ATWs4G17570.2 | 526 | 8 | Chr4 | 9526481 - 9528796 |
| 675 | Ws0 | IV | AtGATA26c | ATWs4G17570.3 | 514 | 8 | Chr4 | 9526481 - 9529129 |
| 676 | Ws0 | III | AtGATA25b | ATWs4G24470.1 | 309 | 7 | Chr4 | 12362799 - 12364748 |
| 677 | Ws0 | III | AtGATA25c | ATWs4G24470.2 | 309 | 7 | Chr4 | 12362799 - 12364748 |
| 678 | Ws0 | III | AtGATA25a | ATWs4G24470.3 | 317 | 7 | Chr4 | 12362799 - 12364748 |
| 679 | Ws0 | II | AtGATA22 | ATWs4G26150.1 | 352 | 3 | Chr4 | 12968854 - 12970304 |
| 680 | Ws0 | I | AtGATA9 | ATWs4G32890.1 | 308 | 2 | Chr4 | 15581935 - 15582952 |
| 681 | Ws0 | I | AtGATA3a | ATWs4G34680.1 | 269 | 2 | Chr4 | 16258779 - 16259689 |
| 682 | Ws0 | I | AtGATA3b | ATWs4G34680.2 | 269 | 2 | Chr4 | 16258779 - 16259689 |
| 683 | Ws0 | I | AtGATA7 | ATWs4G36240.1 | 238 | 2 | Chr4 | 16851344 - 16852166 |
| 684 | Ws0 | II | AtGATA19 | ATWs4G36620.1 | 211 | 2 | Chr4 | 16973205 - 16973961 |
| 685 | Ws0 | I | AtGATA12 | ATWs5G25830.1 | 332 | 2 | Chr5 | 8897507 - 8898613 |
| 686 | Ws0 | II | AtGATA23 | ATWs5G26930.1 | 120 | 2 | Chr5 | 9365079 - 9365548 |
| 687 | Ws0 | IV | AtGATA27 | ATWs5G47140.1 | 470 | 7 | Chr5 | 18796035 - 18798236 |
| 688 | Ws0 | II | AtGATA16 | ATWs5G49300.1 | 139 | 3 | Chr5 | 19609340 - 19609966 |
| 689 | Ws0 | II | AtGATA21 | ATWs5G56860.1 | 398 | 3 | Chr5 | 22578422 - 22580143 |
| 690 | Ws0 | I | AtGATA5b | ATWs5G66320.1 | 339 | 2 | Chr5 | 26074823 - 26075924 |
| 691 | Ws0 | I | AtGATA5a | ATWs5G66320.2 | 339 | 2 | Chr5 | 26074823 - 26075924 |
| 692 | Wu0 | I | AtGATA10b | ATWu1G08000.1 | 308 | 2 | Chr1 | 2475610 - 2476714 |
| 693 | Wu0 | I | AtGATA10a | ATWu1G08000.2 | 308 | 2 | Chr1 | 2475610 - 2476714 |
| 694 | Wu0 | I | AtGATA11c | ATWu1G08010.1 | 303 | 2 | Chr1 | 2478362 - 2479561 |
| 695 | Wu0 | I | AtGATA11b | ATWu1G08010.2 | 303 | 2 | Chr1 | 2478362 - 2479561 |
| 696 | Wu0 | III | AtGATA28b | ATWu1G51600.1 | 302 | 7 | Chr1 | 18739174 - 18741250 |
| 697 | Wu0 | III | AtGATA28a | ATWu1G51600.2 | 302 | 7 | Chr1 | 18739174 - 18741250 |
| 698 | Wu0 | II | AtGATA20 | ATWu2G18380.1 | 208 | 2 | Chr2 | 7758916 - 7760065 |
| 699 | Wu0 | I | AtGATA13 | ATWu2G28340.1 | 291 | 2 | Chr2 | 11835788 - 11838287 |
| 700 | Wu0 | I | AtGATA2 | ATWu2G45050.1 | 264 | 2 | Chr2 | 18273746 - 18274633 |
| 701 | Wu0 | II | AtGATA15a | ATWu3G06740.1 | 149 | 3 | Chr3 | 2120783 - 2121390 |
| 702 | Wu0 | II | AtGATA17 | ATWu3G16870.1 | 190 | 2 | Chr3 | 5743051 - 5743875 |
| 703 | Wu0 | II | AtGATA29 | ATWu3G20750.1 | 208 | 3 | Chr3 | 7226322 - 7227446 |
| 704 | Wu0 | III | AtGATA24a | ATWu3G21175.1 | 297 | 7 | Chr3 | 7394051 - 7395817 |
| 705 | Wu0 | III | AtGATA24b | ATWu3G21175.2 | 295 | 7 | Chr3 | 7394051 - 7395817 |
| 706 | Wu0 | I | AtGATA1 | ATWu3G24050.1 | 274 | 2 | Chr3 | 8643361 - 8644763 |
| 707 | Wu0 | I | AtGATA14 | ATWu3G45170.1 | 192 | 2 | Chr3 | 16205198 - 16205856 |
| 708 | Wu0 | II | AtGATA18 | ATWu3G50870.1 | 295 | 2 | Chr3 | 18531363 - 18532620 |
| 709 | Wu0 | I | AtGATA6 | ATWu3G51080.1 | 288 | 2 | Chr3 | 18593542 - 18594499 |
| 710 | Wu0 | I | AtGATA8a | ATWu3G54810.1 | 322 | 2 | Chr3 | 19911394 - 19912673 |
| 711 | Wu0 | I | AtGATA8b | ATWu3G54810.2 | 322 | 2 | Chr3 | 19911394 - 19912673 |
| 712 | Wu0 | I | AtGATA4 | ATWu3G60530.1 | 240 | 2 | Chr3 | 21974407 - 21975206 |
| 713 | Wu0 | II | AtGATA30 | ATWu4G16141.1 | 197 | 2 | Chr4 | 8917556 - 8918619 |
| 714 | Wu0 | IV | AtGATA26b | ATWu4G17570.1 | 510 | 8 | Chr4 | 9531158 - 9533806 |
| 715 | Wu0 | IV | AtGATA26a | ATWu4G17570.2 | 526 | 8 | Chr4 | 9531158 - 9533473 |
| 716 | Wu0 | IV | AtGATA26c | ATWu4G17570.3 | 514 | 8 | Chr4 | 9531158 - 9533806 |
| 717 | Wu0 | III | AtGATA25b | ATWu4G24470.1 | 309 | 7 | Chr4 | 12385992 - 12387941 |
| 718 | Wu0 | III | AtGATA25c | ATWu4G24470.2 | 309 | 7 | Chr4 | 12385992 - 12387941 |
| 719 | Wu0 | III | AtGATA25a | ATWu4G24470.3 | 317 | 7 | Chr4 | 12385992 - 12387941 |
| 720 | Wu0 | II | AtGATA22 | ATWu4G26150.1 | 351 | 3 | Chr4 | 12991600 - 12993046 |
| 721 | Wu0 | I | AtGATA9 | ATWu4G32890.1 | 308 | 2 | Chr4 | 15611952 - 15612969 |
| 722 | Wu0 | I | AtGATA3a | ATWu4G34680.1 | 269 | 2 | Chr4 | 16286164 - 16287074 |
| 723 | Wu0 | I | AtGATA3b | ATWu4G34680.2 | 269 | 2 | Chr4 | 16286164 - 16287074 |
| 724 | Wu0 | I | AtGATA7 | ATWu4G36240.1 | 238 | 2 | Chr4 | 16878915 - 16879750 |
| 725 | Wu0 | II | AtGATA19 | ATWu4G36620.1 | 211 | 2 | Chr4 | 17000895 - 17001651 |
| 726 | Wu0 | I | AtGATA12 | ATWu5G25830.1 | 332 | 2 | Chr5 | 8894239 - 8895346 |
| 727 | Wu0 | II | AtGATA23 | ATWu5G26930.1 | 120 | 2 | Chr5 | 9363817 - 9364286 |
| 728 | Wu0 | IV | AtGATA27 | ATWu5G47140.1 | 470 | 7 | Chr5 | 18789771 - 18791972 |
| 729 | Wu0 | II | AtGATA16 | ATWu5G49300.1 | 139 | 3 | Chr5 | 19615627 - 19616253 |
| 730 | Wu0 | II | AtGATA21 | ATWu5G56860.1 | 399 | 3 | Chr5 | 22596514 - 22598238 |
| 731 | Wu0 | I | AtGATA5b | ATWu5G66320.1 | 339 | 2 | Chr5 | 26087952 - 26089053 |
| 732 | Wu0 | I | AtGATA5a | ATWu5G66320.2 | 339 | 2 | Chr5 | 26087952 - 26089053 |
| 733 | Zu0 | I | AtGATA10b | ATZu1G08000.1 | 308 | 2 | Chr1 | 2472223 - 2473327 |
| 734 | Zu0 | I | AtGATA10a | ATZu1G08000.2 | 308 | 2 | Chr1 | 2472223 - 2473327 |
| 735 | Zu0 | I | AtGATA11c | ATZu1G08010.1 | 303 | 2 | Chr1 | 2474965 - 2476165 |
| 736 | Zu0 | I | AtGATA11b | ATZu1G08010.2 | 303 | 2 | Chr1 | 2474965 - 2476165 |
| 737 | Zu0 | III | AtGATA28b | ATZu1G51600.1 | 302 | 7 | Chr1 | 18845540 - 18847616 |
| 738 | Zu0 | III | AtGATA28a | ATZu1G51600.2 | 302 | 7 | Chr1 | 18845540 - 18847616 |
| 739 | Zu0 | II | AtGATA20 | ATZu2G18380.1 | 208 | 2 | Chr2 | 7680627 - 7681776 |
| 740 | Zu0 | I | AtGATA13 | ATZu2G28340.1 | 291 | 2 | Chr2 | 11741555 - 11744136 |
| 741 | Zu0 | I | AtGATA2 | ATZu2G45050.1 | 265 | 2 | Chr2 | 18187337 - 18188227 |
| 742 | Zu0 | II | AtGATA15a | ATZu3G06740.1 | 150 | 3 | Chr3 | 2126202 - 2126812 |
| 743 | Zu0 | II | AtGATA17 | ATZu3G16870.1 | 189 | 2 | Chr3 | 5750990 - 5751811 |
| 744 | Zu0 | II | AtGATA29 | ATZu3G20750.1 | 208 | 3 | Chr3 | 7236893 - 7238017 |
| 745 | Zu0 | III | AtGATA24a | ATZu3G21175.1 | 297 | 7 | Chr3 | 7404628 - 7406394 |
| 746 | Zu0 | III | AtGATA24b | ATZu3G21175.2 | 295 | 7 | Chr3 | 7404628 - 7406394 |
| 747 | Zu0 | I | AtGATA1 | ATZu3G24050.1 | 274 | 2 | Chr3 | 8663772 - 8665174 |
| 748 | Zu0 | I | AtGATA14 | ATZu3G45170.1 | 192 | 2 | Chr3 | 16286549 - 16287207 |
| 749 | Zu0 | II | AtGATA18 | ATZu3G50870.1 | 296 | 2 | Chr3 | 18625041 - 18626301 |
| 750 | Zu0 | I | AtGATA6 | ATZu3G51080.1 | 312 | 2 | Chr3 | 18687864 - 18688893 |
| 751 | Zu0 | I | AtGATA8a | ATZu3G54810.1 | 322 | 2 | Chr3 | 20003683 - 20004955 |
| 752 | Zu0 | I | AtGATA8b | ATZu3G54810.2 | 322 | 2 | Chr3 | 20003683 - 20004955 |
| 753 | Zu0 | I | AtGATA4 | ATZu3G60530.1 | 244 | 2 | Chr3 | 22057753 - 22058564 |
| 754 | Zu0 | II | AtGATA30 | ATZu4G16141.1 | 197 | 2 | Chr4 | 8913271 - 8914316 |
| 755 | Zu0 | IV | AtGATA26b | ATZu4G17570.1 | 510 | 8 | Chr4 | 9564709 - 9567352 |
| 756 | Zu0 | IV | AtGATA26a | ATZu4G17570.2 | 526 | 8 | Chr4 | 9564709 - 9567019 |
| 757 | Zu0 | IV | AtGATA26c | ATZu4G17570.3 | 514 | 8 | Chr4 | 9564709 - 9567352 |
| 758 | Zu0 | III | AtGATA25b | ATZu4G24470.1 | 309 | 7 | Chr4 | 12394573 - 12396522 |
| 759 | Zu0 | III | AtGATA25c | ATZu4G24470.2 | 309 | 7 | Chr4 | 12394573 - 12396522 |
| 760 | Zu0 | III | AtGATA25a | ATZu4G24470.3 | 317 | 7 | Chr4 | 12394573 - 12396522 |
| 761 | Zu0 | II | AtGATA22 | ATZu4G26150.1 | 351 | 3 | Chr4 | 12999886 - 13001332 |
| 762 | Zu0 | I | AtGATA9 | ATZu4G32890.1 | 308 | 2 | Chr4 | 15609797 - 15610814 |
| 763 | Zu0 | I | AtGATA3a | ATZu4G34680.1 | 269 | 2 | Chr4 | 16287946 - 16288856 |
| 764 | Zu0 | I | AtGATA3b | ATZu4G34680.2 | 269 | 2 | Chr4 | 16287946 - 16288856 |
| 765 | Zu0 | I | AtGATA7 | ATZu4G36240.1 | 238 | 2 | Chr4 | 16880502 - 16881337 |
| 766 | Zu0 | II | AtGATA19 | ATZu4G36620.1 | 211 | 2 | Chr4 | 17002467 - 17003223 |
| 767 | Zu0 | I | AtGATA12 | ATZu5G25830.1 | 331 | 2 | Chr5 | 8915672 - 8916776 |
| 768 | Zu0 | II | AtGATA23 | ATZu5G26930.1 | 120 | 2 | Chr5 | 9372336 - 9372805 |
| 769 | Zu0 | IV | AtGATA27 | ATZu5G47140.1 | 470 | 7 | Chr5 | 18721040 - 18723241 |
| 770 | Zu0 | II | AtGATA16 | ATZu5G49300.1 | 139 | 3 | Chr5 | 19551079 - 19551705 |
| 771 | Zu0 | II | AtGATA21 | ATZu5G56860.1 | 398 | 3 | Chr5 | 22525667 - 22527388 |
| 772 | Zu0 | I | AtGATA5b | ATZu5G66320.1 | 339 | 2 | Chr5 | 26022218 - 26023319 |
| 773 | Zu0 | I | AtGATA5a | ATZu5G66320.2 | 339 | 2 | Chr5 | 26022218 - 26023319 |
